# Fibroblast growth factor 18 alleviates stress-induced pathological cardiac hypertrophy in male mice

**DOI:** 10.1038/s41467-023-36895-1

**Published:** 2023-03-04

**Authors:** Gen Chen, Ning An, Jingling Shen, Huinan Chen, Yunjie Chen, Jia Sun, Zhicheng Hu, Junhui Qiu, Cheng Jin, Shengqu He, Lin Mei, Yanru Sui, Wanqian Li, Peng Chen, Xueqiang Guan, Maoping Chu, Yang Wang, Litai Jin, Kwonseop Kim, Xiaokun Li, Weitao Cong, Xu Wang

**Affiliations:** 1 School of Pharmaceutical Science, Wenzhou Medical University, Wenzhou, 325035 P.R. China; 2 Oujiang Laboratory (Zhejiang Lab for Regenerative Medicine, Vision and Brain Health), School of Pharmaceutical Science, Wenzhou Medical University, Wenzhou, P.R. China; 3 College of Pharmacy, Chonnam National University, Gwangju, 61186 Korea; 4 Department of Physiology and Pharmacology, School of Basic Medical Sciences, Health Science Center, Ningbo University, Ningbo, 315211 P.R. China; 5 Department of Pharmacy, The Affiliated Li Huili Hospital, Ningbo University, Ningbo, 315040 P.R. China; 6 Institute of Life Sciences, College of Life and Environmental Sciences, Wenzhou University, Wenzhou, 325035 P.R. China; 7 Department of Pharmacy, Ningbo First Hospital, Ningbo, 315010 P.R. China; 8 Department of Pharmacy, Xiamen Medical College, Xiamen, 361023 PR China; 9 Pharmacy Department, Taizhou Municipal Hospital, Taizhou, 318000 P.R. China; 10 Department of Cardiology, The Second Affiliated Hospital and Yuying Children’s Hospital of Wenzhou Medical University, Wenzhou, 325027 P.R. China; 11 Pediatric Research Institute, The Second Affiliated Hospital and Yuying Children’s Hospital of Wenzhou Medical University, Wenzhou, 325027 P.R. China; 12 Department of Histology and Embryology, Institute of Neuroscience, Wenzhou Medical University, Wenzhou, 325035 China

**Keywords:** Heart failure, Cardiac hypertrophy, Hypertension

## Abstract

Fibroblast growth factor-18 (FGF18) has diverse organ development and damage repair roles. However, its role in cardiac homeostasis following hypertrophic stimulation remains unknown. Here we investigate the regulation and function of the FGF18 in pressure overload (PO)-induced pathological cardiac hypertrophy. FGF18 heterozygous (*Fgf18*^+/−^) and inducible cardiomyocyte-specific *FGF18* knockout (*Fgf18-CKO*) male mice exposed to transverse aortic constriction (TAC) demonstrate exacerbated pathological cardiac hypertrophy with increased oxidative stress, cardiomyocyte death, fibrosis, and dysfunction. In contrast, cardiac-specific overexpression of FGF18 alleviates hypertrophy, decreased oxidative stress, attenuates cardiomyocyte apoptosis, and ameliorates fibrosis and cardiac function. Tyrosine-protein kinase FYN (FYN), the downstream factor of FGF18, was identified by bioinformatics analysis, LC-MS/MS and experiment validation. Mechanistic studies indicate that FGF18/FGFR3 promote FYN activity and expression and negatively regulate NADPH oxidase 4 (NOX4), thereby inhibiting reactive oxygen species (ROS) generation and alleviating pathological cardiac hypertrophy. This study uncovered the previously unknown cardioprotective effect of FGF18 mediated by the maintenance of redox homeostasis through the FYN/NOX4 signaling axis in male mice, suggesting a promising therapeutic target for the treatment of cardiac hypertrophy.

## Abstract (web summary)

Although the role of FGFs in cardiovascular disease has attracted extensive attention, the potential role of FGF18 in pathological cardiac hypertrophy remains unknown. Here, the authors show the cardioprotective effect of FGF18 is mediated by maintaining redox homeostasis through FYN/Nox4 signaling.

## Introduction

Millions of patients worldwide suffer from hypertension and its cardiovascular sequelae, cardiac hypertrophy, and congestive heart failure. Cardiac hypertrophy is an adaptive response to injury that maintains cardiac function, whereas sustained hypertrophy, which can eventually lead to heart failure, is associated with increased interstitial fibrosis, cell death, and contractile dysfunction^1–3^. There is no effective drug therapy to reverse the progression from pathological cardiac hypertrophy to heart failure. Positive intervention during the early stages of heart failure is a widely accepted strategy to inhibit pathologic cardiac hypertrophy.

In fact, increasing studies have shown that oxidative stress is rapidly triggered in cardiac pathophysiology^4^. Mechanistically, oxidative stress induced by ROS causes contractile failure and structural damage, and thus plays an essential role in the pathogenesis of heart failure, especially myocardial hypertrophy^5–8^. In the past, nonspecific scavenging of ROS by antioxidant compounds does not counteract disease. Therefore, manipulating the endogenous redox system via specific targeting is potentially beneficial for more refined redox therapy^9,10^. NADPH oxidases (NOXs) are the only known enzymes whose sole biological function is to purposefully produce O_2_^–^ or H_2_O_2_ and are major sources of ROS in the cardiovascular system^11^. Among the seven members of the NOX family of proteins (NOX1-5 and DUOX1 and 2), NOX2 and NOX4 are associated with the pathogenesis of cardiovascular diseases and serve as therapeutic targets in these diseases^12,13^.

Specifically, NOX2 is required for angiotensin II (Ang II) but not pressure overload (PO)-induced cardiac hypertrophy^14^, whereas cardiac-specific overexpression of NOX4 causes cardiac fibrosis and hypertrophy^15^. By contrast, in cardiac-specific, but not systemic, NOX4-knockout (KO) mice, cardiac hypertrophy and dysfunction in response to PO are significantly attenuated due to decreased ROS generation^16,17^. Meanwhile, FYN, a non-receptor-type tyrosine kinase, maintains cardiac homeostasis by negatively regulating NOX4 activity in cardiomyocytes and optimizing ROS production^18^. Mechanistically, phosphorylation of tyrosine 416 leads to activation of FYN, which alleviates ROS-induced cardiomyocyte injury by phosphorylating Y566 of NOX4. This suggests that FYN can be targeted to inhibit ROS overproduction and protect the myocardium. Thus, a better understanding of the potential mediators that regulate FYN activity and inhibit ROS overproduction might be clinically valuable for treating pathologic myocardial hypertrophy.

The fibroblast growth factor (FGF) family includes 23 members that function in cell growth and survival, and are involved in various biological processes, such as embryonic development, tissue repair, and angiogenesis^19,20^. FGF18, which selectively binds to FGFR3, is an essential mitogen for embryonic limb development and is required for lung development and disease^21,22^. It is also involved in differentiation during osteogenesis and chondrogenesis^23–26^. Meanwhile, sprifermin (recombinant human FGF18) has been clinically used as a disease-modifying osteoarthritis drug in phase I and Ib studies^27–29^. However, only a few studies have examined the relationship between FGF18 and cardiovascular diseases, such as FGF18 promotes tissue repair after ischemia-induced injury in a model of short-term cerebral ischemia^30^, and FGF18 levels in peripheral blood show a compensatory increase after myocardial infarction^31^. Also, the mRNA level of *FGF18* increases in pediatric dilated cardiomyopathy (DCM) hearts^32^ and hearts with ventricular tachycardia^33^. In our study, analysis of the GSE18801 dataset showed that FGF18 was significantly decreased in the myocardium under isoproterenol (ISO) treatment. We found that FGF18 was significantly higher in the heart than in other organs at 14 weeks of age, indicating that FGF18 has a potential role in the maintenance of myocardial homeostasis. Despite the importance of FGF18 as a paracrine factor, no studies have focused on its role in pathologic cardiac hypertrophy.

In the present study, we analyzed the role of FGF18 in pathologic cardiac hypertrophy. We observed that mice lacking FGF18 (*Fgf18*^+/−^*KO* and *Fgf18*-*CKO*) were prone to developing cardiac hypertrophy under stress, and overexpression of FGF18 antagonized PO- or ISO-induced cardiac hypertrophy. We also showed that FGF18/FGFR3 exerted these cardioprotective effects by phosphorylating FYN^Y416^ and negatively regulating NOX4, decreasing ROS generation. This study provides a mechanistic underpinning for clinical observations and indicates that FGF18 is a potential therapeutic factor or functional biomarker in the chronic phase of cardiac hypertrophy.

## Results

### FGF18 is downregulated in pathological cardiac hypertrophy

**Fig. 1:**
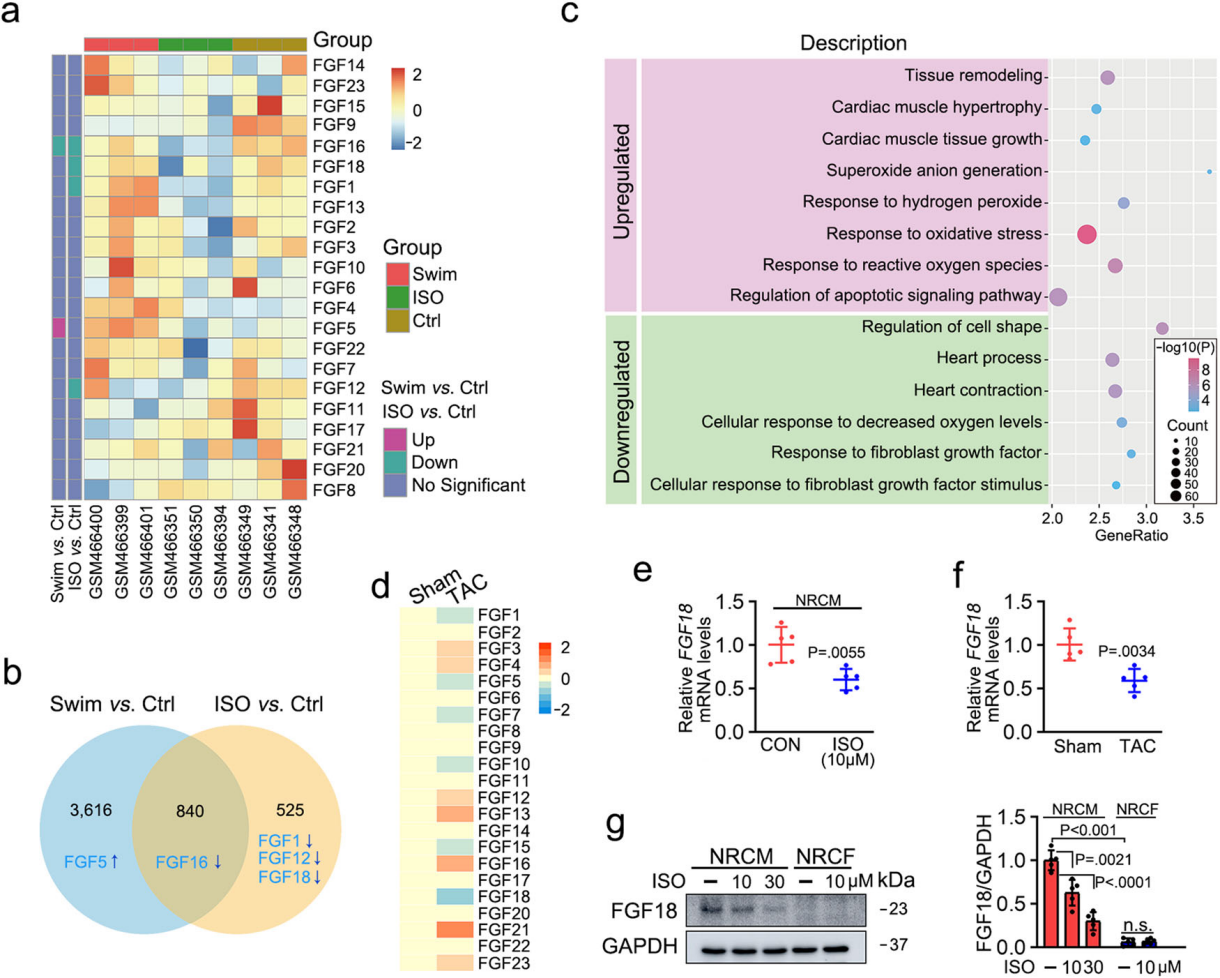
Identification of the candidate FGFs-related genes in pathological myocardial hypertrophy. **a**–**c** The results were from the analysis of the GSE18801 dataset. **a** The corresponding FGFs of the 5 genes correlated with the physiological and pathological myocardial hypertrophy were visualized by the heatmap in the normal, swim, and isoproterenol (ISO) treatment samples. The darker shade of red or blue represents the higher correlation level. **b** Venn diagram visualizing the myocardial hypertrophy-related differentially expressed genes between swim and ISO treatment. **c** KEGG enrichment analysis of the influence of pathological myocardial hypertrophy genes after ISO treatment. The y-axis refers to pathway terms. The x-axis is the rich factor. **d** A profile of the mRNA expression of FGFs in Sham or transverse aortic constriction (TAC)-operated mouse heart at 6 weeks. Heatmap depicts differentially expressed FGFs mRNA in Sham and TAC heart samples (*n* = 4/group). Two-tailed student’s t-test. **e** Real-time quantitative PCR assays were performed to determine the mRNA levels of FGF18 in neonatal rat cardiomyocytes (NRCMs). *n* = 5. Data represent means ± SD. Two-tailed student’s t-test. **f** Real-time quantitative PCR assays were performed to determine the mRNA levels of FGF18 in Sham or TAC mice. *n* = 5. Data represent means ± SD. Two-tailed student’s t-test. **g** Representative western blots; Quantitative results of FGF18 protein expression in NRCMs and neonatal rat cardiac broblasts (NRCFs) treated with ISO for 48 h. *n* = 5. Data represent means ± SD. Two-tailed student’s t-test. All numbers (*n*) are biologically independent experiments. Source data are provided as a Source data file.

To examine the correlation between the expression of *FGFs* and biological and pathological myocardial hypertrophy, the GSE18801 dataset was analyzed (hearts from the swim and ISO treatment, respectively). The hierarchical cluster heatmap and Venn diagram indicated that 5 of 22 *FGF* genes (*FGF1*, *FGF5*, *FGF12*, *FGF16*, and *FGF18*) were associated with myocardial hypertrophy (Fig. 1a, b). Detailly, we found that *FGF1*, *FGF12*, and *FGF18* were decreased in ISO-treated hearts, and *FGF16* was decreased in both swimming and ISO model groups. Interestingly, *FGF5* was elevated in swimming-induced physiological hypertrophy group (Fig. 1a, b). Although the roles and mechanisms of FGF1^34^, FGF5^35^, FGF12^36^, and FGF16^37^ in myocardial injury have been revealed successively, the role of FGF18 in pathological myocardial hypertrophy has never been reported in detail. Predominantly, FGF18 was highly expressed in the myocardium and had no significant change under physiological hypertrophy, while it is significantly decreased under ISO stimulation, suggesting that FGF18 might be involved in maintaining cardiac homeostasis. To further assess the expression profile of *FGFs* in the hypertrophic heart, wild-type (WT) mice were subjected to TAC operation. The results of RT-qPCR showed that *FGF18* was highly downregulated in the heart of TAC mice at 6 weeks (Fig. 1d, f), which was consistent with the GSE18801 dataset (Fig. 1b). Decreased protein levels of FGF18 were also obtained by immunoblotting in isolated adult mouse cardiomyocytes (ACMs) and whole heart tissue after TAC for 6 weeks (Supplementary Fig. 1a). Whether the protein levels correlated with the mRNA levels of other potential growth factors (FGF1, 2, 3, 5, 9, 13, 16) mentioned in the TAC model, consistent with previous revealed, we found that protein levels of FGF2, FGF3, FGF13, and FGF16 were increased, whereas FGF1 and FGF9 were not significantly changed (Supplementary Fig. 1b). Furthermore, the expression of several *FGFs* were affected after 48 h ISO-treatment in neonatal rat cardiomyocytes (NRCMs), including downregulated mRNA levels of *FGF18* (Fig. 1e; Supplementary Fig. 1c) and protein levels in a dose-dependent manner (Fig. 1g). Furthermore, Kyoto Encyclopedia of Genes and Genomes (KEGG) pathway analysis demonstrated that oxidative stress response and tissue remodeling signaling pathways were significantly increased after ISO treatment, accompanying by the decreasing in response to fibroblast growth factor (Fig. 1c). Overall, these results indicate that FGF18 might play a critical role in the regulation of cardiac hypertrophy, and was associated with oxidative stress.

### FGF18 overexpression attenuates ROS production and hypertrophy remodeling in NRCMs

To study the association of FGF18 with cardiac hypertrophy, NRCMs were infected with adenovirus harboring FGF18 and LacZ (control) and treated with ISO for 48 h. Overexpression of FGF18 attenuated ISO-induced cell enlargement (Supplementary Fig. 2a) and downregulated phenotypic marker genes [atrial natriuretic peptide (*ANP*), brain natriuretic peptide (*BNP*), *collagen I*, and *collagen III*] in NRCMs (Supplementary Fig. 2b). Given that FGF18 periodically activated mitogen-activated protein kinases (MAPKs) at basal level (Supplementary Fig. 3a) and inhibits downstream signaling pathways by endocytosis of FGFR^38^, in contrast, we found that ISO activated MAPKs, including p-Erk, p-JNK, and p-p38 in NRCMs compared with the controls, whereas FGF18 overexpression reversed these effects (Supplementary Fig. 2h).

In addition, KEGG pathway analysis suggested that oxidative stress played an essential role in the pathogenesis of pathological cardiac hypertrophy, whereas the response to fibroblast growth factor was decreased (Fig. 1c). Thus, we investigated the effects of FGF18 on oxidative stress in NRCMs exposed to hypertrophic stress conditions. ISO induced the generation of ROS in NRCMs, as evidenced by increased intensity of DHE fluorescence (Supplementary Fig. 2d), production of 3-nitrotyrosine (3-NT) (Supplementary Fig. 2e), a product of tyrosine oxidation, and detection of the cell-permeable redox-sensitive dye DCFH-DA at different time intervals (Supplementary Figs. 2f and 3b), thus inducing the accumulation of hydrogen peroxide (Supplementary Fig. 2g). FGF18 overexpression suppressed the effects of ISO on oxidative stress and decreased the production of superoxide (Supplementary Fig. 2d–g). In addition, overexpression of FGF18 decreased apoptosis induced by ISO in NRCMs, as indicated by the proportion of TUNEL-positive cells (Supplementary Fig. 2c) and the Bax/Bcl-2 ratio (Supplementary Fig. 2h). These results suggest the protective effect of FGF18 against cardiac hypertrophy in a redox-sensitive manner.

In view of that FGF18 selectively binds to FGFR3 among the four growth factor receptors (FGFR1, FGFR2, FGFR3, and FGFR4) expressed in cardiomyocytes, thus to activate downstream pathways^39,40^. We further examine whether the cardioprotective effect of FGF18 is receptor-mediated by treating cardiomyocytes with si-*FGFR3*. Silencing of *FGFR3* did not affect myocardial size (Supplementary Fig. 4a, b), apoptosis (Supplementary Fig. 4c), or superoxide generation (Supplementary Fig. 4d, e) in normal cardiomyocytes. However, si-*FGFR3* abolished the cardioprotective effects of FGF18 overexpression under hypertrophic stress (Supplementary Fig. 4). Taken together, these data indicate that cardioprotection of FGF18 is receptor-dependent.

### FGF18 overexpression mitigates TAC-induced oxidative stress and pathological cardiac hypertrophy

**Fig. 2:**
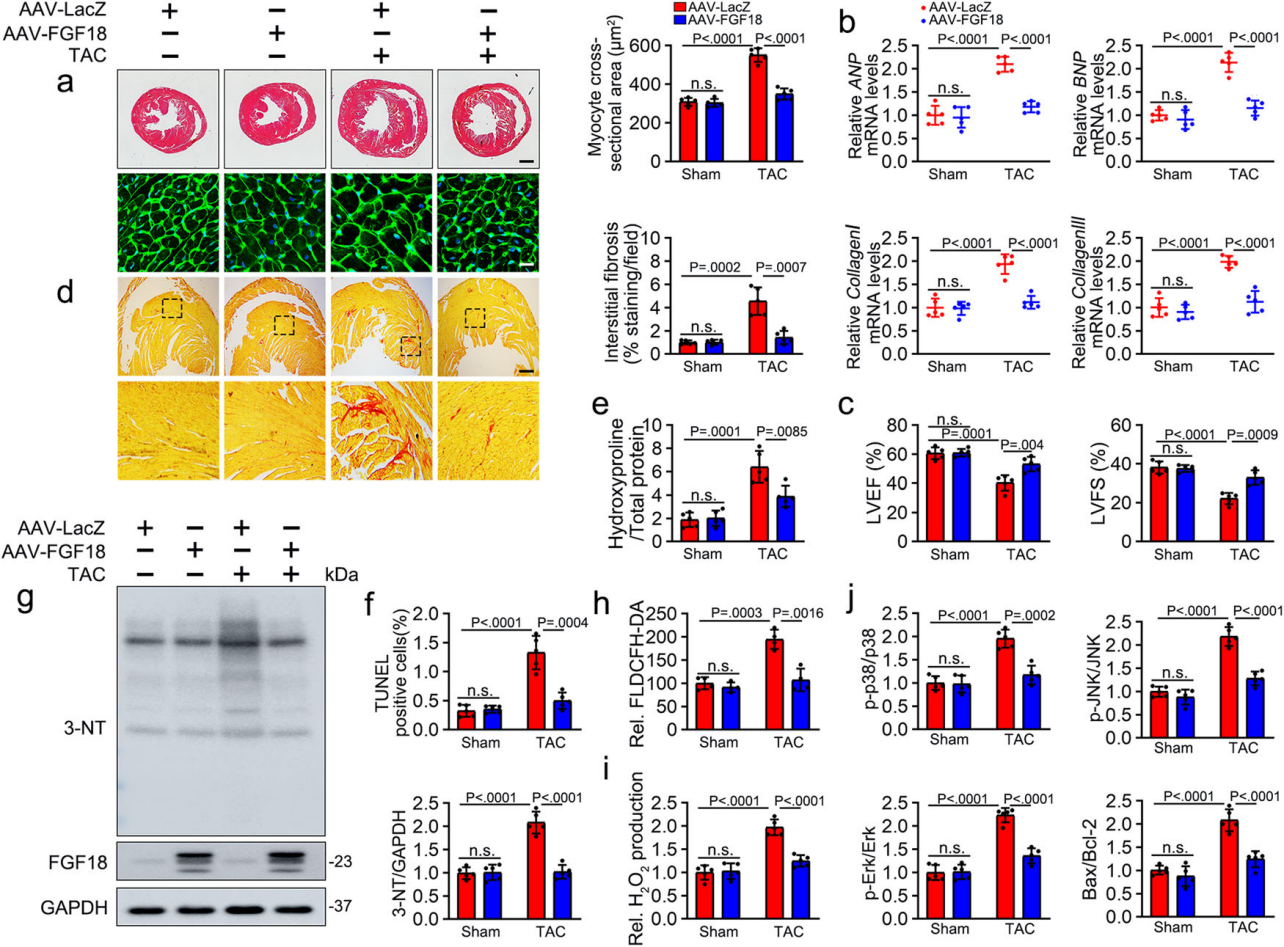
Overexpression of FGF18 attenuates pressure overload-induced cardiac hypertrophy. FGF18 overexpression vector (AAV9-cTnT-FGF18) and control vector (AAV9-LacZ) were injected intravenously into tail veins of 6 weeks old male C57BL/6J mice, respectively, 2 weeks after the injection, these mice were subjected to Sham or TAC operation. **a** Histological analysis of the Hematoxylin-eosin (HE) staining and Wheat germ agglutinin (WGA) staining (*n* = 5. Scale bar = 0.6 mm for upper HE staining; scale bar = 20 μm for lower WGA staining). Statistical results for the sectional cell area (right panel). *n* = 5. **b** Real-time quantitative PCR assays. *n* = 5. **c** Echocardiographic measurement of Left ventricular ejection fraction (LVEF) and Left ventricular fraction shortening (LVFS) are shown. *n* = 5. **d** Picrosirius red (PSR) staining and quantification (right panel). *n* = 5. Scale bar = 450 μm, and then zoom in 5 times. **e** Left ventricular collagen quantification by hydroxyproline assay (μg/mg). *n* = 5. **f** The histogram is the quantitative analysis of TUNEL^+^ cells in at least six separate fields. *n* = 5. Two-tailed non-parametric Mann–Whitney U test. **g** Levels of the oxidative damage marker 3-NT. The quantitative analysis of 3-NT protein immunoblots (right panel), *n* = 5. **h** Total reactive oxygen species (ROS) levels (by DCFH-DA probe) were quantified. *n* = 4. **i** Hydrogen peroxide levels (by Amplex Red assay) quantified in different groups. *n* = 5. One-way ANOVA was followed by a post hoc Fisher’s comparison test. **j** Heart lysate was analyzed by western blotting with indicated antibodies. Quantification of relative protein levels (right panel). *n* = 5. All quantitative data are reported as means ± SD, one-way ANOVA with Tukey multiple comparisons test: n.s. = not significant. All numbers (*n*) are biologically independent animals. Source data are provided as a Source data file.

Spatial-temporal distribution of FGF18 was investigated and illustrated that high levels of FGF18 expression were detected in the lung, liver and kidney of 4-week-old mice, whereas levels of FGF18 in those tissues were decreased significantly at 14 weeks and were lower than those in the heart (Supplementary Fig. 5a). It suggests that FGF18 might play a vital role in maintaining cardiac homeostasis in adult mice. To confirm the effect of FGF18 on pathological cardiac hypertrophy in vivo, we used adeno-associated virus (AAV9) vectors expressing FGF18 under the control of the murine cardiac troponin-T (cTNT) core promoter, which enabled specific overexpression of FGF18 in cardiomyocytes after virus injection 2 weeks and continued overexpression for 8 weeks (AAV9-cTNT-FGF18; Supplementary Fig. 5b). Consistent with the results of in vitro experiments, cardiomyocyte-specific FGF18 overexpression decreased cardiomyocyte size, as shown by hematoxylin-eosin (HE) and wheat germ agglutinin (WGA) staining (Fig. 2a), the heart weight/body weight (HW/BW) ratio (Supplementary Fig. 5c), and the mRNA level of phenotypic markers (Fig. 2b). Echocardiographic measurements showed that FGF18 overexpression dramatically alleviated TAC-induced cardiac dilation and contractile dysfunction (Fig. 2c; Supplementary Table 2). FGF18 overexpression also significantly decreased interstitial fibrosis (Fig. 2d), hydroxyproline content (Fig. 2e), and cardiomyocyte apoptosis (Fig. 2f, j; Supplementary Fig. 5e, f) compared with those in TAC mice. Consistent with these in vitro observations, MAPKs were generally activated in response to TAC and suppressed by FGF18 overexpressing (Fig. 2j; Supplementary Fig. 5f). Significantly, PO markedly increased the accumulation of 3-NT, ROS (detected by DCFH-DA) and hydrogen peroxide, and these effects were abrogated by FGF18 overexpression (Fig. 2g–i).

Furthermore, we used AAV9 vectors carrying short hairpin RNA (shRNA) (AAV9-cTNT-sh-FGFR3) to silence FGFR3 in cardiomyocytes specifically to evaluate the impact of FGFR3 on cardiac hypertrophy. Consistently, silencing of *FGFR3* did not affect myocardial hypertrophy. However, *FGFR3* knockdown abolished the cardioprotective effects of FGF18 compared with those FGF18-treated mice after TAC, including cardiomyocyte size (Supplementary Fig. 6a), myocardial fibrosis (Supplementary Fig. 6b), hydroxyproline content (Supplementary Fig. 6c) and cardiac function (Supplementary Fig. 6d, Supplementary Table 3), as well as promoting the accumulation of ROS (detected by DCFH-DA, Supplementary Fig. 6e), hydrogen peroxide (Supplementary Fig. 6f) and aggravated the expression of phenotypic markers (Supplementary Fig. 6g). These findings indicate that FGF18 attenuated PO-induced cardiac hypertrophy and oxidative stress was receptor-dependent.

### FGF18 deletion exacerbates the pathology of cardiac hypertrophy

**Fig. 3:**
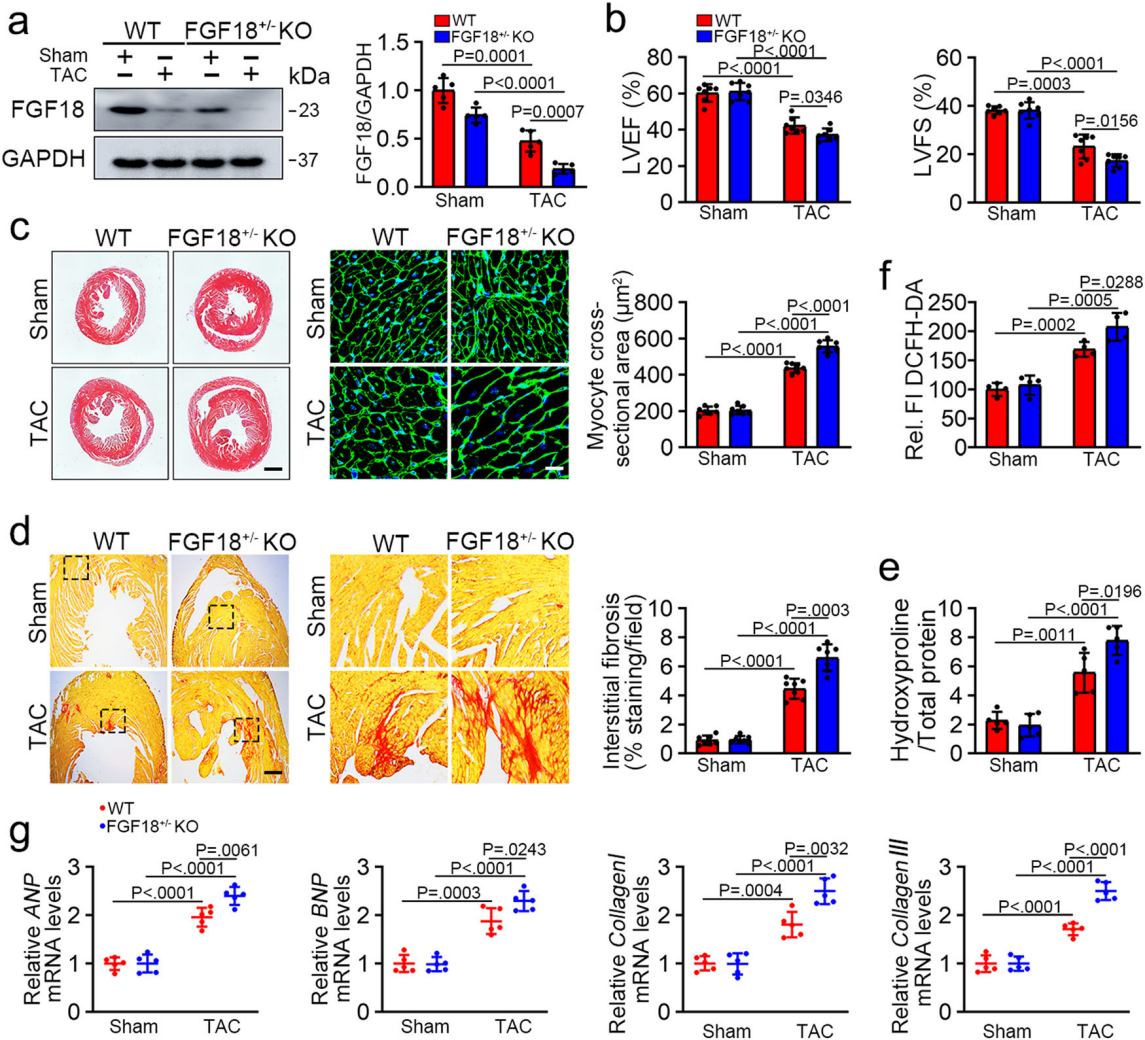
FGF18 heterozygous exacerbates the pathology of cardiac hypertrophy. FGF18 heterozygous mice (*Fgf18*^+/−^ mice) were subjected to Sham or TAC surgery and harvested after 6 weeks (Supplementary Fig. 7e). **a** Validation of FGF18 semi-knockout (*Fgf18*^+/−^*KO*) mice by western blot. Quantification of relative protein levels (right panel). *n* = 5. **b** LVEF and LVFS of WT and FGF18^+/−^*KO* mice as determined by echocardiography (Supplementary Fig. 7g). *n* = 7. **c** Histological analyses of the HE staining and WGA staining (*n* = 5. scale bar =  0.6 mm for HE staining; scale bar = 20 μm for WGA staining). Statistical results for the sectional cell area (right panel). *n* = 7. **d** PSR staining and quantification (right panel). n = 7 mice per group. Scale bar = 450 μm, and then zoom in 5 times. **e** Left ventricular collagen quantification by hydroxyproline assay (μg/mg). *n* = 5. **f** Total ROS levels (by DCFH-DA probe) were quantified. *n* = 4. One-way ANOVA followed by Turkey post-test. **g** Real-time quantitative PCR assays. *n* = 5. All quantitative data are reported as means ± SD, one-way ANOVA with Tukey multiple comparisons test. All numbers (*n*) are biologically independent animals. Source data are provided as a Source data file.

FGF18 primarily signals to mesenchymal tissue during embryonic development in developing bone and cartilage, lung and brain, and FGF18 germline KO mice (*Fgf18*^−/−^) die shortly after birth^25^. We, therefore, used FGF18 heterozygous mice (*Fgf18*^+/−^ mice, Fig. 3a, Supplementary Fig. 20a) to evaluate the impact of FGF18 on cardiac hypertrophy. A schematic of the experimental procedure is shown in Supplementary Fig. 7a. There was no difference in body weight (Supplementary Fig. 7b), organ size (Supplementary Fig. 7c), and the organ to body weight ratios (Supplementary Fig. 7d) between *Fgf*18^+/−^*KO* and WT mice. Male mice and their age-matched WT littermates were challenged with TAC operation (Supplementary Fig. 7e). Although there were no remarkable changes at the basal level, FGF18 deficiency significantly increased the heart size and HW/BW ratio (Fig. 3c and Supplementary Fig. 7f) after TAC operation. Echocardiographic observations showed that PO-induced cardiac disorders, including decreased left ventricular ejection fraction (LVEF) and left ventricular fraction shortening (LVFS), were more severe in *Fgf*18^+/−^*KO* mice than in WT controls after TAC (Fig. 3b, Supplementary Fig. 7g and Supplementary Table 4). Furthermore, picrosirius red staining showed that myocardial fibrosis (Fig. 3d) and hydroxyproline content (Fig. 3e) were exacerbated in the hearts of *Fgf*18^+/−^*KO* mice compared with those from WT controls after TAC. Also, the TAC-induced generation of ROS (Fig. 3f) and expression of phenotypic markers (Fig. 3g) were significantly increased in *Fgf*18^+/−^*KO* mice. These data suggest that FGF18 plays a protective role against cardiac hypertrophy.

### Cardiomyocyte-specific FGF18 deletion exacerbates pathological cardiac hypertrophy in vitro and in vivo

To further assess the effect of FGF18 on cardiomyocyte hypertrophy, NRCMs were transduced with adenoviruses harboring shRNA against FGF18 or a scrambled sequence in the absence or presence of ISO. Although no remarkable changes were observed at the basal level, FGF18 deficiency significantly increased cardiomyocyte size (Supplementary Fig. 8a), exacerbated apoptosis (Supplementary Fig. 8b), oxidative stress (Supplementary Fig. 8c, d), and promoted the expression of phenotypic markers (Supplementary Fig. 8e) in response to ISO. In addition, we found that FGF18 deficiency did not significantly affect the expression of other FGFs under basal conditions, however, slightly increased the expression of FGF13, FGF21, and FGF23 after ISO treatment (Supplementary Fig. 8f), suggesting that deficiency of FGF18 in response to stress exacerbates cardiac hypertrophy and indirectly affects compensatory growth factors. Together, these data demonstrate that FGF18 is protective against cardiac hypertrophy in vitro.

To study the functional significance of FGF18 expression in cardiomyocytes, we generated cardiomyocyte-specific inducible FGF18 KO mice (αMHC-MerCreMer, *Fgf18*^f/f^, *Fgf18-CKO* mice; Supplementary Figs. 9a and 20b). Cre and *Fgf18*^f/f^ alleles in mice were identified by PCR (Supplementary Fig. 9b). Adult mouse cardiomyocytes were isolated and verified by immunofluorescence (Supplementary Fig. 9c). The specificity and efficiency of FGF18 deletion were determined by real-time quantitative reverse transcription PCR and western blotting. The results showed an 80% reduction in FGF18 mRNA (Supplementary Fig. 9d) and protein levels in isolated adult mouse cardiomyocytes but not in noncardiomyocytes (endothelial cells and fibroblasts; Supplementary Fig. 9e). Immunoblot analysis showed that FGF18 protein expression decreased by 75% in whole heart tissues (Supplementary Fig. 9f), whereas no changes in FGF18 protein expression were observed in other tissues (Supplementary Fig. 9g) in *Fgf18-CKO* mice.

**Fig. 4:**
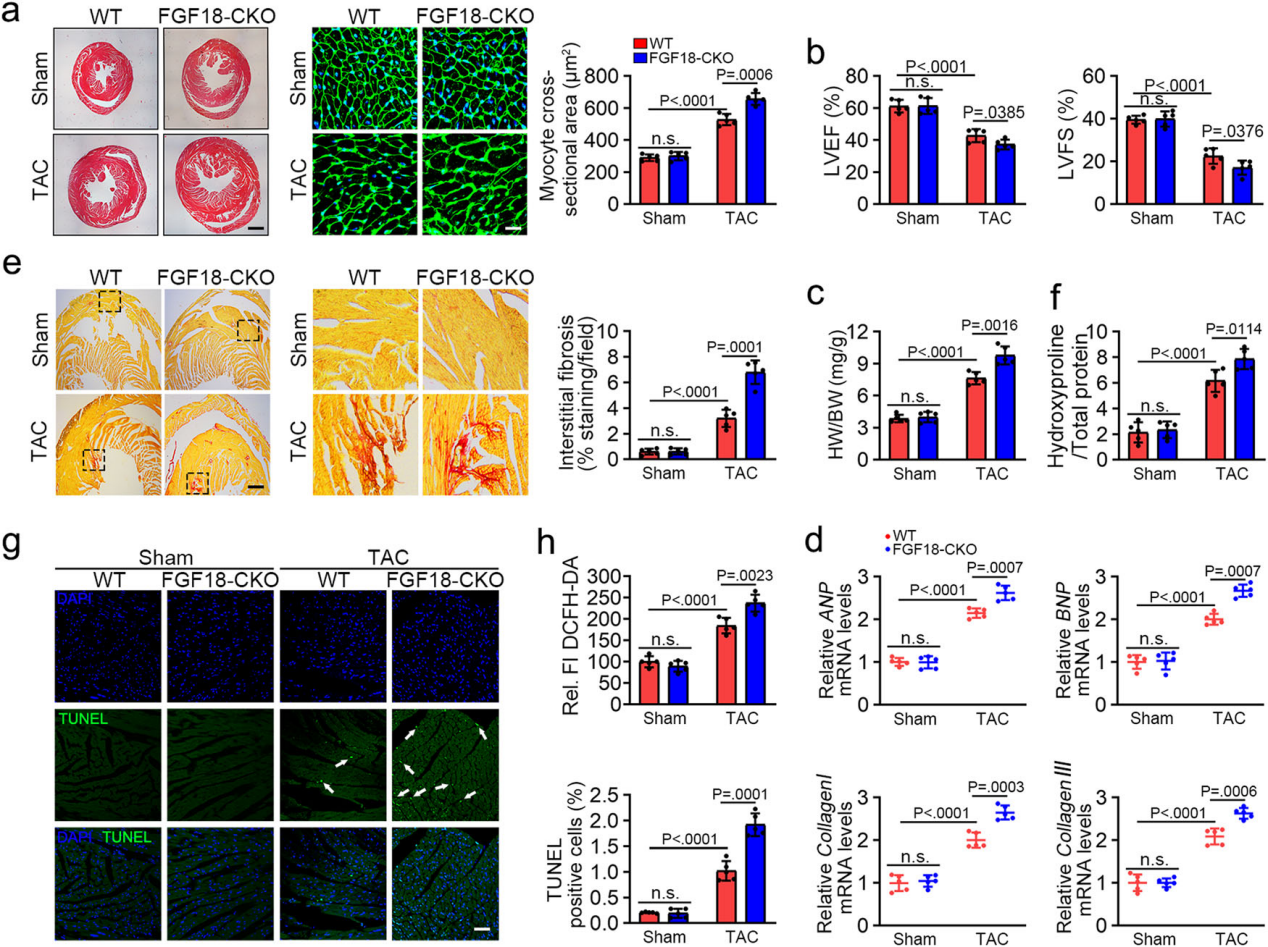
Cardiomyocyte-specific FGF18 deletion exacerbates pathological cardiac hypertrophy in vivo. *Fgf18*^f/f^ and *Fgf18*-*CKO* mice (αMHC-MerCreMer; *Fgf18*^f/f^) were intraperitoneally injected with tamoxifen at 6 weeks old male mice, and 2 weeks after the injection, these mice were subjected to Sham or TAC surgery and harvest after 6 weeks. **a** Histological analyses of the HE staining and WGA staining (*n* = 5. Scale bar = 0.6 mm for left HE staining; scale bar = 20 μm for right WGA staining). Statistical results for the sectional cell area (right panel). *n* = 5. **b** Representative echocardiographic images and echocardiographic data for LVEF and LVFS are shown. *n* = 5. **c** Statistical results for the ratios of heart weight/body weight (HW/BW) in the indicated groups. *n* = 5. **d** Real-time quantitative PCR assays. *n* = 5. **e** PSR staining and quantification (right panel). *n* = 5. Scale bar = 450 μm, and then zoom in 5 times. **f** Left ventricular collagen quantification by hydroxyproline assay (μg/mg). *n* = 5. **g** Representative confocal scans are shown for terminal TUNEL and DAPI (green and blue, respectively). The histogram (right panel) is the quantitative analysis of TUNEL^+^ cells in at least six separate fields. *n* = 5. Scale bars = 45 μm. Two-tailed Non-parametric Mann–Whitney U test. **h** Total ROS levels (by DCFH-DA probe) were quantified. *n* = 5. All quantitative data are reported as means ± SD, one-way ANOVA with Tukey multiple comparisons test: n.s. = not significant. All numbers (*n*) are biologically independent animals. Source data are provided as a Source data file.

The in vivo studies showed that the proportion of born WT (*Fgf18*^+/+^), heterozygous (αMHC-MerCreMer; *Fgf18*^f/+^), and homozygous (αMHC-MerCreMer; *Fgf18*^f/f^) mice complied with Mendel’s laws, and their appearance was indistinguishable from that of their littermates (Supplementary Fig. 9h). There were no differences in cardiac phenotype between *Fgf18-CKO* and WT mice at the basal level. However, the survival rate was significantly lower in *Fgf18-CKO* mice after TAC operation than in the α-MHC-MerCreMer and *Fgf18*^f/f^ controls (Supplementary Fig. 9i). Furthermore, the TAC-induced hypertrophic pathology was exacerbated in *Fgf18-CKO* mice, as shown by the changes in heart size and cardiomyocyte cross-sectional area (Fig. 4a, Supplementary Fig. 9j). Echocardiographic analysis showed that PO-induced cardiac disorders, including decreased LVEF, LVFS, and increased HW/BW ratio, were more severe in *Fgf18-CKO* than in WT mice after TAC (Fig. 4b, c; Supplementary Fig. 9k; Supplementary Table 5). These changes were accompanied by the increased mRNA level of phenotypic markers (Fig. 4d), aggravation of interstitial fibrosis (Fig. 4e), hydroxyproline content (Fig. 4f), myocyte apoptosis (Fig. 4g) and accumulation of oxidation products (Fig. 4h). In summary, these results strongly support the notion that cardiomyocyte-specific FGF18 deletion exacerbated TAC-induced ventricular remodeling.

### FGF18 promotes the tyrosine kinase activity and expression of FYN and attenuates cardiomyocyte hypertrophy in vitro and in vivo

**Fig. 5:**
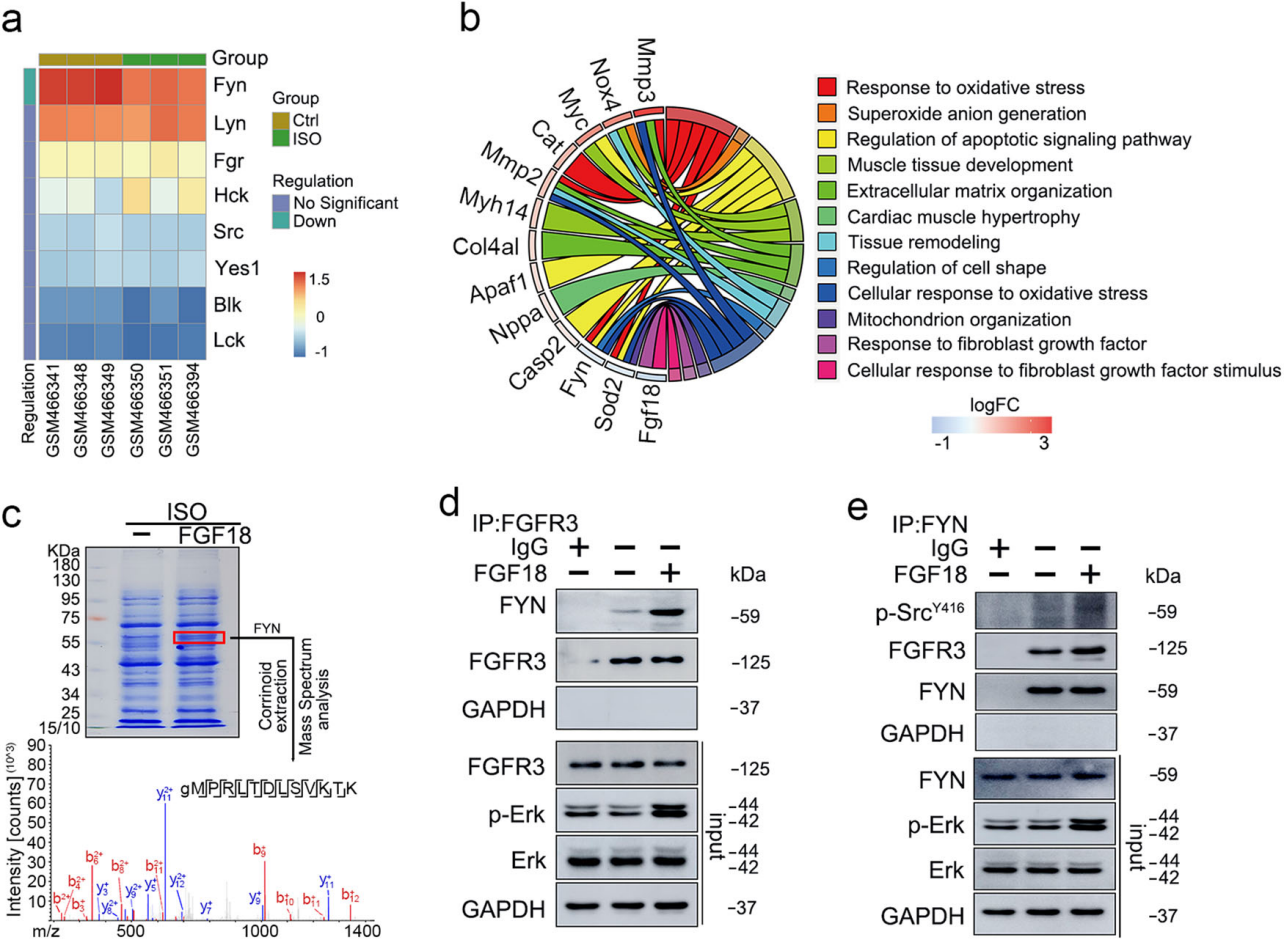
FYN expression is downregulated by hypertrophic stimuli and rescued by FGF18/FGFR3. **a** The corresponding Src family kinases correlated with the non-hypertrophic myocardium and hypertrophic myocardium were visualized by the heatmap in the normal and ISO treatment samples in GSE18801 dataset. The darker shade of red or blue represents the higher correlation level. **b** Circle plot depicting the important signal pathways associated with related genes. **c** NRCMs were treated with ISO (10 μM) in the presence or absence of FGF18 (50 ng/ml) for 48 h. Protein bands at ~60 kDa were excised from the SDS-PAGE gels for LC-MS/MS analysis. **d** NRCMs were treated with FGF18 (50 ng/ml) for 10 min. The cell lysates were subjected to immunoprecipitation with FGFR3 antibody, followed by immunoblotting with the FYN antibody. Cell lysates were also subjected to immunoprecipitation with IgG as negative control. *n* = 4. **e** NRCMs were treatment with FGF18 (50 ng/ml) for 10 min. The cell lysates were subjected to immunoprecipitation with FYN antibody, followed by immunoblotting with the p-SrcY416 and FGFR3 antibodies. Cell lysates were also subjected to immunoprecipitation with IgG as a negative control. *n* = 4. All numbers (*n*) are biologically independent experiments. Source data are provided as a Source data file.

FYN, a member of the Src family of non-receptor tyrosine kinases, is involved in cytoplasmic signal transduction cascades initiated by various membrane receptors^41^, and mediates the cellular responses to ROS and cardiac hypertrophy^18^. Analysis of the GSE18801 dataset (hypertrophic stimulation) showed that FYN was significantly downregulated in response to ISO treatment compared with other members of the Src family (Fig. 5a). KEGG pathway analysis showed that FYN and NOX4 were enriched in association with response to oxidative stress- and tissue remodeling-related signaling, whereas FGF18 was downregulated (Fig. 5b).

Given that FGF18/FGFR3 triggers downstream pathways and Src family kinases are the most frequently identified proteins in mass spectrometry analyses of the FGFR3 interactome^42^, we hypothesized that FYN might be involved in the cardioprotective effect of FGF18. To obtain this, we performed coomassie brilliant blue and liquid chromatography-tandem mass spectrometry (LC-MS/MS) analyses in the presence or absence of FGF18 after ISO treatment. Indeed, the band with a size of about 60 kDa was increased after FGF18 treatment in ISO-induced hypertrophic NRCMs, and FYN was detected by LC-MS/MS analyses (Fig. 5c; Supplementary Fig. 21). In addition, the results of immunoprecipitation assays showed that FGF18 promoted the association between FGFR3 and FYN, and induced tyrosine phosphorylation of FYN^Y416^ (Fig. 5d, e), indicating that FGF18/FGFR3 activated FYN by promoting its phosphorylation at Y416^18^. In addition, FGF18 overexpression promote the phosphorylation and expression of FYN in cardiomyocytes following ISO stimulation (Supplementary Fig. 10a, b). Therefore, the cardioprotection of FGF18 could be plausible attribute to the interaction between FGFR3 and FYN that enabled FGF18 to phosphorylate FYN and then activate its activity.

To further examine the role of FYN in the protective effects of FGF18, FYN was knocked down using adenovirus-mediated RNA interference in NRCMs followed by ISO treatment. FYN knockdown abolished the cardioprotective effects of FGF18, as evidenced by an increase in cardiomyocyte size (Supplementary Fig. 10c), higher level of *ANP*, *BNP*, *collagen I,* and *collagen III* (Supplementary Fig. 10d), and activation of MAPKs (Supplementary Fig. 10j). Meanwhile, FYN knockdown also promoted the accumulation of oxidation products (3-NT), ROS and hydrogen peroxide in cardiomyocytes (Supplementary Fig. 10g–i). The results of TUNEL assays (Supplementary Fig. 10e) and the Bax/Bcl-2 ratio (Supplementary Fig. 10j) indicated that FYN deletion block the anti-apoptotic effect of FGF18 under ISO treatment. Furthermore, no feedback effects were observed by silencing of FYN in NRCMs (e.g., FGF18 and p-FGFR3, Supplementary Fig. 11). Overall, these results suggest that FYN plays a critical role in protecting against cardiac hypertrophy as downstream of FGF18.

**Fig. 6:**
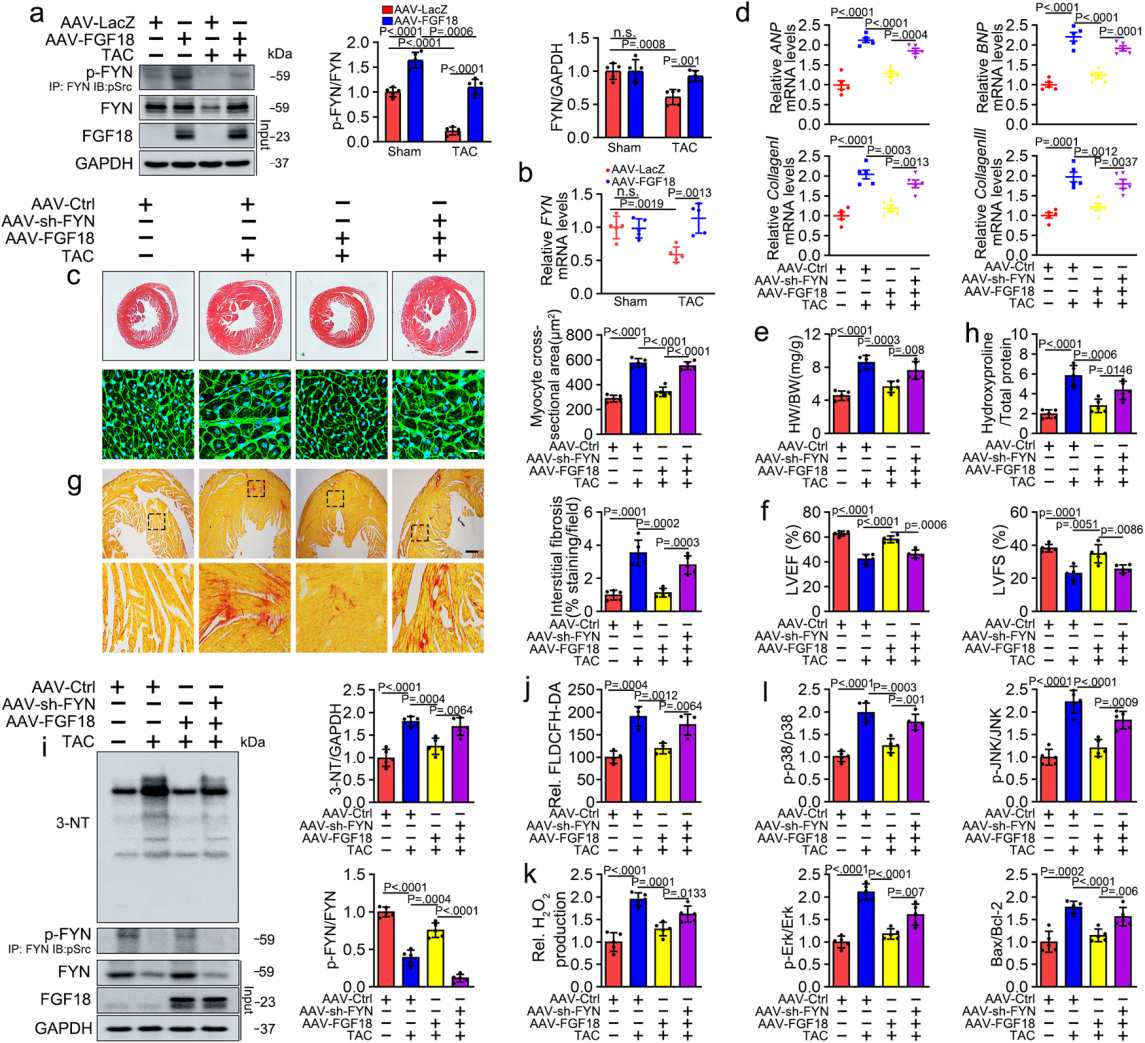
Loss of FYN abolishes the cardioprotective effects of FGF18 in vivo. **a**, **b** The mice were injected with AAV9-LacZ or AAV9-cTnT-FGF18 intravenously before Sham or TAC operation. **a** Western blotting with p-FYN (IP: FYN, IB: p-Src) and FYN antibody. Quantification of relative FYN activity and protein expression levels (right panel). *n* = 5. Data represent means ± SD. one-way ANOVA with Tukey multiple comparisons test. **b** qRT-PCR analysis of FYN levels. *n* = 5. Data represent means ± SD. one-way ANOVA with Tukey multiple comparisons test: n.s. = not significant. **c**–**i** AAV9-cTnT-FGF18 and control vector were injected intravenously into tail veins of 6 weeks old male C57BL/6J mice, respectively. One week after the injection, these mice were intravenously injected with the adeno-associated virus (AAV9-cTnT-sh-FYN; AAV9-Scramble) into tail veins. One week after the injection, these mice were subjected to Sham or TAC operation for 6 weeks. **c** Histological analyses of the HE staining and WGA staining (*n* = 5. Scale bar = 0.6 mm for upper HE staining; scale bar = 20 μm for lower WGA staining). Statistical results for the sectional cell area (right panel). *n* = 5. **d** Real-time quantitative PCR assays. *n* = 5. **e** Statistical results for the ratios of HW/BW in the indicated groups. *n* = 5. **f** Echocardiographic data for LVEF and LVFS are shown. *n* = 5. **g** PSR staining and Quantification (right panel). *n* = 5. Scale bar = 450 μm, and then zoom in 5 times. **h** Left ventricular collagen quantification by hydroxyproline assay (μg/mg). *n* = 5. **i** Levels of the oxidative damage marker 3-NT. The quantitative analysis of protein immunoblots (right panel), *n* = 5. **j** Total ROS levels (by DCFH-DA probe) were quantified. *n* = 4. **k** Hydrogen peroxide levels (by Amplex Red assay) quantified in different groups. *n* = 5. One-way ANOVA was followed by a post hoc Fisher’s comparison test. **l** Quantification of relative protein levels. *n* = 5. **c**–**i** All quantitative data are reported as means ± SD, one-way ANOVA with Tukey multiple comparisons test. All numbers (*n*) are biologically independent animals. Source data are provided as a Source data file.

Consistent with the results of in vitro studies, FGF18 overexpression restored the activity and expression of FYN in the PO-induced hypertrophic heart (Fig. 6a, b). To confirm the role of FYN, we used AAV9 vectors carrying shRNA (AAV9-cTNT-sh-FYN) to silence FYN in cardiomyocytes specifically. FYN-deficient mice (Supplementary Fig. 12; Supplementary Table 6) or FGF18 overexpressing mice (Fig. 2) shown no cardiac phenotypic differences at the basal level. In contrast, FGF18 overexpression decreased cardiomyocyte hypertrophy (Fig. 6c, e; Supplementary Figs. 13a and 14a, b), the mRNA levels of phenotypic marker (Fig. 6d; Supplementary Fig. 14c), cardiac dilation and contractile function (Fig. 6f; Supplementary Figs. 13b and 14d; Supplementary Tables 7, 8), myocardial fibrosis (Fig. 6g; Supplementary Fig. 14e), hydroxyproline content (Fig. 6h; Supplementary Fig. 14f), cardiomyocyte apoptosis (Supplementary Fig. 13c), 3-NT accumulation (Fig. 6i), ROS production (detected by DCFH-DA, Fig. 6j; Supplementary Fig. 14g), hydrogen peroxide levels (Fig. 6k; Supplementary Fig. 14h), and the activation of MAPKs (Fig. 6l; Supplementary Fig. 13d). However, these protective effects of FGF18 were inhibited in FYN-deficient mice (Fig. 6; Supplementary Figs. 13 and 14). Collectively, these data indicate that FYN is involved in the protective effects of FGF18 against cardiac hypertrophy in vivo.

### Cardiac FYN overexpression partially rescues PO-induced cardiac hypertrophy in Fgf18-CKO mice

**Fig. 7:**
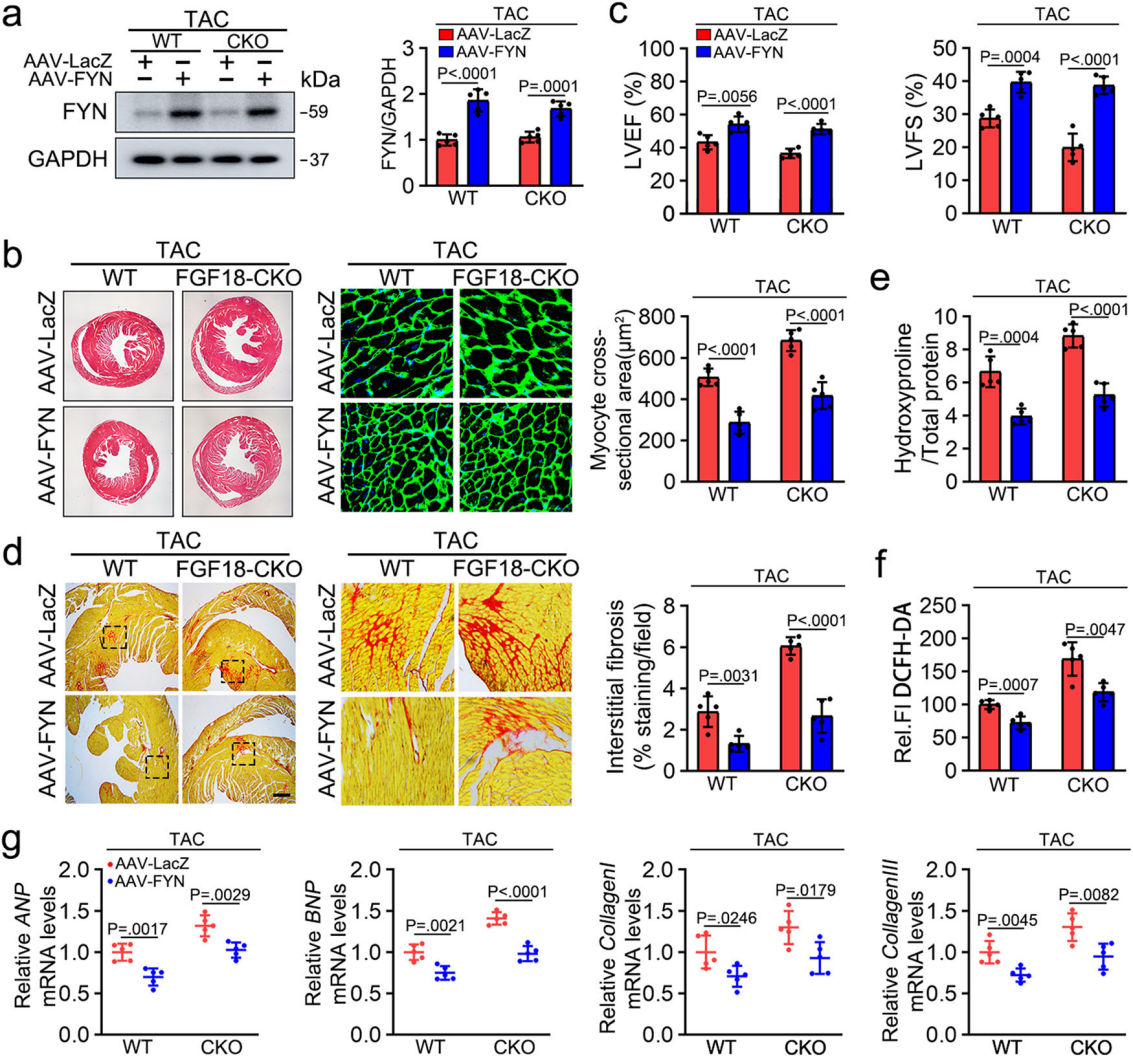
Cardiac FYN overexpression partially rescues PO-induced cardiac hypertrophy in *Fgf18-CKO* mice. *Fgf18*^f/f^ and *Fgf18*-*CKO* mice (αMHC-MerCreMer; *Fgf18*^f/f^) were intraperitoneally injected with tamoxifen at 6 weeks old male mice. FYN overexpression adeno-associated virus (AAV9-cTnT-FYN) and control vector (AAV9-LacZ) were injected intravenously into tail veins of 6 weeks old male mice. Two weeks after the injection, these mice were subjected to TAC operation. **a** Validation of overexpression of FYN by western blot. Quantification of relative protein levels (right panel). *n* = 5. **b** Histological analysis of the HE staining and WGA staining (*n* = 5. Scale bar = 0.6 mm for left HE staining; scale bar = 20 μm for right WGA staining). Statistical results for the sectional cell area (right panel). *n* = 5. **c** Representative echocardiographic data for LVEF and LVFS are shown. *n* = 5. **d** PSR staining and quantification (right panel). *n* = 5. Scale bar = 450 μm, and then zoom in 5 times. **e** Left ventricular collagen quantification by hydroxyproline assay (μg/mg). *n* = 5. **f** Total ROS levels (by DCFH-DA probe) were quantified. *n* = 5. **g** Real-time quantitative PCR assays. *n* = 5. All quantitative data are reported as means ± SD, one-way ANOVA with Tukey multiple comparisons test. All numbers (*n*) are biologically independent animals. Source data are provided as a Source data file.

To test our hypothesis that FYN plays a protective role in hypertrophic cardiomyopathy in *Fgf18-CKO* mice, we used AAV9 vectors carrying FYN under the control of the murine cTNT core promoter (AAV9-cTNT-FYN; Fig. 7a). FYN overexpression partially protected *Fgf18-CKO* mice from hypertrophy (Fig. 7b), as well as cardiac function (Fig. 7c, Supplementary Table 9), myocardial fibrosis (Fig. 7d), and hydroxyproline content (Fig. 7e). In addition, AAV9-FYN transfected *Fgf18-CKO* mice showed reduced ROS accumulation (detected by DCFH-DA, Fig. 7f) and decreased cardiac-specific expression of genes associated with hypertrophy and fibrosis (Fig. 7g) compared with those in WT mice after TAC. These findings confirm that FYN is involved in the protective effects of FGF18 against cardiac hypertrophy.

### FGF18 attenuates cardiomyocyte hypertrophy by promoting the interaction between FYN and NOX4

FGF18 suppressed ROS accumulation by increasing the activity and expression of FYN under conditions of myocardial hypertrophy, suggesting that the protective effect of FGF18-FYN on cardiomyocytes is related to the suppression of ROS generation. NOX4 is considered a key mediator of ROS production^9,12^, and FYN negatively regulates NOX4-induced ROS production by phosphorylating NOX4^18^. Furthermore, the GSE18801 dataset showed that NOX4 was upregulated in response to hypertrophic stimulation, accompanied by a decrease in FGF18 and FYN. These led us to hypothesize that FGF18-FYN restores redox homeostasis by suppressing NOX4. To test this hypothesis, we performed co-immunoprecipitation assays, which showed that ISO decreased the interaction between FYN and NOX4, and this effect was abolished by FGF18 overexpression (Supplementary Fig. 15a, b). Similar observations were made in mouse heart tissues. Considering that the FYN-NOX4 interaction was not altered at the basal level, but rather in response to TAC, namely, the TAC-decreased interaction was restored by FGF18 overexpression (Supplementary Fig. 15c, d). Therefore, the anti-hypertrophic effect of FGF18 might be mediated by the modulation of the FYN-NOX4 interaction.

**Fig. 8:**
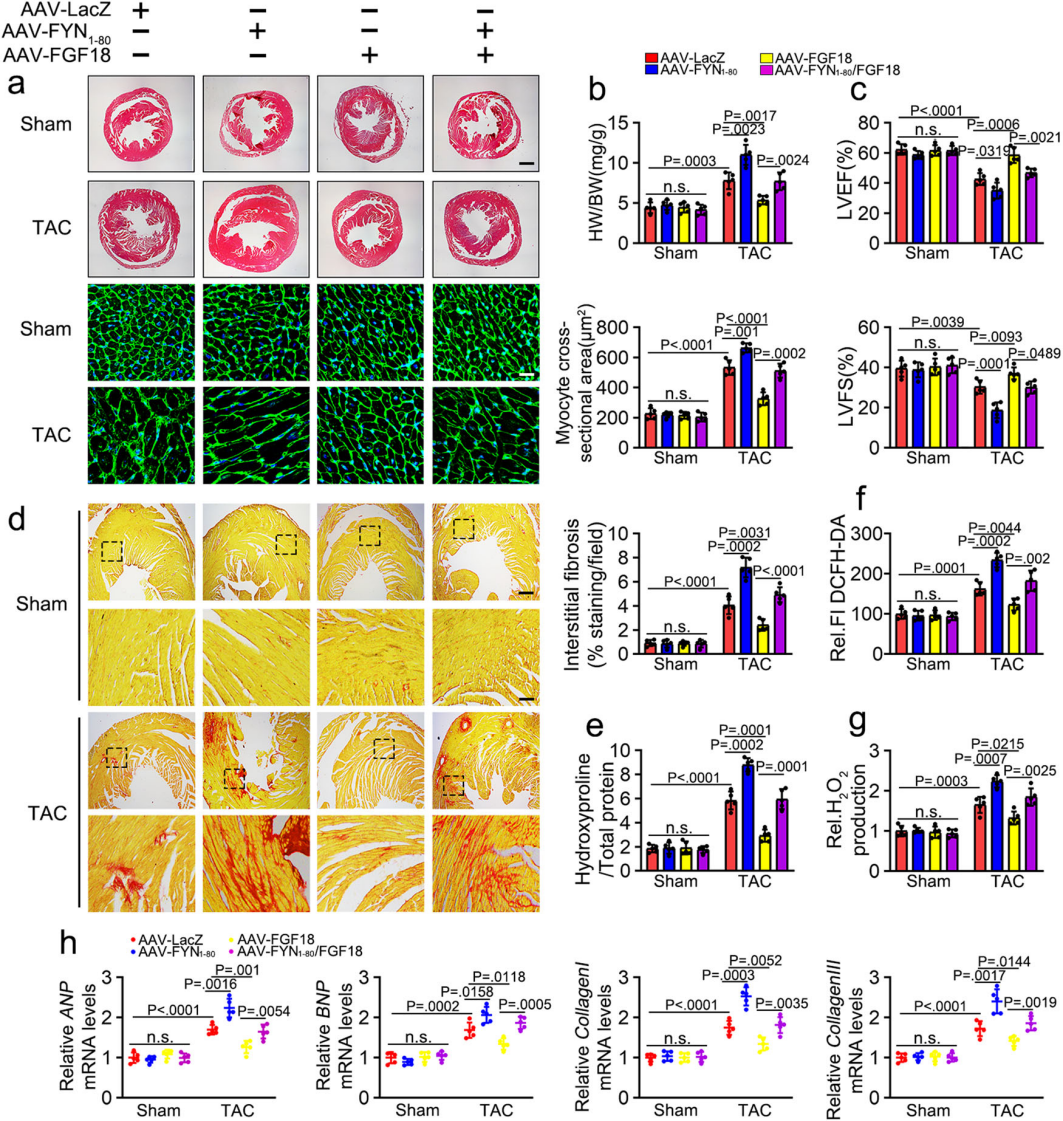
FYN_1-80_ aggravates pressure overload-induced hypertrophy. FGF18 overexpression adeno-associated virus (AAV9-cTnT-FGF18) and control vector (AAV9-LacZ) were injected intravenously into tail veins of 6 weeks old male C57BL/6J mice, respectively. One week after the injection, these mice were intravenously injected with the adeno-associated virus (AAV9-cTnT-FYN_1-80_; AAV9-LacZ) into tail veins. One week after the injection, these mice were subjected to Sham or TAC operation. **a** Histological analysis of the HE staining and WGA staining (*n* = 5. Scale bar = 0.6 mm for upper HE staining; scale bar = 20 μm for lower WGA staining). Statistical results for the sectional cell area (right panel). *n* = 5. **b** Statistical results for the ratios of HW/BW in the indicated groups. *n* = 5. **c** Echocardiographic measurement of LVEF and LVFS are shown. *n* = 5. **d** PSR staining and quantification (right panel). *n* = 5. Scale bar = 450 μm, and then zoom in 5 times. **e** Left ventricular collagen quantification by hydroxyproline assay (μg/mg). *n* = 5. **f** Total ROS levels (by DCFH-DA probe) were quantified. *n* = 5. **g** Hydrogen peroxide levels (by Amplex Red assay) quantified in different groups. *n* = 5. One-way ANOVA was followed by a post hoc Fisher’s comparison test. **h** Real-time quantitative PCR assays. *n* = 5. All quantitative data are reported as means ± SD, one-way ANOVA with Tukey multiple comparisons test: n.s. = not significant. All numbers (*n*) are biologically independent animals. Source data are provided as a Source data file.

To determine whether the direct interaction mediates the protective effect of FGF18 on the myocardium between FYN and NOX4, we constructed an adenovirus vector for FYN_1-80_ that retained the binding activity but lost the catalytic activity^18^. FYN_1-80_ transfection competitively reduced FGF18 promoted interaction between FYN and NOX4 (Supplementary Fig. 15e), and suppressed the anti-hypertrophic effects of FGF18 upon ISO stimulation, as demonstrated by increased cardiomyocyte surface area (Supplementary Fig. 16a) and upregulation of *ANP*, *BNP*, *collagen I* and *collagen III* (Supplementary Fig. 16b). The FYN_1-80_ group showed increased ROS production and TUNEL-positive cells under the above conditions (Supplementary Fig. 16c–e). To further investigate the functional effect of this interaction following FGF18 overexpression in vivo, the AAV9-cTNT-FGF18 and AAV9-cTNT-FYN_1-80_ vectors were introduced into the hearts of mice via tail vein injection, followed by Sham or TAC operation. Competitive inhibition of the FYN-NOX4 interaction by FYN_1-80_ blocked the protective effect of FGF18 (Supplementary Fig. 15f), as demonstrated by heart size changes (Fig. 8a, b; Supplementary Fig. 17a), and cardiac dysfunction (Fig. 8c; Supplementary Fig. 17b; Supplementary Table 10). In addition, the protective effects of FGF18 overexpression against TAC-induced fibrosis (Fig. 8d), hydroxyproline content (Fig. 8e), ROS (detected by DCFH-DA, Fig. 8f), hydrogen peroxide (Fig. 8g) and level of phenotypic marker genes (Fig. 8h) were blunted in the FYN_1-80_ group. Collectively, these data suggest that the interaction between FYN and NOX4 is essential for regulating FGF18 against myocardial hypertrophy.

### FGF18 prevents cardiac hypertrophy by decreasing NOX4 activity

Next, we investigated whether the anti-hypertrophy effects of FGF18 are mediated by the modulation of NOX4 activity. p22^*phox*^ is a pivotal component of NOX oxidases required for NOX4 functional activity^43^. Immunoprecipitation analysis showed that FGF18 negatively regulated the interaction between p22^*phox*^ and NOX4, suggesting that FGF18 maintains intracellular redox homeostasis by suppressing the activation of NOX4 (Supplementary Fig. 18a). To confirm this, NRCMs were treated with GKT137831, a NOX4 activity antagonist. GKT137831 has anti-hypertrophic effects similar to those of FGF18. GKT137831 significantly reduced cardiomyocyte surface area (Supplementary Fig. 18b) and downregulated *ANP*, *BNP*, *collagen I*, and *collagen III* (Supplementary Fig. 18c). GKT137831 also decreased the proportion of TUNEL-positive cells (Supplementary Fig. 18d), and 3-NT accumulation, ROS production (detected by DCFH-DA) and hydrogen peroxide (Supplementary Fig. 18e–g) in ISO-induced NRCMs hypertrophy. Meanwhile, GKT137831 restored the anti-hypertrophic effect of FGF18 in FYN-deficient cardiomyocytes, as indicated by the decreased cell surface area and ROS production and apoptosis (Supplementary Fig. 18b–g). Furthermore, NOX4 transfection abolished the cardioprotective effects of FGF18. Immunoprecipitation analysis revealed that NOX4 overexpression negatively regulated the cardioprotective effects of FGF18 by increasing the interaction between p22^*phox*^ and NOX4 (Supplementary Fig. 19a). Meanwhile, NOX4 overexpression increased the cardiomyocyte size (Supplementary Fig. 19b), apoptosis (Supplementary Fig. 19c), and the mRNA level of phenotypic markers (Supplementary Fig. 19d). Notably, FGF18 markedly reduced the accumulation of ROS (detected by DCFH-DA) and hydrogen peroxide, and these effects were abrogated by NOX4 overexpression (Supplementary Fig. 19e, f). Taken together, these results suggested that FGF18 upregulates FYN activity, which in turn negatively regulates NOX4 activity and then prevents cardiac hypertrophy.

## Discussion

Considerable advances have been made in understanding the complex mechanism that maintains myocardial homeostasis. In recent years, the potential role of FGFs in cardiovascular diseases has garnered considerable interest. The paracrine factors FGF3, FGF8, FGF9, FGF10, FGF15/19, and FGF16 are necessary for cardiac development^44^. Furthermore, FGF2^45^, FGF5^35^, FGF9^46^, FGF10^47^, FGF16^37^, and FGF21^48^ exert cardioprotective effects on the myocardium under pathological conditions, whereas FGF12^36^, FGF13^49^, and FGF23^50^ are involved in adaptive hypertrophic signaling and induce cardiac hypertrophy. Previously, we revealed that FGF21 alleviates Ang II-induced hypertrophy in a Sirt1-dependent manner^51^. Moreover, elevated FGF13 expression is associated with PO-induced hypertrophy, whereas deletion of FGF13 alleviates myocardial hypertrophy and cardiac function^52^. These studies indicate that FGFs play essential roles in the pathogenesis of cardiovascular diseases and probably serve as biomarkers of cardiac injury. Characterization of these biomarkers might not only reveal new signaling mechanisms but also lead to their investigation as new therapeutic targets that can help to maintain cardiac homeostasis after injury.

The paracrine factor FGF18, which selectively binds to FGFR3, has been extensively studied for its role in the bone and lung^21–26^. The role of FGF18 in the kidney^53^, liver^54^, endothelium^55^, and skeletal muscle^56^ in embryos and adults has also been studied to varying degrees. However, few studies have examined the relationship between FGF18 and cardiovascular diseases, and only the protective effect of FGF18 on cerebral ischemia^30^ and the increase in FGF18 levels in peripheral blood after myocardial infarction^31^ have been reported. The biological functions of FGF18 in the myocardium and the underlying mechanisms remain unclear and need to be extensively studied. Accordingly, in this study, we found that FGF18 expression in mice’s lungs, liver, and kidney was high at 4 weeks postnatally but was significantly decreased and lower than that in the heart at 14 weeks. Furthermore, analysis of the GSE18801 dataset showed that FGF18 was downregulated in hypertrophic hearts associated with increased ROS generation. These findings are in agreement with the observations in mice after hypertrophic stimulation. Thereby, we speculated that FGF18 might be critical for myocardial homeostasis.

Consistent with the Human Protein Atlas database (https://www.proteinatlas.org), we found that expression of FGF18 was higher in primary cardiomyocytes than in all other cell types. Under hypertrophic pressure stimulation, FGF18 changed significantly in cardiomyocytes but not in noncardiomyocytes, suggesting that FGF18 is mainly derived from cardiomyocytes and regulated by pressure stimulation. Therefore, the role of FGF18 in cardiomyocytes was explored. As a paracrine factor, FGF18 might be responsible for regulating crosstalk between cardiomyocytes and other cells, and further work is necessary to modulate cell-to-cell crosstalk. To systematically study the effect of FGF18 on the heart, *Fgf*18^+/−^*KO* and *Fgf18-CKO* mice were generated. The morphologies of organs that highly express FGF18, such as the kidney, lung, brain, and liver, were not significantly affected in these mice. However, knockdown of FGF18 (*Fgf18*^+/−^*KO* and *Fgf18-CKO*) aggravated ROS production, myocardial hypertrophy and functional injury after pressure overload in vivo and in vitro. By contrast, cardiomyocyte-specific FGF18 overexpression attenuated pathologic cardiac hypertrophy associated with decreased ROS accumulation. Knockdown of FGFR3 severely blunted the protective effect of FGF18 in NRCMs after ISO treatment. These results indicate that FGF18/FGFR3 elicit a cardioprotective effect by negatively regulating ROS generation. In addition, recent studies showed that intra-articular injection of FGF18 ameliorates oxidative stress in an osteoarthritis model, which supports the potential regulatory function of FGF18 upon oxidative stress^26–29^.

The balance between ROS generation and the action of antioxidants under physiological conditions is crucial for redox homeostasis. Clinical and experimental studies indicate that oxidation is involved in developing cardiac hypertrophy and heart failure^10,57^. However, reversing the ROS-scavenging deficit in hypertrophic cardiomyocytes by exposure to antioxidants, such as N-acetylcysteine, oxypurinol, and vitamins E and C, has failed to reverse the hypertrophic phenotype and improve cardiovascular outcomes in clinical trials^58–60^. Therefore, inhibition of ROS production is considered a complementary strategy to prevent cardiac hypertrophy^10^.

FYN, a member of the Src family of non-receptor tyrosine kinases, plays multifactorial roles in cardiac homeostasis under different pathophysiological conditions. For example, FYN can be activated by oxidative stress and, in turn serves as a negative feedback regulator of NOX4 in cardiomyocytes during cardiac remodeling^18^. Therefore, FYN is considered a potential target for myocardial protection, and activation of FYN provides autonomic feedback protection in the early stages of injury, with its phosphorylation at position 416 leading to downregulation of NOX4 activity. However, under chronic stress, the function of FYN gradually weakens, and it is challenging to maintain myocardial homeostasis in mice^18^, indicating that autonomic feedback of FYN is insufficient to resist continuous pressure on the myocardium. Therefore, it is particularly important to identify factors that can positively regulate FYN and restore its activity and function upon sustained myocardial injury. In this study, the GSE18801 dataset analysis showed that FYN was significantly downregulated in response to ISO treatment compared with other members of the Src family, accompanied by the decrease of FGF18. Given that FGF18/FGFR3 activate downstream pathways and Src kinases^61,62^, which are the most frequently identified proteins in mass spectrometry analysis of the FGFR3 interactome^42^. Also, LC-MS/MS analyses demonstrated that FYN might be involved in the cardioprotective effect of FGF18. We therefore hypothesized that FYN might be involved in the cardioprotective effect of FGF18. Consistently, we showed that FGF18 promoted the interaction between FGFR3 and FYN and induced phosphorylation of FYN^Y416^ in NRCMs. Phosphorylation of FYN^Y416^ increases its activity and is considered an important negative trigger for its downstream substrates^18^. A further experiment demonstrated that FYN levels were lower in mice hypertrophic hearts than in normal hearts, and FGF18 overexpression markedly increased the expression and activity of FYN in vivo and in vitro. By contrast, deletion of FYN abrogated the protective effects of FGF18, including its antioxidant, antiapoptotic, and antihypertrophic effects, suggesting that FYN might be the critical factor involved in FGF18-mediated protection of cardiomyocytes. Interestingly, AAV-mediated cardiac overexpression of FYN did not wholly rescue PO-induced cardiac hypertrophy in *Fgf18-CKO* mice, indicating that the full biological function of FYN is attributable to FGF18-regulated FYN quantification and activity. In addition, FGF18-induced FYN expression increased the possibility of FYN phosphorylation and substrate binding. FYN expression has been mainly reported to be regulated by noncoding RNAs. For example, microRNA-125a-3p negatively regulates FYN expression^63^. However, FGF18 as a paracrine factor, influences FYN expression would be indirect and complicated. Further elucidation of how FYN expression is regulated during hypertrophic stimulation will provide new insights into the mechanism by which FGF18 ameliorates hypertrophy. Together, these results revealed that FYN is positively regulated by FGF18, which is an important factor that protects cardiomyocytes upon chronic myocardial injury.

The role of NOXs, which are the predominant sources of ROS in cardiovascular diseases, has been reviewed in detail^64–67^. Specifically, NOX2 induces oxidative stress-related damage in response to ischemia/reperfusion, whereas it is not essential for the development of cardiac hypertrophy in a PO model^68^. NOX4, a significant source of ROS in the heart, localizes to intracellular membranes in mitochondria, the endoplasmic reticulum, and the nucleus, and derived ROS contribute to cardiomyocyte apoptosis, cardiac fibrosis, and hypertrophy^16,17,57,69^. Therefore, NOX4 is a target for the development of novel therapeutic strategies for cardiovascular diseases^70^. As known, the activity of NOX4 is primarily regulated via modulation of its expression level and that of its membrane partner p22^*phox*^^71–73^. In addition, phosphorylation of NOX4^Y566^ by FYN is the key mechanism that reduces the association of p22^*phox*^ and NOX4 and optimizes ROS production in cardiomyocytes^18^. In this study, analysis of the GSE18801 dataset showed that NOX4 was significantly upregulated, and oxidative stress was significantly increased under hypertrophic pressure, while FYN and FGF18 were downregulated. In addition, overexpression of FGF18 significantly increased the activity of FYN and decreased the association of NOX4 and p22^*phox*^, accompanied by a decrease in ROS. Consistently, GKT137831, an inhibitor of NOX4 activity, attenuated ISO-induced oxidative stress and cardiomyocyte hypertrophy. Meanwhile, GKT137831 restored the anti-hypertrophic effect of FGF18 in FYN-deficient cardiomyocytes. These results demonstrated that the FGF18/FGFR3-mediated beneficial effect of FYN on oxidative stress is related to the suppression of NOX4 activity. Since the unique domain of FYN_1-80_ retains its binding activity but lost its biological function, and mediates its binding to NOX4 in cardiomyocytes^18^, we further explored the role of the FYN-NOX4 interaction in the protective effect of FGF18 by generating FYN_1-80_ recombinant protein. As expected, FYN_1-80_ abolished the cardioprotective effect of FGF18 by interfering with the interaction between endogenous FYN and NOX4, supporting the notion that FGF18 elicits antioxidant effects by modulating the activity of FYN.

**Fig. 9:**
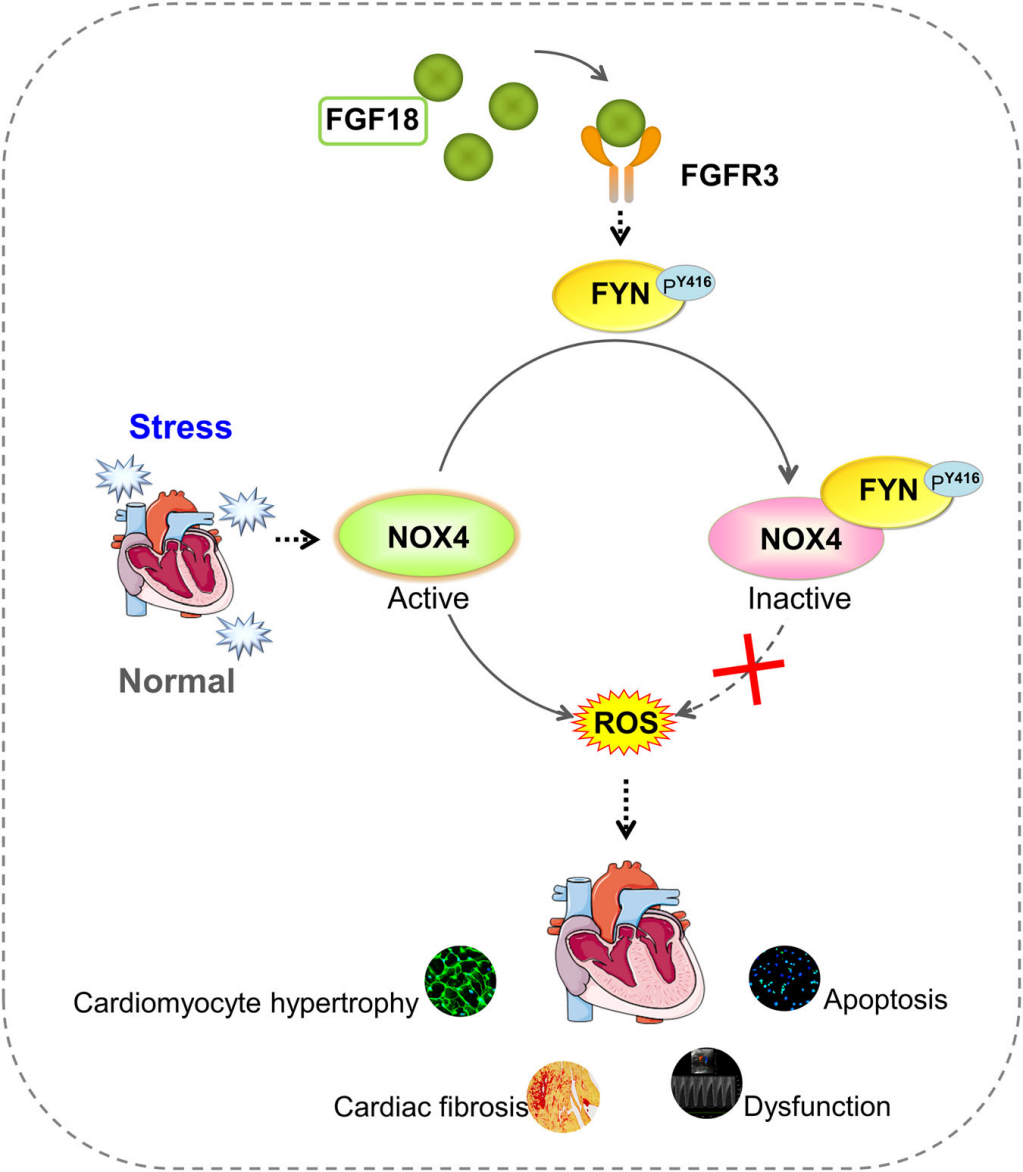
Schematic figure illustrates that FGF18 ameliorates pathological cardiac hypertrophy and dysfunction by maintaining redox homeostasis by FYN/NOX4 signal axis. FGF18/FGFR3 promoted the activity and expression of FYN and negatively regulated NOX4 activity, thereby inhibiting ROS generation and protecting heart function by inhibiting cardiac fibrosis and hypertrophy. In conclusion, the present study identifies a previously unknown biological function of FGF18 in pathological cardiac hypertrophy. Part of the figure is modified from Servier Medical Art (http://smart.servier.com/), licensed under a Creative Common Attribution 3.0 unported License.

In conclusion, the present study identified a previously unrecognized biological function of FGF18 in cardiac hypertrophy. FGF18/FGFR3 increase the activity of FYN, which negatively regulates NOX4 activity and prevents oxidative stress, apoptosis, and cardiac remodeling (Fig. 9). This study provides insight into the molecular mechanisms underlying the cardioprotective effect of FGF18 against pathological hypertrophy and suggests a potential therapeutic approach to prevent detrimental outcomes.

## Methods

### Animal procedures

All animal experiments and methods performed in this study followed ethical guidelines for animal studies and were approved by the Institutional Animal Care and Use Committee of Wenzhou Medical University, China.

Adult (6-week-old) male C57BL/6J mice were obtained from the Model Animal Research Center of Nanjing University. *Fgf18* flox/flox (*Fgf18*^f/f^) mice on C57BL/6J background were gifted by professor Shen of Wenzhou University (Supplementary Fig. 20b). Mice with tamoxifen-inducible Cre-fusion protein under the control of the cardiomyocyte-specific α-myosin heavy-chain promoter (αMHC-MerCreMer) were previously described^74^. αMHC-MerCreMer transgenic mice were crossed to *Fgf18*^f/f^ mice to deplete FGF18 expression in adult cardiomyocytes under tamoxifen administration. For wild-type (WT, *Fgf18*^f/f^) mice and *Fgf18-CKO* (*Fgf18*^f/f^ crossed with αMHC-MerCreMer) mice, tamoxifen (Santa Cruz Biotechnology, sc-208414) was administered at the dose of 35 mg/kg/day for 5 consecutive days by intraperitoneal (i.p.) injection. After the injection, all mice were kept for a 14-day waiting period to get the efficient gene knockout. Genotypes of the transgenic mice were detected by polymerase chain reaction (PCR) analysis using the DNA from the mouse tail, and the specific primers were used as follows:

αMHC-MerCreMer

Forward: 5’-GCGGTCTGGCAGTAAAAACTATC-3’

Reverse: 5’-GTGAAACAGCATTGCTGTCACTT-3’

*Fgf18* ^f/f^

Forward: 5’-CTGAATCAAGAGAAGGACACCAAG-3’

Reverse: 5’-TACTTTGAGGAGTAGAAGGCTCTG-3’

The FGF18 heterozygous (*Fgf18*^+/−^) mice on C57BL/6J background was a generous gift from professor Shen of Wenzhou University (Supplementary Fig. 20a). Breeding of transgenic mice follows Mendel’s laws, with equal male to female ratios. All mice were housed in an environmentally controlled room for 4–6 days to adapt to the environment before experimentation. All animals were kept in a standard laboratory condition of temperature 21 ± 2 °C, relative humidity 50 ± 15%, 12 h light-darkness cycles, with water and food available ad libitum. When mice reach an experimental endpoint, all mice were euthanized by cervical dislocation.

AAV9 harboring FYN shRNA (AAV9-cTnT-sh-FYN), FGFR3 shRNA (AAV9-cTnT-sh-FGFR3) and control vector (AAV9-cTnT-Scrambled); FYN overexpression vector (AAV9-cTnT-FYN); FYN_1-80_ overexpression vector (AAV9-cTnT-FYN_1-80_), FGF18 overexpression vector (AAV9-cTnT-FGF18) and control vector (AAV9-cTnT-LacZ) were injected intravenously into tail veins of 6 weeks old male C57BL/6J mice respectively. The 1 × 10^12^ vg of virus Sham or TAC operation was conducted 2 weeks after the AAV9 injection. After 6 more weeks, echocardiography was carried out, and tissue samples were collected for further analysis.

### Isolation and culture of neonatal rat cardiomyocytes (NRCMs)

NRCMs were isolated from the ventricles of Sprague-Dawley rats (2 weeks pregnant, obtained from the Model Animal Research Center of Nanjing University) on a postnatal day 2 using the techniques described previously^75,76^. Briefly, 1- to 2-day-old SD rat pups were rinsed quickly in 75% ethanol solution for surface sterilization. Hearts were extracted from the body with curved scissors and transferred immediately into cold DPBS (Gibco, 14190144). Tissue was minced into small pieces (approximately 0.5–1 mm^3^, or smaller), which were then transferred into a tube containing trypsin medium [0.08% trypsin (Solarbio, T8150), 0.8% NaCl (Sigma-Aldrich, V900058), 0.03% KCl (Sigma-Aldrich, V900068), 0.035% NaHCO_3_ (Sigma-Aldrich, V900182), 0.1% D-(+)-Glucose (Sigma-Aldrich, G7021), and 0.2% Hepes (Sigma-Aldrich, H3375)] with 37 °C water bath and magnetic stirring (100 rpm) for 8 min per time.

After enzymatic digestion, cardiomyocytes and fibroblasts were purified by Percoll density gradient centrifugation (Sigma-Aldrich, P4937). We made two-layer density gradients consisting of red-colored 65% Percoll solution underneath transparent 45% Percoll solution in 15 mL tubes. The cell suspension was layered on top of the gradient, and the tubes were centrifuged at 3000 rpm for 30 min. The fraction of cardiomyocytes was harvested from the newly formed layer between the Percoll solutions. Finally, cardiomyocytes were plated at a density of 5 × 10^5^ cells per well onto collagen (Sigma-Aldrich, C919) coated six-well culture plates, which consisted of DMEM/F-12 (Gibco, 11330032) supplemented with 10% fetal calf serum (Gibo, 16010159) and 1% penicillin/streptomycin for 24 h at 37 °C with 5% CO_2_.

As described previously, the NRCMs were then incubated with isoprenaline (ISO, 10 μM, Sigma-Aldrich, I5627) for 48 h to induce cardiomyocyte hypertrophy^77^. The induction of hypertrophy was confirmed by determining the cell volume (cardiac troponin-T staining). GKT137831 (20 μM, Selleck, 1218942-37-0), an inhibitor of NOX4, was used to inhibit the activity of NOX4 in NRCMs^78,79^.

For RNA interference, cells were transfected with FGFR3 siRNA (Origene, SR513507) or control scrambled siRNA (Origene, SR30004) by Lipofectamine 3000 for 12 h in Opti-MEM. After the transfection, cells were removed to a full-growth medium for another 12 h and then were analyzed for further studies.

Recombinant human FGF18 (produced by Wenzhou Medical University gene engineering laboratory) were co-cultured in NRCMs (50 ng/mL) to verify the effective duration of FGF18 at different time.

### Isolation and culture of adult mouse cardiomyocytes

Adult mouse cardiomyocytes from 8–12 weeks old C57BL/6J mice were isolated as previously described^71^ and were cultured in medium 199 (M199) supplemented with 10% FBS and 1% penicillin/streptomycin with 5% CO_2_ at 37 °C.

### Immunoblotting analysis

Briefly, the protein concentration was determined using Pierce BCA Protein Assay Reagent (Thermo Fisher Scientific, 23228). 30 μg protein from each sample was resolved by SDS-PAGE on Tris-glycine gels, and transferred to a polyvinylidene fluoride membrane. Membranes were blocked with 5% bovine serum albumin (BSA, Sigma-Aldrich, B2064) in Tris-buffered saline (Sigma-Aldrich, T5030) containing 0.1% Tween 20 (Sigma-Aldrich, 93773) (TBST) and incubated with primary antibodies overnight at 4 °C. Membranes were washed 3 times for 5 min with TBST, incubated with either HRP-goat anti-mouse (Abcam, ab6789, 1:5000) or HRP-goat anti-rabbit (Abcam, ab6721, 1:5000) secondary antibodies for 1 h at room temperature. Bound antibody was visualized using Pierce ECL plus western blotting substrate (Thermo Fisher Scientific, 32132). The protein bands were visualized by exposure machine (GE, Amersham 154 Imager 680) and quantified using Image Quant 5.2 software (Molecular Dynamics, Sunnyvale, CA). The primary antibodies were p-p38 (CST, 4511, 1:1000), p38 (CST, 8690, 1:2000), p-Erk (CST, 9101, 1:2000), Erk (CST, 9102, 1:2000), p-JNK (CST, 4668, 1:1000), JNK (Abcam, ab179461, 1:2000), Bax (CST, 2772, 1:2000), Bcl-2 (Santa cruz, sc-7382, 1:1000), FGF1 (Abcam, ab207321, 1:1000), FGF2 (Santa cruz, sc-74412, 1:1000), FGF3 (Santa cruz, sc-135, 1:1000), FGF5 (Santa cruz, sc-376264, 1:1000), FGF9 (Santa cruz, sc-373716, 1:1000), FGF13 (Affinity biosciences, DF4699, 1:1000), FGF16 (Santa cruz, sc-390547, 1:1000), FGF18 (Santa cruz, sc-393471, 1:1000), FGFR3 (Santa cruz, sc-390423, 1:1000), FYN (Santa cruz, sc-365913, 1:1000), p-Src (CST, 2101, 1:1000), 3-NT (Abcam, ab61392, 1:1000), NOX4 (Origene, TA349083, 1:1000), p22^*phox*^ (Santa cruz, sc-271968, 1:1000). The expression of GAPDH (glyceraldehyde-3-phosphate dehydrogenase) (Abcam, ab9485, 1:1000) was used as a loading control.

### Immunoprecipitation

NRCMs or heart tissues were lysed with IGEPAL CA-630 buffer (50 mM Tris-HCl, pH 7.4, [Sigma-Aldrich, T5030], 1% IGEPAL CA-630 [Sigma-Aldrich, I8896], 10 mM EDTA, 150 mM NaCl, 50 mM NaF, 1 μM leupeptin [Sigma-Aldrich, L5793], 0.1 μM aprotinin [Sigma-Aldrich, SRE0050]). Samples were incubated for 2 h with primary antibody after being precleared with 30 μL PureProteome™ Protein A/G Mix Magnetic Beads (Merck Millipore, LSKMAGAG10) at 4 °C. Cell lysates were also subjected to immunoprecipitation with either mouse IgG1 isotype control (Cell Signaling Technology, 5415) or rabbit IgG isotype control (Cell Signaling Technology, 3900) according to the immunoglobulin type of primary antibody. After immunoprecipitation, the samples were washed five times with TBS. They were then eluted with glycine-HCl (0.1 M, pH 3.5) and the immunoprecipitates were subjected to immunoblotting using specific primary antibodies.

### Transverse aortic constriction (TAC)

C57BL/6J male mice (body weight 21 ± 2 g) were anesthetized with isoflurane, intubated, and a transsternal thoracotomy performed. The transverse aorta was constricted by tying a 7–0 nylon suture ligature against a 28 gauge needle between the innominate artery and left carotid artery, which was promptly removed to yield a constriction of 0.35 mm in diameter. The sham operation was identical to those described above, except that the knot was not tied around the aorta. After the completion of the operation, the mice were transferred to a heating pad and closely monitored. Echocardiograph (VisualSonics 1100, Toronto, Ontario, Canada) was used to measure the right-to-left carotid artery flow velocity ratio after TAC, and the mice with a carotid artery flow velocity ratio >4000 mm/s were regarded as eligible models (Supplementary Fig. 7e).

### Echocardiography

Echocardiograms were obtained using a Vevo 1100 Ultrasound System (VisualSonics, Toronto, Canada) equipped with a high-frequency (30 MHz) linear array transducer. Echocardiography was performed 6 weeks following the TAC operation. The mice were anaesthetized with isoflurane (3% for induction and 1–1.5% for maintenance) mixed in 1 L/min O_2_ via a facemask. To minimize the confounding influence of different heart rates on the aortic pressure gradient and left ventricular function, the flow of isoflurane was adjusted to anesthetize the mice while maintaining their heart rates at 400–500 beats per minute. Hair was removed from the anterior chest region using a chemical hair remover. Body temperature was carefully maintained at close to 37.0 °C using a rectal temperature probe and heating blanket. Long-axis M-mode was used for the measurement of systolic and diastolic ventricular diameter and wall thickness. The left ventricular end-diastolic diameter (LVEDD), left ventricular end-systolic diameter (LVESD), left ventricular end-diastolic volume (LVEDV), and left ventricular end-systolic volume (LVESV) were measured as indicators of dilative remodeling. Left ventricular fractional shortening (LVFS = [LVEDD − LVESD] × 100/LVEDD) and left ventricular ejection fraction (LVEF = [LVEDV − LVESV] × 100/LVEDV) were calculated for assessment of systolic function. Systolic and diastolic anatomic parameters were obtained from M-mode tracings at the mid-papillary level. All views were digitally stored in cine loops consisting of >300 frames. Subsequent analysis was performed off-line on a workstation installed with Vevo LAB software (version 1.7.1) (VisualSonics, Toronto, Canada) by an experienced operator who was blind to the treatment allocation and was unaware of data from other modalities.

### Heart weight, body-weight assessment, and histological examination

After the completion of the experiments, the animals were euthanized. The hearts were harvested and arrested in diastole with 0.5% KCl (w/v) in PBS. The heart weights (HW) were determined and normalized to the body weight (BW). Then, part of each heart was immersed in 4% paraformaldehyde perfusion at 4 °C overnight. On the next day, the tissue samples were transferred to 70–100% ethanol for dehydration before embedding in paraffin. Heart sections (5 µm intervals) were deparaffinized and stained with hematoxylin and eosin (H&E), picrosirius red staining (Sigma-Aldrich) according to routine procedures. Images were acquired using Nikon Eclipse Ni light microscopy.

To determine the myocyte cross-sectional area, heart sections were incubated with wheat germ agglutinin-Alexa488 (WGA-Alexa488, Thermo Fisher Scientific, W11261) for 1 h to visualize the membranes and with 4,6 diamidino-2-phenylindole (DAPI) for 20 min to observe the nuclei. At least 100 randomly selected myocytes were measured from five animals/groups, and the average values were used for analysis. All measurements were made using ImagePro Plus software version 7.0 (Media Cybernetics, Rockville, MD). All morphometric analyses were performed in a blinded fashion. Sections were measured using a Leica TCS SP8 Confocal microscope (Leica, Wetzlar, Germany). All histopathologic data were confirmed by a blinded pathologist.

### Adenovirus constructs

Recombinant adenovirus vectors were constructed, propagated, and titered as previously described^80^. Briefly, pBHGloxΔE1, 3 Cre plasmid (Microbix, PD-01-40), including the ΔE1 adenoviral genome, was cotransfected with the pDC316 shuttle vector (Microbix, PD-01-28) containing the gene of interest into HEK293T cells (Procell Life Science & Technology, CL-0005) using Lipofectamine 2000 (Thermo Fisher Scientific, 11668019). Through homologous recombination, the test genes were integrated into the E1-deleted adenoviral genome. The viruses were propagated in HEK293T cells. We made replication-defective adenovirus type 5 (devoid of E1) harboring rat FYN_1-80_ (Ad-FYN_1-80_), NOX4 (Ad-NOX4), FGF18 (Ad-FGF18) and an in-house-generated Ad-*LacZ* were used as a control. For adenovirus-mediated gene transfer, these constructed adenovirus vectors were transfected into NRCMs at an MOI of 20×PFU/cell for 24 h. After 24 h, the overexpression efficiency was evaluated.

### Construction of shRNA adenoviral expression vectors

The pSilencer 2.1-U6 expression vector was purchased from Ambion (Ambion, AM5762). The RNU6-1 RNA polymerase III promoter and the polylinker region were subcloned into the adenoviral shuttle vector pDC311 (Microbix, PD-01-25). The rat FYN shRNA targeting sequence was 5ʹ-AGGATAAAGAAGCAGCGAAACTGAC-3ʹ. The rat FGF18 shRNA targeting sequence was 5ʹ-CCTGCACTTGCCTGTGTTT-3ʹ. For Scrambled shRNA, an in-house-generated shRNA adenovirus that encodes a scrambled sequence was used as a control. Recombinant adenoviruses were generated by homologous recombination in HEK293T cells as described above. For adenovirus-mediated gene knockdown, these constructed adenovirus vectors were transfected into NRCMs at an MOI of 20× PFU/cell for 24 h. After 24 h, the knockdown efficiency was evaluated.

### Adeno-associated virus production

We used AAV9 because it shows strong tropism for the heart, particularly for cardiomyocytes and yields long-term gene expression^81^. Recombinant virus was produced as described below. Briefly, murine FGFR3 and FYN shRNA (FYN shRNA targeting sequence was 5ʹ-CCTGTATGGAAGGTTCACAAT-3ʹ; FGFR3 shRNA targeting sequence was 5ʹ-AGCAGTTGGTAGAGGATTTAG-3ʹ) or a scrambled sequence was initially inserted into a rAAV plasmid consisting of the vector genome, a murine cardiac troponin T (cTnT) core promoter and AAV ITRs flanking the expression cassette. Meanwhile, we have constructed the rAAV to deliver the murine FGF18, FYN, and FYN_1-80_ cDNA under the control of the murine cTnT core promoter. An in-house-generated AAV-LacZ was used as a control. To generate the virus, the plasmid carrying the target gene cassette was cotransfected into HEK293T cells with a packaging plasmid and an adenovirus helper plasmid. The packaged, recombinant viral particles were then purified by a CsCl gradient sedimentation method, desalted by dialysis, and subjected to a quality control analysis as described^82^.

### Oxidative stress measurement

Myocardial superoxide generation was measured via in situ dihydroethidium (DHE, Thermo Fisher Scientific, D11347) staining. Briefly, NRCMs were incubated with DHE (5 μM) in a humidified chamber at 37 °C, followed by PBS washing. The staining (red) images were captured with the microscope (EVOS, Thermo Fisher Scientific, MA, USA). The fluorescence intensity of DHE staining was measured using the ImageJ software (version 1.8.0), and the results were expressed as fold change against the corresponding controls.

Amplex Red assay (Invitrogen) was used to detect hydrogen peroxide levels in mouse hearts and NRCMs according to the manufacturer’s protocol. For mouse hearts, fresh frozen tissue (30–60 μg) was incubated in Amplex Red working solution (containing 100 μM Amplex Red reagent and 1 U/mL horseradish peroxidase) in HEPES buffer for 30 min at 37 °C. The supernatant was then collected, and the fluorescence was measured at 530 nm excitation wavelength and 590 emission wavelength using a Mithras LB 940 reader (Berthold Technologies). All procedures were protected from light, and a standard curve using fresh H_2_O_2_ was generated for comparison.

For the detection of hydrogen peroxide levels in NRCMs cells. Equal amounts of cells seeded in 96-well plates were incubated with Amplex Red working solution (containing 50 μM Amplex Red reagent and 0.1 U/mL horseradish peroxidase) for 30 min at 37 °C. Fluorescence was measured at 530 nm excitation wavelength and 590 nm emission wavelength using a Mithras LB 940 reader (Berthold Technologies). All procedures were protected from light, and a standard curve using fresh H_2_O_2_ was generated for comparison.

The production of ROS was measured using the 2’,7’-dichlorodihydrofluorescein-diacetate (DCFH-DA, Sigma-Aldrich, D6883). For the in vivo study, 5 μg tissue homogenate was incubated with 4 μM DCFH-DA for 10 min at 37 °C, and for the in vitro study, equal amounts of cells cultured in 96-well plates were incubated with 10 μM DCFH-DA for 20 min at 37 °C, protected from light. The fluorescence was then measured at 485 nm excitation wavelength and 535 nm emission wavelength using a Mithras LB 940 reader, and the data were analyzed with MikroWin 2010 software (Berthold Technologies).

### Immunofluorescent staining

Briefly, the cardiomyocytes or deparaffinized heart sections (5 µm intervals) were subsequently fixed with 4% paraformaldehyde for 20 min and permeabilized with 0.5% (v/v) Triton X-100 in PBS for 20 min at 37 °C. After wash, the samples were blocked in 2% (w/v) BSA in PBS for 2 h and incubated with primary antibody at 4 °C overnight. In our studies, the following primary antibodies were used: troponin T-FITC (Abcam, ab196384, the cardiomyocytes marker) and WGA-Alexa488 (Thermo Fisher Scientific, W11261). After incubation and washing with PBS, the nuclei were stained with DAPI for 15 min. Images were captured with a confocal laser scanning microscope (Leica TCS SP8, Wetzlar, Germany). The total tissue positive areas were measured with ImageJ software (version 1.8.0). Data were expressed as a percentage of positive staining area to the total area.

### RNA isolation and quantitative real-time-PCR (qRT-PCR)

Total RNA was extracted from heart tissue and cardiomyocytes using TRIzol reagent (Takara Bro Inc, 9108), as described by the manufacturer’s instructions. The RNA samples (1 ng) were reversely transcribed to cDNA by the Hiscript ® III Reverse Transcriptase kit (Vazyme, R223-01). qRT-PCR analysis was performed on a QuantStudio™ 3 Real-Time PCR Detection System using ChamQ Universal SYBR qPCR Master Mix (Vazyme, Q711-02) with specific primers. The relative expression levels of each gene were quantitated using the 2^−∆∆CT^ method and normalized to the amount of endogenous Glyceraldehyde-3-phosphate dehydrogenase (GAPDH). The sequences of specific primers used for qRT-PCR in this study are listed in Supplementary Table 1.

### TUNEL staining

The cardiomyocytes or deparaffinized heart sections (5 µm intervals) were then stained with the DeadEnd™ Fluorometric TUNEL System (Promega, G3250) according to the manufacturer’s protocol. DAPI was used for nuclear staining. Images were visualized and captured with a confocal laser scanning microscope (Leica TCS SP8, Wetzlar, Germany). TUNEL-positive cardiomyocytes were counted in randomly selected three fields of the slide. One hundred cells per field were counted, and the percentage of TUNEL-positive cells was calculated.

### Hydroxyproline assay

Total collagen content was measured using a hydroxyproline assay kit (Solarbio, BC0255) according to the manufacturer’s protocol. Snap frozen left ventricular free wall were hydrolyzed in 6 M HCl at 120 °C for 16 h and neutralized with 6 M NaOH. From each sample, 5 µL of the final neutralized hydrolysate was used. Hydroxyproline concentration was measured using a microplate reader and normalized to the protein concentration.

### Sample preparation for LC-MS/MS analysis

Protein bands at ~60 kDa were excised from the SDS-PAGE gels, reduced with 10 mM of DTT and alkylated with 55 mM iodoacetamide, and then digested with Sequence Grade Modified Trypsin (Promega, Madison, WI) in ammonium bicarbonate buffer at 37 °C overnight. The digestion products were extracted twice with 0.1% trifluoroacetic acid, 50% acetonitrile, and 1.0% trifluoroacetic acid, respectively. The extracted mixture was dried by SpeedVac and dissolved in 10 μL of 0.1% trifluoroacetic acid for LC-MS/MS analysis^83^.

### Mass spectrometric and chromatographic methods and instrumentation

The gradient consisted of 5–35% (v/v) acetonitrile in 0.1% (v/v) formic acid at a flow rate of 200 nL/min for 10 min, 35–100% (v/v) acetonitrile in 0.1% (v/v) formic acid at a flow rate of 200 nL/min for 2 min and 100% acetonitrile in 0.1% formic acid at a flow rate of 200 nL/min for 8 min. The eluted peptides were ionized and introduced into a Thermo Fisher LTQ Velos Pro mass spectrometer (Thermo Fisher Scientific, Bremen, Germany) using a Proxeon nanoelectrospray ion source. Survey full-scan MS spectra (from *m*/*z* 200–1800) were acquired. A full mass spectrum (*m*/*z* 200–1800) was followed by fragmentation of the ten most abundant peaks, using 35% of the normalized collision energy for obtaining MS/MS spectra. All peptide assignments were verified by manual inspection. Data analyzed by Thermo Proteome Discoverer 1.4 software.

### Statistical analysis

All analyses were performed with the experimenter blinded to the groups of mice and cultured cells. Statistical comparisons were made with a two-tailed students’ t-test for two experimental groups or a one-way analysis of variance (ANOVA) with Tukey for multiple groups with SPSS software. For enzymatic activity analyses, Wilcoxon rank-sum test was performed. Statistical analysis comparing TUNEL^+^ cells between groups was performed using a two-tailed Mann–Whitney U Test. Statistical analyses were done using GraphPad Prism 8 (GraphPad Software).

### Reporting summary

Further information on research design is available in the Nature Portfolio Reporting Summary linked to this article.

## Supplementary information

Supplementary Information

Reporting Summary

## Data Availability

The hierarchical cluster heatmap, Venn diagram, and KEGG pathway data used in this study are available in the GSE18801 dataset (www.ncbi.nlm.nih.gov/geo/query/acc.cgi?acc=GSE18801). The mass spectrometry proteomics data have been deposited to the ProteomeXchange Consortium via the PRIDE partner repository with the dataset identifier PXD039587. Source data are provided as source data files. Additional relevant data are available at request. Source data are provided with this paper.

## Source data

Source Data
