# Supplementary material for: Fibroblast growth factor 18 alleviates stress-induced pathological cardiac hypertrophy in male mice
Source: Nat Commun. 2023 Mar 4;14:1235. doi: 10.1038/s41467-023-36895-1 (PMC9985628; doi:10.1038/s41467-023-36895-1)

**SUPPLEMENTAL MATERIAL**

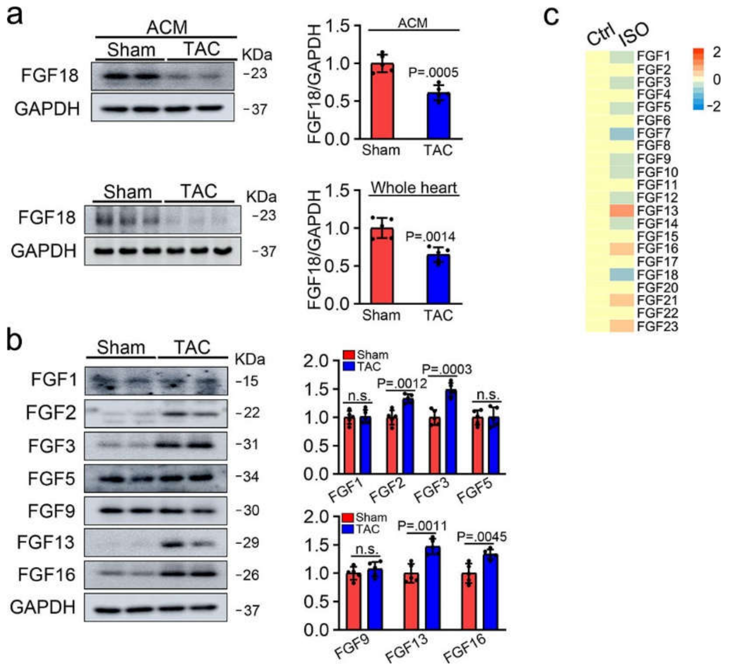


**Supplementary Fig. 1 FGF18 expression is decreased in NRCMs and hypertrophic murine hearts. a** Representative western blots; Quantitative results of FGF18 protein expression in isolated adult mouse cardiomyocytes and whole heart tissue after TAC for 6 weeks. n=5. Data represent means ± SD. Two-tailed student's t-test. **b** Representative western blots; Quantitative results of FGFs protein expression in whole heart tissue after TAC for 6 weeks. n=5. Data represent means ± SD. n.s. = not significant, Two-tailed student's t-test. **c** A profile of the mRNA expression of FGFs in NRCMs treated with ISO for 48 h. Heatmap depicts differentially expressed FGFs mRNA (applying the average fold change). Two-tailed student's t-test. All numbers (n) are biologically independent experiments. Source data are provided as a Source Data file.

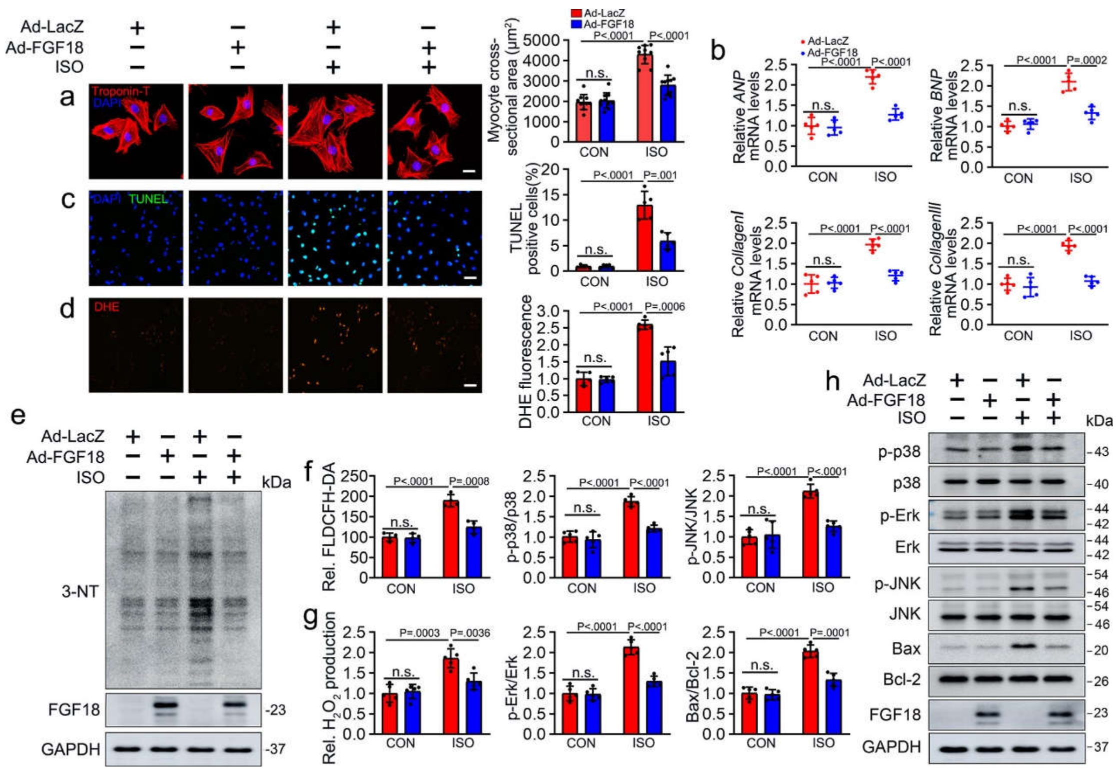

**Supplementary Fig. 2 FGF18 protects against ISO-induced cardiomyocyte hypertrophy *in vitro*.** NRCMs were infected with Ad-LacZ or Ad-FGF18 in the presence or absence of ISO (10 μM) for 48 h. **a** Representative images of NRCMs (blue: DAPI; red: cTnT; scale bar=20 μm). Quantification of cell surface area as shown in right panel, n=10. **b** Real-time quantitative PCR assays. n=5. **c** The apoptotic cells (green: TUNEL$^+$ cells , blue: DAPI). Scale bars = 45 μm. The quantitative analysis of TUNEL$^+$ cells (right panel). n=5. Two-tailed Non-parametric Mann-Whitney U test. **d** Fluorescent images and quantitation of superoxide levels (right panel). Scale bars = 130 μm. n=5. Two-tailed Non-parametric Mann-Whitney U test. **e** Levels of the oxidative damage marker 3-NT in NRCMs. **f** Total ROS levels (by DCFH-DA probe) were quantified. n=4. **g** Hydrogen peroxide levels (by Amplex Red regent) quantified in different groups of NRCMs. n=5. One-way ANOVA followed by a post-hoc Fisher's comparison test. **h** Cell lysate was analysed by western blotting with indicated antibodies. Quantification of relative protein levels in cardiomyocytes (left panel). n=5. n.s. = not significant. Quantitative data are reported as means ± SD, one-way ANOVA with Tukey multiple comparisons test. All numbers (n) are biologically independent experiments. Source data are provided as a Source Data file.

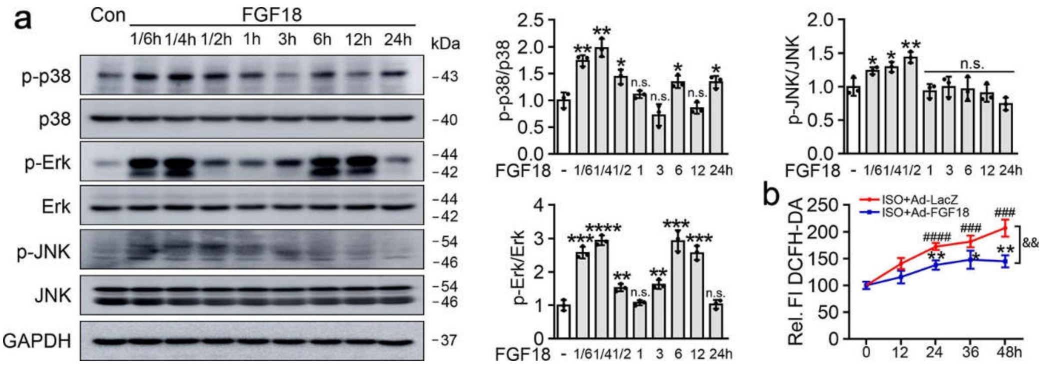


**Supplementary Fig. 3 FGF18 promotes downstream signaling pathways in time dependence.** NRCMs were treated with FGF18 (50 ng/mL) at different times. **a** Representative western blots; Quantitative results of p-p38, p-Erk, p-JNK protein expression in NRCMs in different hours. n=3. Data represent means ± SD. n.s. = not significant, (p-p38: 1/6h*p=0.0020, 1/4h*p=0.0016, 1/2h*p=0.0182, 6h*p=0.0327, 24h*p=0.0342; p-Erk: 1/6h*p=0.0003, 1/4h*p<0.0001, 1/2h*p=0.0090, 3h*p=0.0065, 6h*p=0.0007, 12h*p=0.0005; p-JNK: 1/6h*p=0.0440, 1/4h*p=0.0279, 1/2h*p=0.0083) *p versus NRCMs con. Two-tailed student's t-test. **b** Total ROS levels (by DCFH-DA probe) were quantified. n=3. One-way ANOVA followed by Turkey's post hoc test. Data represent means ± SD. 24h#p<0.0001, 36h#p=0.0002, 48h#p=0.0003 versus ISO/LacZ 0h; 24h*p=0.0039, 36h*p=0.0103, 48h*p=0.004 versus ISO/Ad-FGF18 0 h. &p=0.005. All numbers (n) are biologically independent experiments. Source data are provided as a Source Data file.

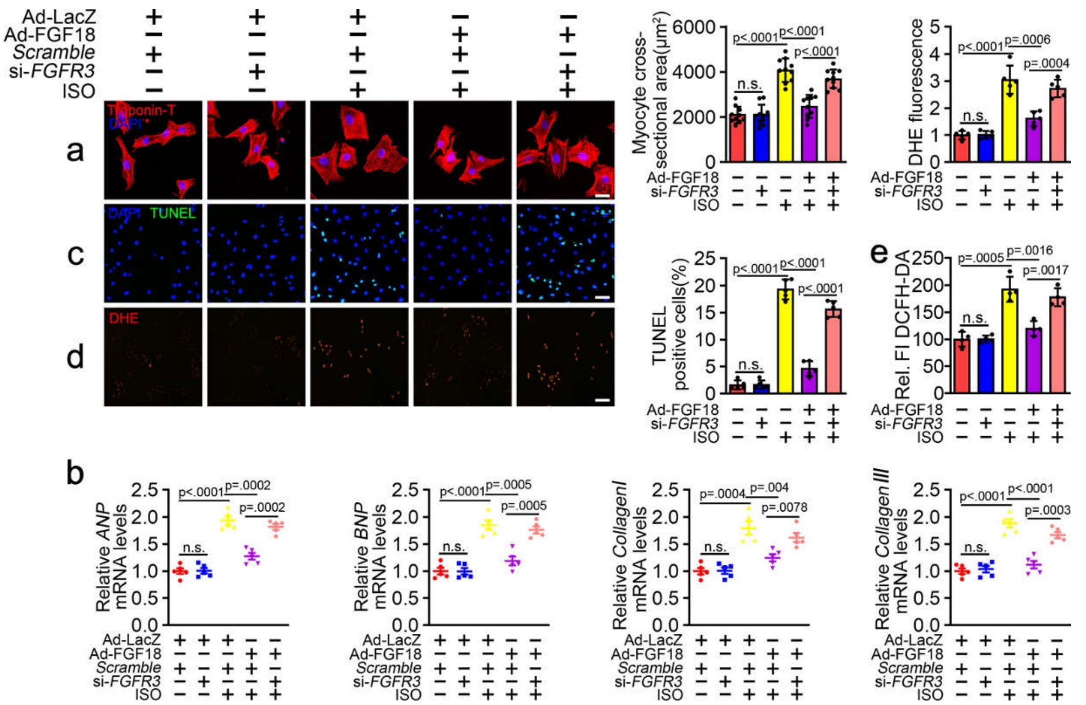


**Supplementary Fig. 4 The effect of FGF18 is receptor-dependent.** NRCMs were infected with Ad-LacZ or Ad-FGF18 in the presence or absence of ISO (10μM) and si-FGFR3 (1 mM) for 48 h. **a** Representative images of NRCMs (blue: DAPI; red: c-Troponin-T; scale bar=35 μm). Quantification of cell surface area as shown in right panel, n=10. **b** Real-time PCR assays were performed to determine the mRNA levels of ANP, BNP, collagen I and collagen III in NRCMs. n=5. **c** The apoptotic cells (green: TUNEL$^+$ cells , blue: DAPI). Scale bars = 45 μm. The quantitative analysis of TUNEL$^+$ cells (right panel). n=5. Two-tailed Non-parametric Mann-Whitney U test was used. **d** Fluorescent images and quantitation of superoxide levels (right panel). Scale bars = 130 μm. n=5. Two-tailed Non-parametric Mann-Whitney U test. **e** Total ROS levels (by DCFH-DA probe) were quantified. n=4. All quantitative data are reported as means ± SD, one-way ANOVA with Tukey multiple comparisons test: n.s. = not significant. All numbers (n) are biologically independent experiments. Source data are provided as a Source Data file.

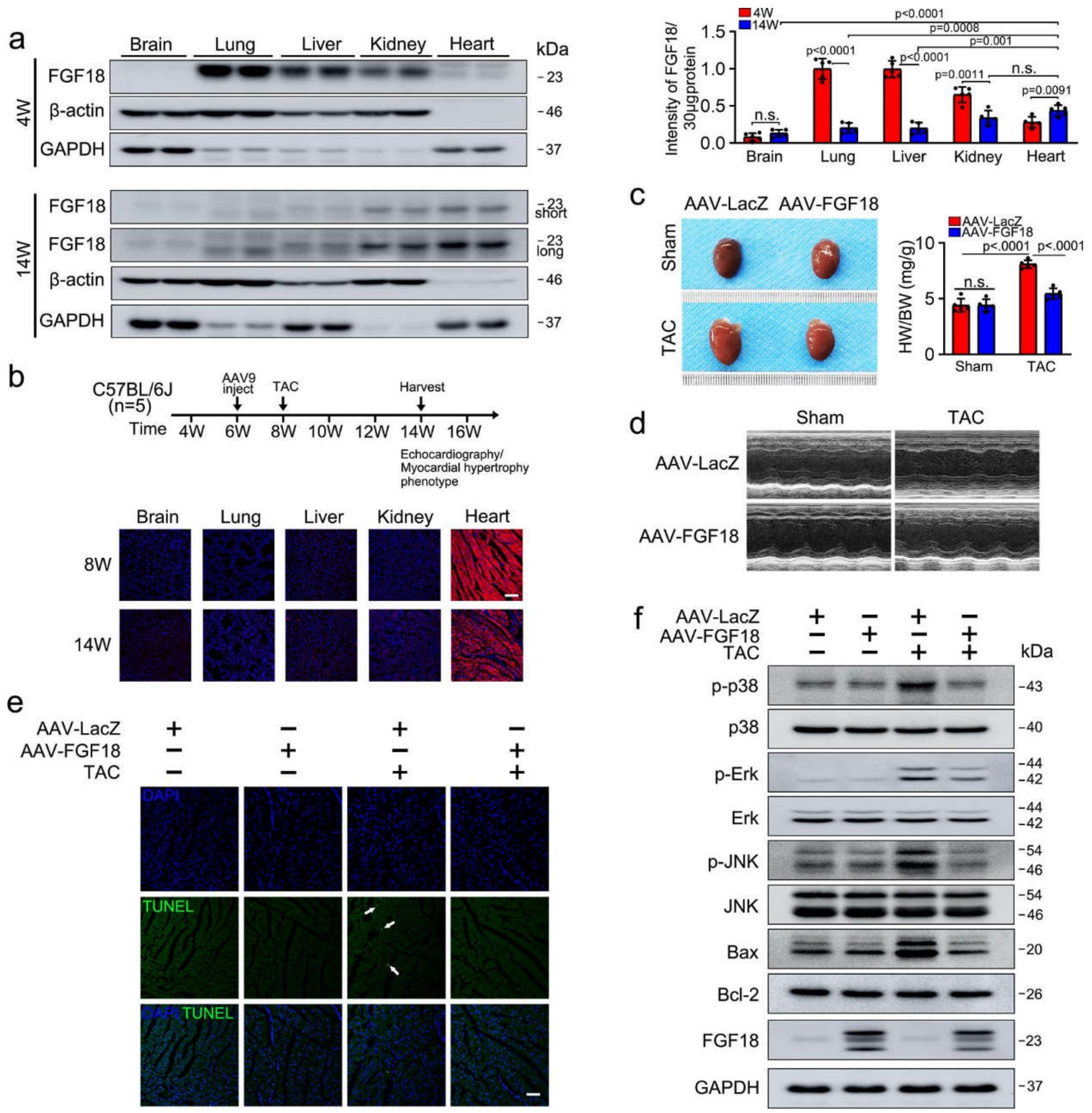


**Supplementary Fig. 5 Expression of FGF18 in different age's mice and the cardioprotection of FGF18 after Sham or TAC operation. a** Representative western blots; Quantitative results of FGF18 protein expression in different age's mice. n=5. Data represent means ± SD. n.s. = not significant. Two-tailed student's t-test. **b** Design of the study. $5\times10^{12}$ viral particles (AAV9-cTnT) were delivered to 6 weeks mice via intravenously tail veins injection. Two weeks later, mice were subjected to sham or TAC operation and sacrificed another 6 weeks later (after echocardiographic analysis). Representative confocal scans are shown for FGF18 overexpression in a variety of organs. Scale bars=110 μm. **c** Representative image of hearts from AAV9-LacZ and AAV9-cTnT-FGF18 mice following TAC or sham operation. Statistical results for the ratios of HW/BW in the indicated groups. n=5. Data are reported as means ± SD, one-way ANOVA with Tukey multiple comparisons test: n.s. = not significant. **d** Representative echocardiographic images for AAV9-LacZ and AAV9-cTnT-FGF18 mice after Sham or TAC operation. n=5. **e** Representative confocal scans are shown for TUNEL and DAPI (green and blue, respectively). n=5. Scale bars=45 μm. (Figure2). **f** Heart lysate was analysed by western blotting with indicated antibodies. n=5. Quantification of relative protein levels (Figure2). Source data are provided as a Source Data file.

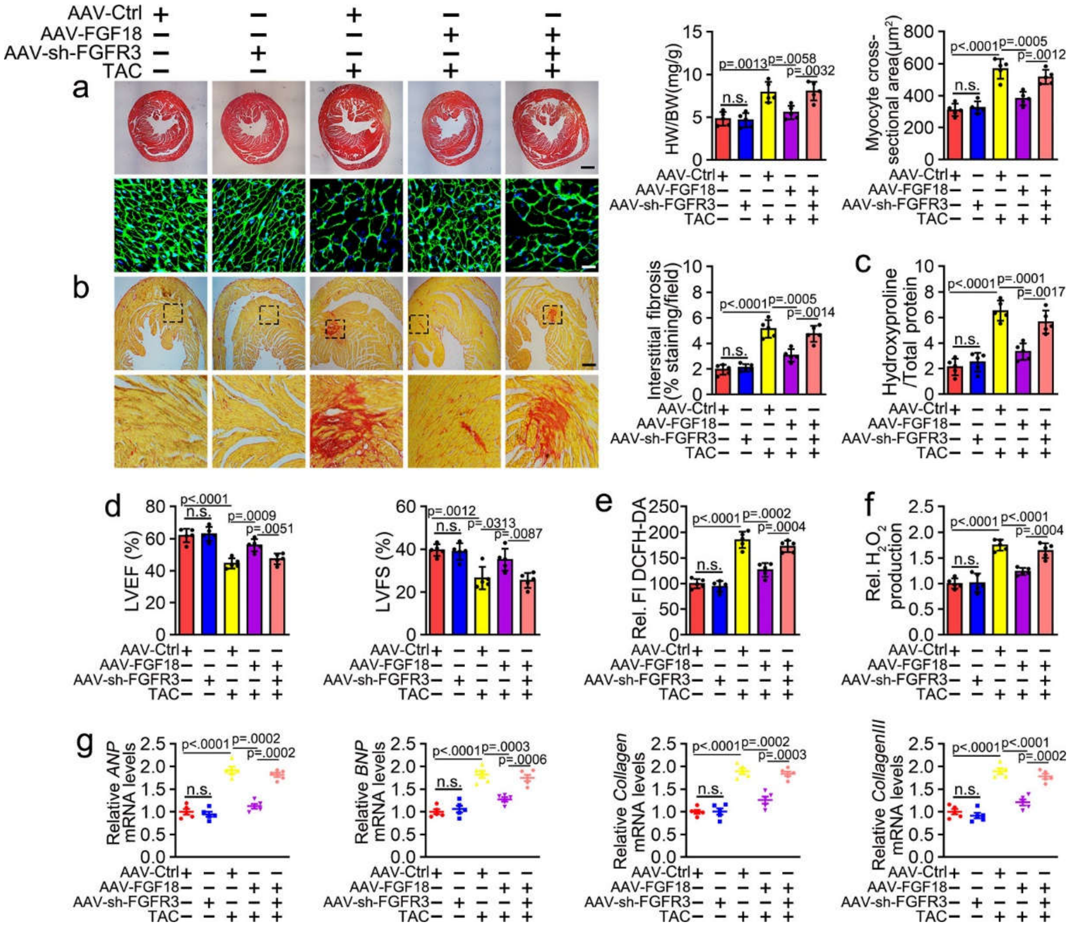

**Supplementary Fig. 6 The effect of FGF18 is receptor dependent in vivo.** FGF18 overexpression adeno-associated virus (AAV9-cTnT-FGF18) and control vector (AAV9-LacZ) were injected intravenously into tail veins of 6 weeks old male C57BL/6J mice respectively, one week after the injection, these mice were intravenously injected with the adeno-associated virus (AAV9-cTnT-sh-FGFR3; AAV9-Scramble) into tail veins, one week after the injection, these mice were subjected to Sham or TAC operation. **a** HE staining and WGA staining. (n=5. scale bar=0.6 mm for upper HE staining; scale bar=20 μm for lower WGA staining). Statistical results for the ratios of HW/BW and the cell sectional area (down panel). n=5. **b** PSR staining and quantification (down panel). n=5. Scale bar=450 μm and then zoom in 5 times. **c** Left ventricular collagen quantification by hydroxyproline assay (μg/mg). n=5. **d** Echocardiographic measurement of LVEF and LVFS are shown. n=5. **e** Total ROS levels (by DCFH-DA probe) were quantified. n=5. **f** Hydrogen peroxide levels (by Amplex Red assay) quantified in different groups. n=5. One-way ANOVA was followed by a post-hoc Fisher's comparison test. **g** Real-time quantitative PCR assays. n=5. All quantitative data are reported as means ± SD, one-way ANOVA with Tukey multiple comparisons test: n.s. = not significant. All numbers (n) are biologically independent animals. Source data are provided as a Source Data file.

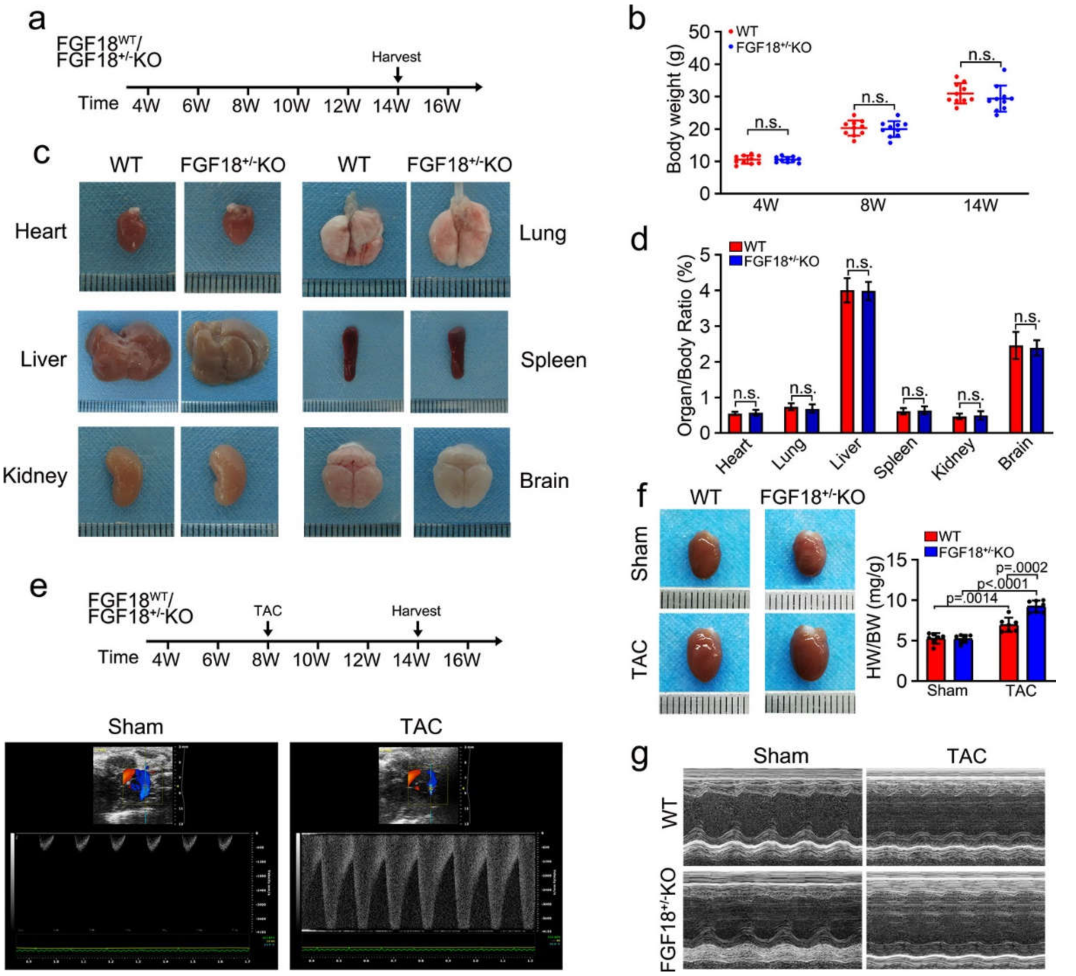


**Supplementary Fig. 7 Characterization of FGF18 systemic semi-knockout mice (*Fgf18*$^{+/-}$KO mice). a** FGF18 heterozygous mice (*Fgf18*$^{+/-}$*KO*) were sacrificed at 14 weeks old male mice. **b** Body weight of WT and *Fgf18*$^{+/-}$KO littermates at indicated times. n=10/group. Data are reported as means ± SD. Two-tailed Mann-Whitney *U* test. n.s., no significant difference. (**c** and **d**) Gross appearance **c** and quantification of organ to body ratio **d** of hearts, lungs, livers, spleens, kidneys and brains isolated from WT and *Fgf18*$^{+/-}$KO littermates. n=10/group. Data are reported as means ± SD. Two-tailed Mann-Whitney *U* test. n.s., no significant difference. **e** FGF18 heterozygous mice (*Fgf18*$^{+/-}$*KO*) were subjected to Sham or TAC operation and sacrificed another 6 weeks later (after echocardiographic analysis); Echocardiograph was used to measure the right-to-left carotid artery flow velocity ratio after TAC, and the mice with a carotid artery flow velocity ratio > 4000 mm/s were regarded as eligible models. **f** Representative image of hearts from WT and *Fgf18*$^{+/-}$KO mice following TAC or sham operation. Statistical results for the ratios of HW/BW in the indicated groups. n=7. Data are reported as means ± SD, one-way ANOVA with Tukey multiple comparisons test. **g** Representative echocardiographic images for WT and *Fgf18*$^{+/-}$KO mice after Sham or TAC operation. n=7. Source data are provided as a Source Data file.

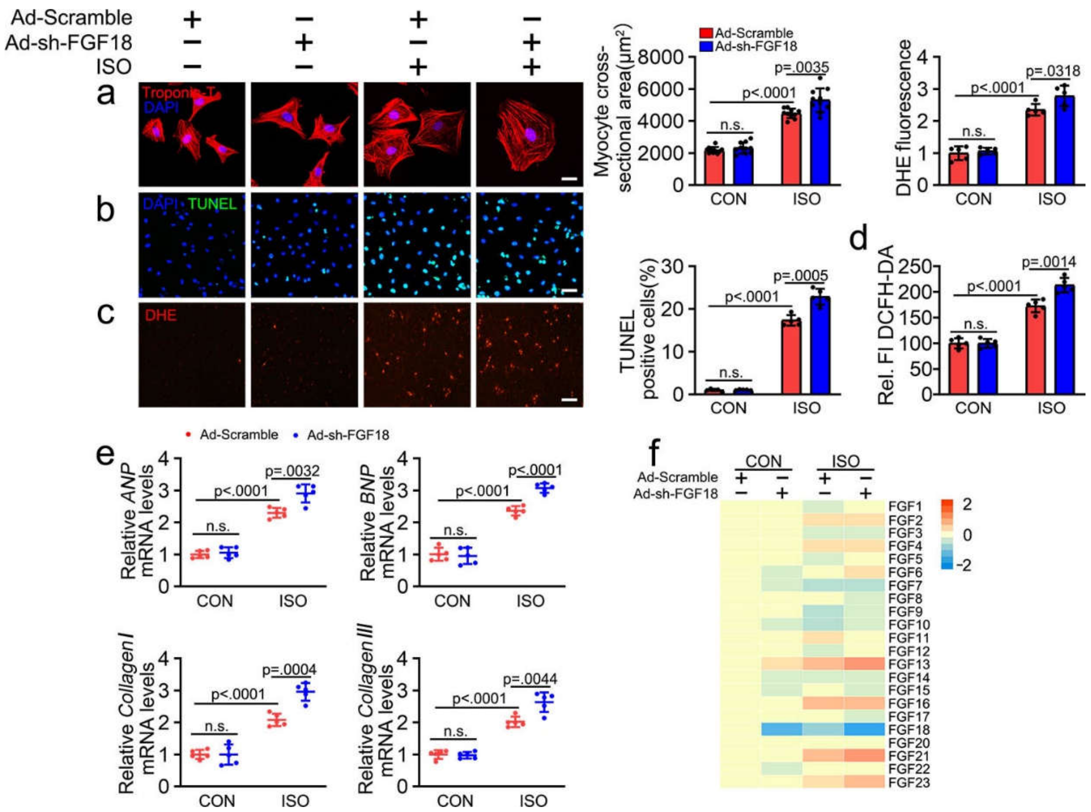


**Supplementary Fig. 8 FGF18 deficiency aggravates ISO-induced hypertrophy.** NRCMs were infected with Ad-Scramble or Ad-sh-FGF18 in the presence or absence of ISO (10 μM) for 48 h. **a** Representative images of NRCMs (DAPI: nuclear; red: c-Troponin-T; scale bar=20 μm). Quantification of cell surface area as shown in right panel, n=10. **b** The apoptotic cells (green: $TUNEL^+$ cells , blue: DAPI). Scale bars = 45 μm. The quantitative analysis of $TUNEL^+$ cells (right panel). n=5. Two-tailed Non-parametric Mann-Whitney U test was used. **c** Fluorescent images and quantitation of superoxide levels (right panel). Superoxide was determined with the fluorescent indicator DHE. Scale bars = 130 μm. n=5. Two-tailed Non-parametric Mann-Whitney U test. **d** Total ROS levels (by DCFH-DA probe) were quantified. n=5. **e** Real-time PCR assays. n=5. **f** A profile of the mRNA expression of FGFs in NRCMs. Heatmap depicts differentially expressed FGFs mRNA (applying the average fold change). Two-tailed student's t-test. All quantitative data are reported as means ± SD, one-way ANOVA with Tukey multiple comparisons test. All numbers (n) are biologically independent experiments. Source data are provided as a Source Data file.

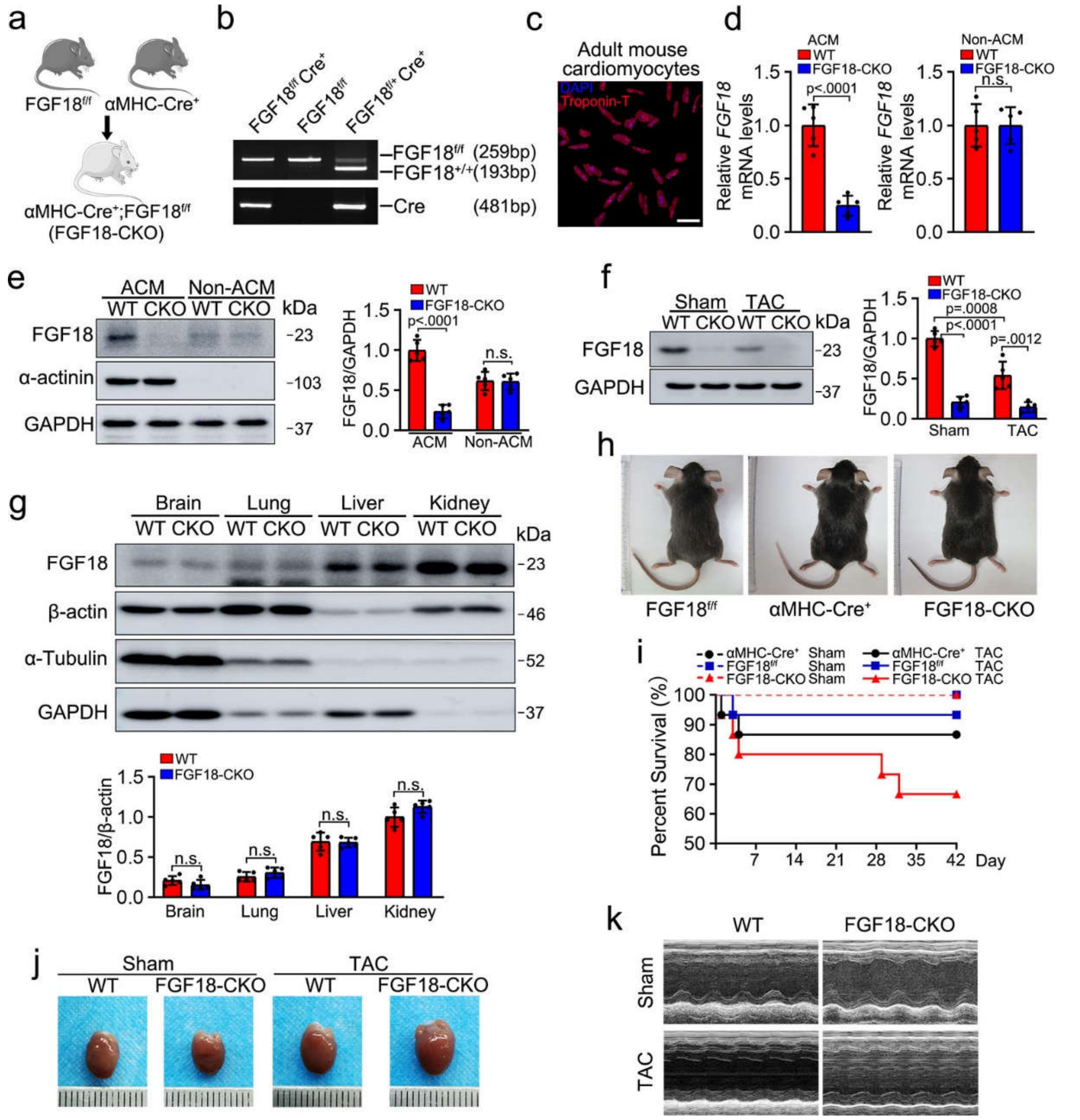


**Supplementary Fig. 9 Generation of cardiomyocyte-specific FGF18 knockout mice (αMHC-MerCreMer, *Fgf18*$^{f/f}$; *Fgf18*-CKO) and genotype identification.** All mice were intraperitoneally injected with tamoxifen at 6 weeks old male mice, and kept for a 14-day waiting period to get the efficient gene knockout. **a** Diagram showing the strategy for the generation of *Fgf18*-CKO mice. *Fgf18*-CKO mice were obtained by crossing FGF18$^{f/f}$ mice with αMHC-Cre mice. Heterozygous animals of both genotypes were mated to generate embryos. The figure is modified from Servier Medical Art (http://smart.servier.com/), licensed under a Creative Common Attribution 3.0 unported License. **b** Genotyping results of wild-type, heterozygous and FGF18 knockout mice. *n*=3. **c** Immunofluorescence images of c-Troponin-T (red) staining of adult mouse cardiomyocytes (ACM). Scale bar=30 μm. **d** Quantitative real-time reverse transcription-PCR. *n=5*. Data are reported as means ± SD. Two-tailed Mann-Whitney U test. **e** FGF18 protein expression in adult mouse cardiomyocytes (ACM) and non-cardiomyocytes cells (non-ACM) isolated from wild-type (WT, *Fgf18*$^{f/f}$) or *Fgf18*-CKO mice. Left, representative Western blot showing FGF18 protein expression; right, Quantification of FGF18 protein levels in cardiomyocytes. n=5. Data are reported as means ± SD. Two-tailed student's t-test. **f** FGF18 protein expression in WT (*Fgf18*$^{f/f}$) and *Fgf18*-CKO mouse hearts subjected to TAC or sham

operation. Left, representative immunoblot showing decreased FGF18 protein expression; right, Quantification of FGF18 protein levels in cardiomyocytes. n=5. Data are reported as means ± SD. **g** Immunoblotting analysis and quantification results of FGF18 protein expression in different organ tissues from 14-weeks WT (*Fgf18*$^{f/f}$) and *Fgf18*-CKO mice. n=5. Data are reported as means ± SD. n.s., no significant difference. Two-tailed student's t-test. **h** The proportion of born WT (*Fgf18*$^{+/+}$), heterozygous (αMHC-MerCreMer; *Fgf18*$^{f/+}$), and homozygous (αMHC-MerCreMer; *Fgf18*$^{f/f}$) mice complied with Mendel's laws, and their appearance was undistinguishable from the littermates. **i** Cumulative survival rate of α-MHC-MerCreMer, *Fgf18*$^{f/f}$ and *Fgf18*-CKO mice, was subjected to Sham or TAC surgery for 6 weeks, n=10. **j** Representative image of hearts from WT and *Fgf18*-CKO mice following TAC or sham operation. n=5. **k** Representative echocardiographic images for WT (*Fgf18*$^{f/f}$) and *Fgf18*-CKO mice after Sham or TAC operation. n=5. All numbers (n) are biologically independent experiments. Source data are provided as a Source Data file.

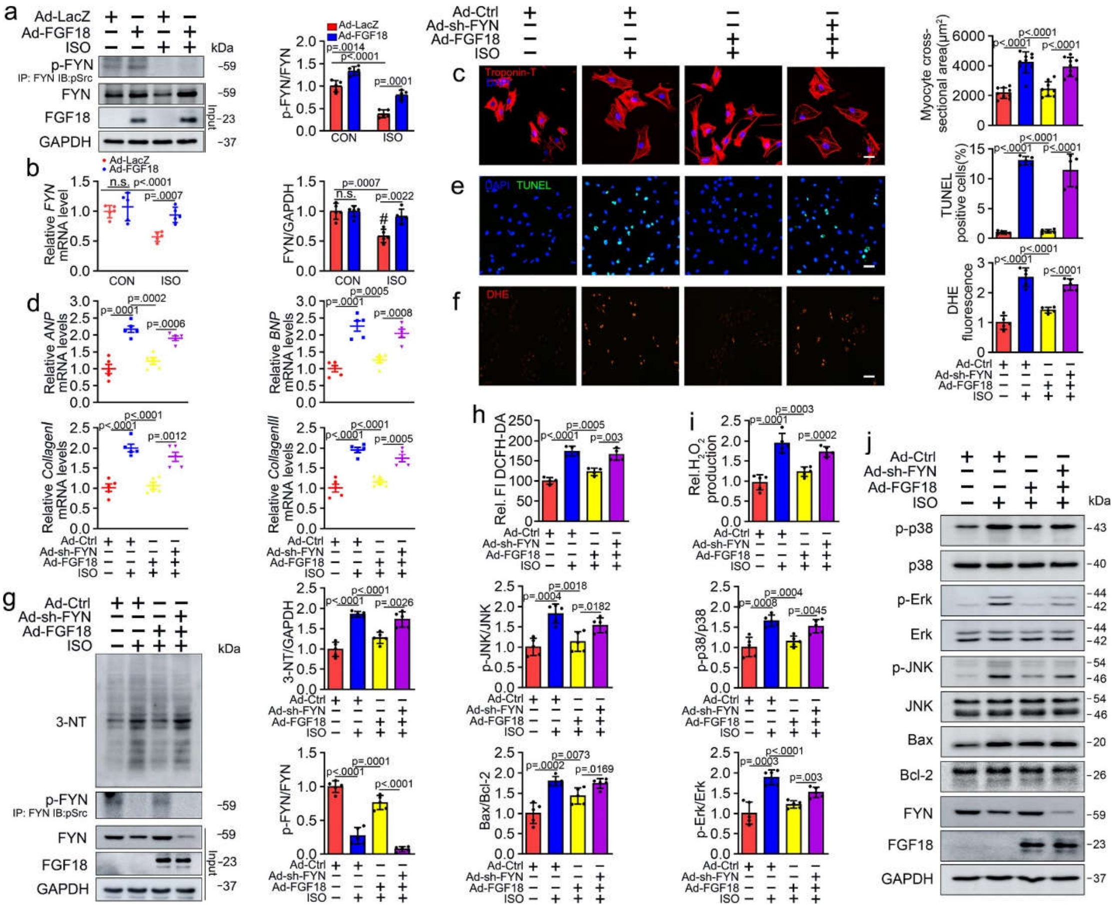


**Supplementary Fig. 10 FGF18 attenuates pressure overload-induced cardiac hypertrophy requires FYN. a** NRCMs infected with Ad-LacZ or Ad-FGF18 in the presence or absence of ISO (10 μM) for 48 h. Expression levels of phosphorylated FYN, total FYN and GAPDH were evaluated with immunoblotting. After immunoprecipitation (IP) with an anti-FYN antibody, immunoblot analyses (IB) for phospho-Src (S416) were performed to detect FYN phosphorylated at the tyrosine in the activation loop of the kinase domain (p-FYN). In the quantitative analysis, the level of FYN was analysed and normalized with GAPDH (right panel), the level of p-FYN detected by the anti-phospho-Src antibody/total FYN. n=5. **b** qRT-PCR analysis of FYN levels in NRCMs infected with Ad-LacZ or Ad-FGF18 in the presence or absence of ISO (10 μM) for 48 h. n=5. (**a-b**) Data are reported as means ± SD, one-way ANOVA with Tukey multiple comparisons test. (**c-j**) NRCMs infected with Ad-LacZ or Ad-FGF18 and Ad-Scramble or Ad-sh-FYN were subjected to ISO (10 μM) for 48 h. **c** Representative images of NRCMs (blue: DAPI; red: c-Troponin-T; scale bar=35 μm). Quantification of cell surface area as shown in right panel, n=9. **d** Real-time PCR assays. n=5. **e** The apoptotic cells (green: TUNEL$^{+}$ cells , blue: DAPI). Scale bars = 45 μm. The quantitative analysis of TUNEL$^{+}$ cells (right panel). n=5. Two-tailed Non-parametric Mann-Whitney U test was used. **f** Fluorescent images and quantitation of superoxide levels (right panel). Scale bars = 130 μm. n=5. Two-tailed Non-parametric Mann-Whitney U test. **g** Cell lysate was analysed by immunoprecipitation and western blotting with indicated antibodies. The quantitative analysis of 3-NT and p-FYN (detected by the p-Src/total FYN) protein immunoblots as shown in right panel, n=5. **h** Total ROS levels (by DCFH-DA probe) were quantified. n=4. **i** Hydrogen peroxide levels (by Amplex Red assay) quantified in different groups. n=5. One-way ANOVA followed by a post-hoc Fisher's comparison

test. **j** Cell lysate was analysed by western blotting with indicated antibodies. Quantification of relative protein levels in cardiomyocytes (left panel). n=5. (**c-j**) Data are reported as means ± SD, one-way ANOVA with Tukey multiple comparisons test. All numbers (n) are biologically independent experiments. Source data are provided as a Source Data file.

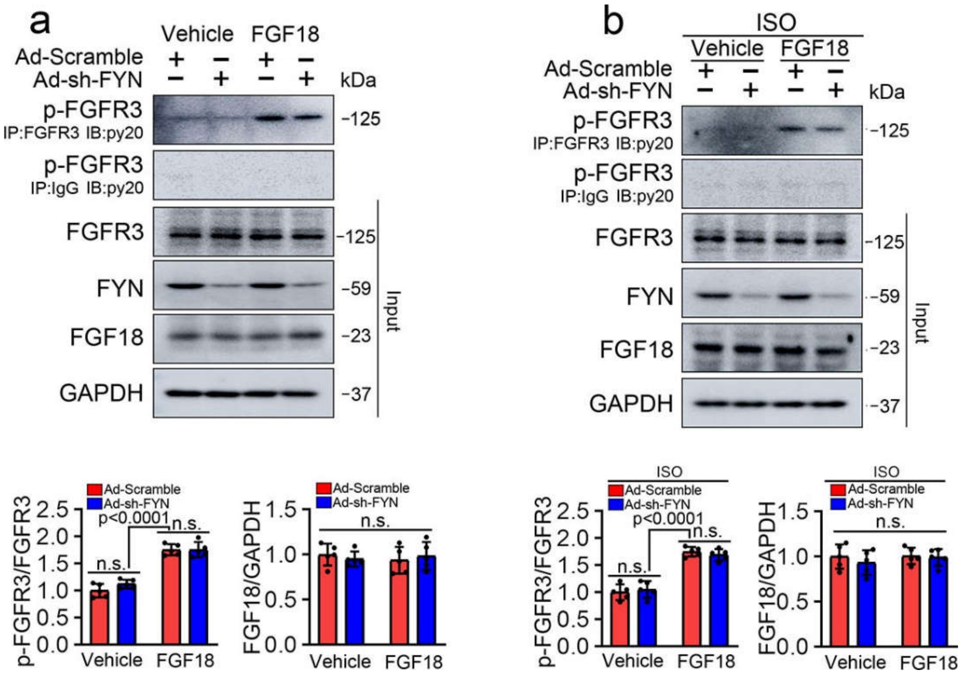


**Supplementary Fig. 11 No feedback effects were observed by silencing of FYN in NRCMs. a-b** NRCMs were infected with Ad-Scramble or Ad-sh-FYN and treatment with FGF18 (50 ng/mL) in the presence or absence of ISO (10 μM) for 48 h. Immunoprecipitation (IP) with an anti-FGFR3 antibody, and immunoblot analyses (IB) for py20 were performed to detect FGFR3 phosphorylated at the tyrosine. The cell lysate was analysed by western blotting with indicated antibodies for input. Quantification of relative protein levels in NRCMs (down panel). n=5. Data are reported as means ± SD, one-way ANOVA with Tukey multiple comparisons test: n.s. = not significant. All numbers (n) are biologically independent experiments. Source data are provided as a Source Data file.

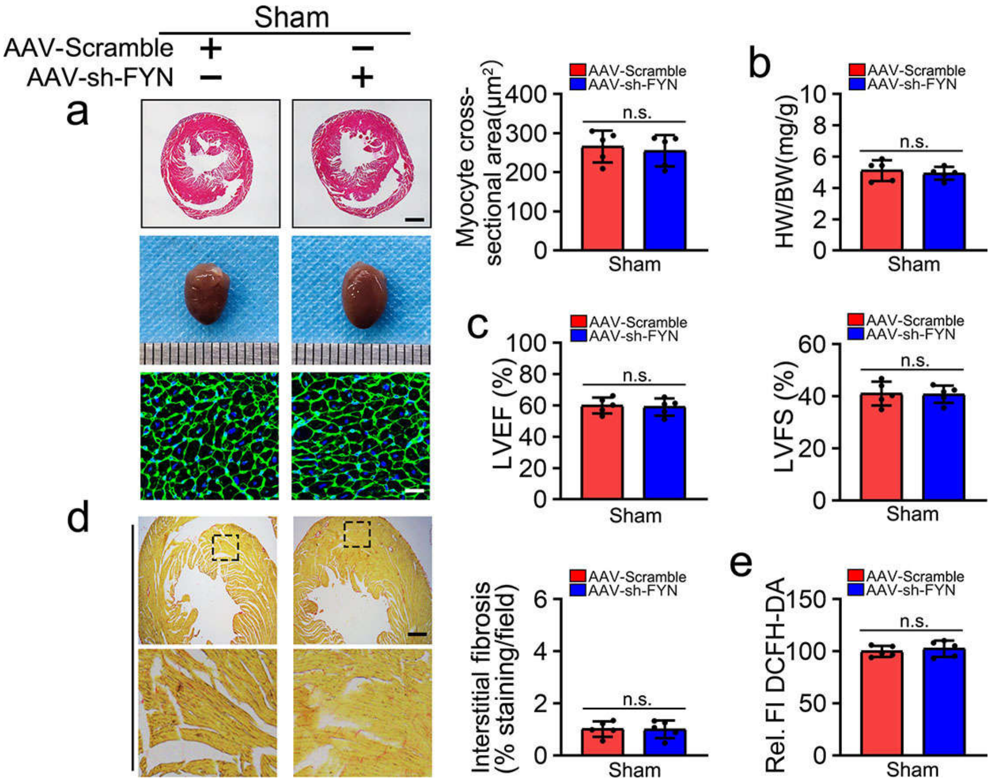


**Supplementary Fig. 12 FYN-deficient mice did not show cardiac phenotypic differences at the basal level.** Six weeks old male C57BL/6J mice were intravenously injected with the adeno-associated virus (AAV9-cTnT-sh-FYN; AAV9-Scramble) into tail veins, two weeks after the injection, these mice were subjected to Sham operation. **a** Histological analysis of the HE staining and WGA staining. (n=5. scale bar=0.6 mm for upper HE staining; scale bar=20 μm for lower WGA staining). Representative image of hearts from injected mice following Sham operation. n=5. Statistical results for the cell sectional area (down panel). n=5. **b** Statistical results for the ratios of HW/BW in the indicated groups. n=5. **c** Echocardiographic measurement of left ventricular ejection fraction [LVEF=(EDV-ES)/EDV] and left ventricular fractional shortening [LVFS=(LVEDD-LVESD)/LVEDD×100%] are shown. n=5.**d** PSR staining and quantification (right panel). n=5. Scale bar=450 μm and then zoom in 5 times. **e** Total ROS levels (by DCFH-DA probe) were quantified. n=5. All quantitative data are reported as means ± SD, two-tailed student's t-test: n.s. = not significant. All numbers (n) are biologically independent animals. Source data are provided as a Source Data file.

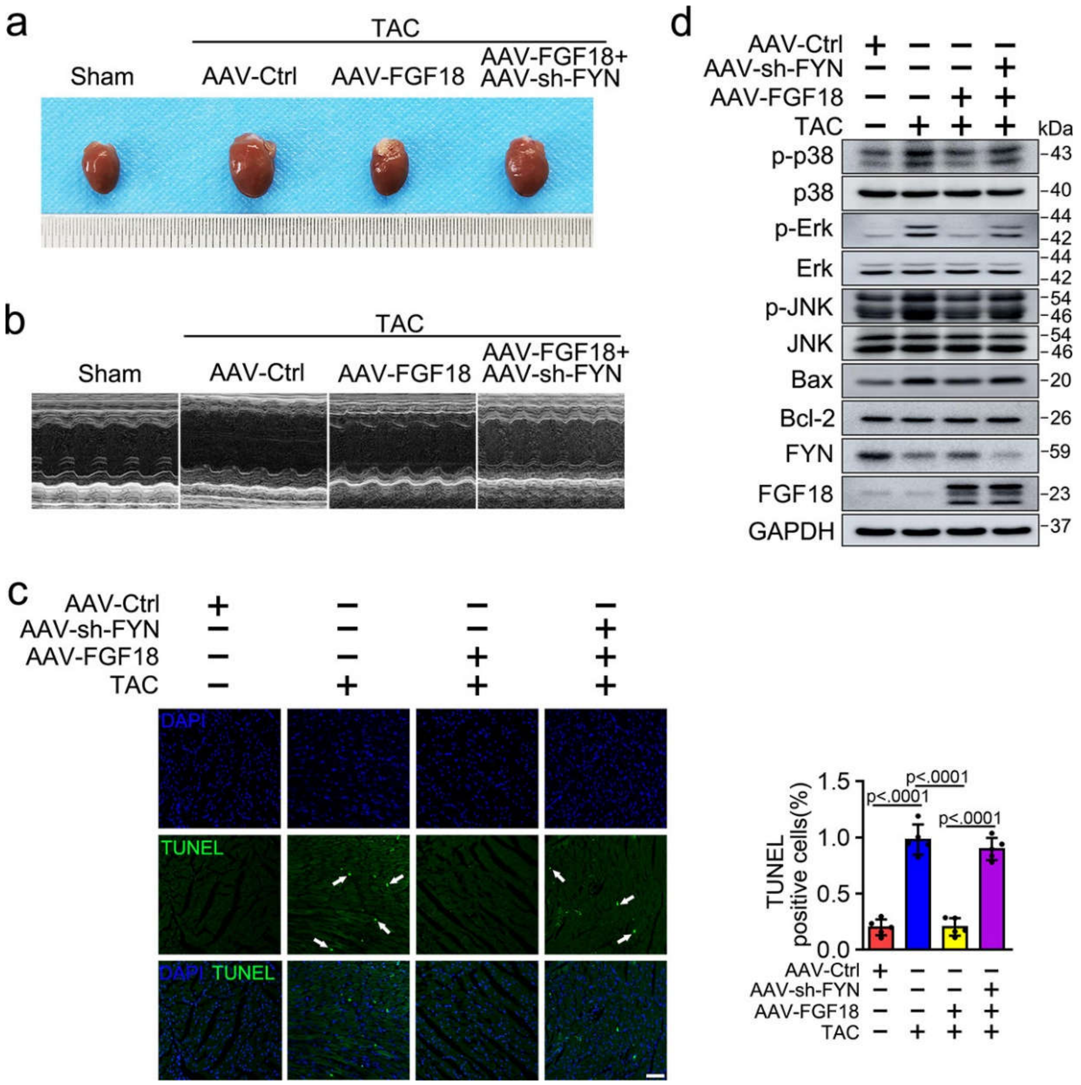


**Supplementary Fig. 13 Loss of FYN abolishes the cardioprotective effects of FGF18 *in vivo*.** FGF18 overexpression adeno-associated virus (AAV9-cTnT-FGF18) and control vector (AAV9-LacZ) were injected intravenously into tail veins of 6 weeks old male C57BL/6J mice respectively, one week after the injection, these mice were intravenously injected with the adeno-associated virus (AAV9-cTnT-sh-FYN; AAV9-Scramble) into tail veins, one week after the injection, these mice were subjected to Sham or TAC operation. **a** Representative image of hearts from injected mice following TAC or sham operation. n=5. **b** Representative echocardiographic images for AAV9-Ctrl, AAV9-FGF18 and AAV9-sh-FYN mice after Sham or TAC operation. **c** Representative confocal scans are shown for TUNEL and DAPI (green and blue, respectively). The histogram (right panel) is the quantitative analysis of TUNEL$^{+}$ cells in at least six separate fields. n=5. (biologically independent experiments). Scale bars=45 μm. Quantitative data are reported as means ± SD. Two-tailed Non-parametric Mann-Whitney U test. **d** Heart lysate was analysed by western blotting with indicated antibodies. n=5. Quantification of relative protein levels (Figure 6). Source data are provided as a Source Data file.

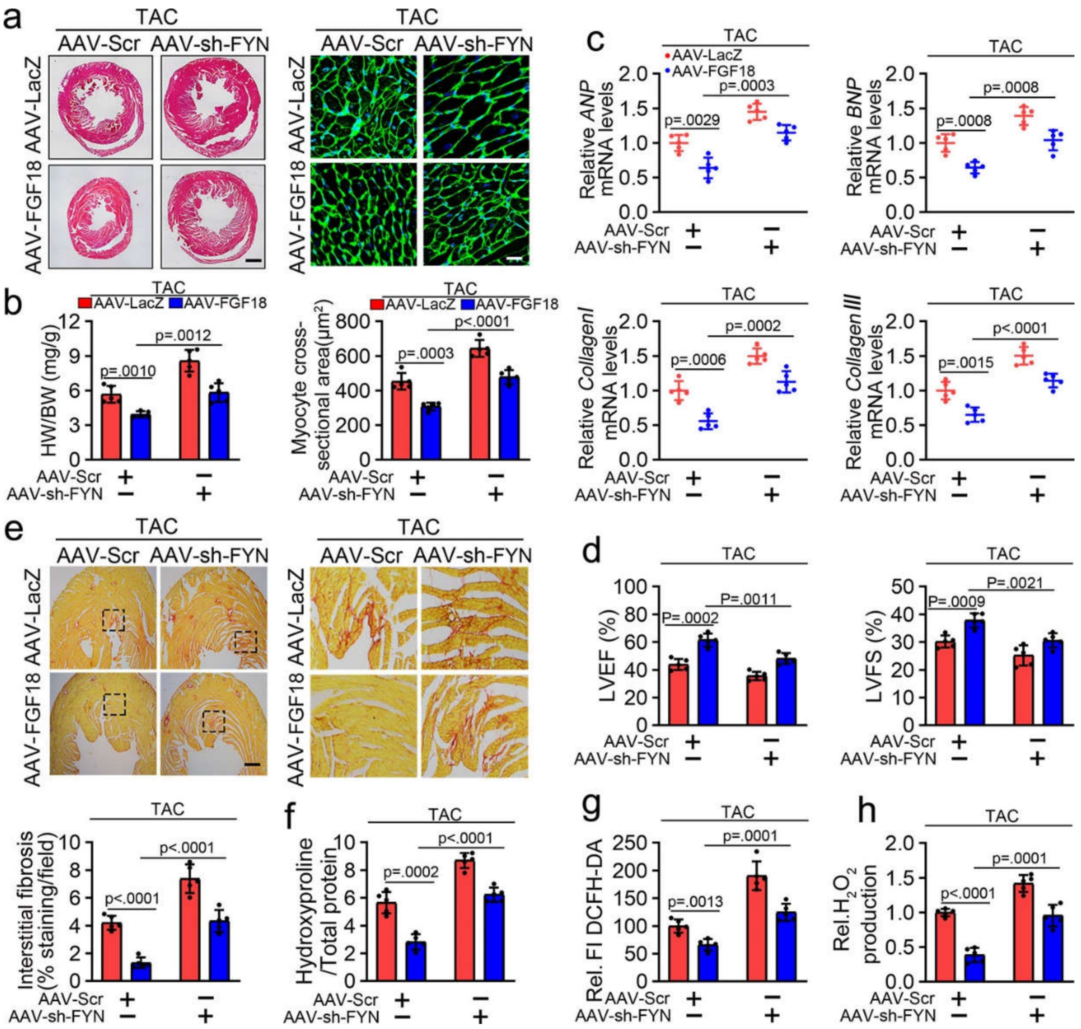

**Supplementary Fig. 14 FYN deletion aggravates TAC-induced hypertrophy.** FGF18 overexpression adeno-associated virus (AAV9-cTnT-FGF18) and control vector (AAV9-LacZ) were injected intravenously into tail veins of 6 weeks old male C57BL/6J mice respectively, one week after the injection, these mice were intravenously injected with the adeno-associated virus (AAV9-cTnT-sh-FYN; AAV9-Scramble) into tail veins, one week after the injection, these mice were subjected to TAC operation. **a** Histological analysis of the HE staining and WGA staining. (n=5. scale bar=0.6 mm for upper HE staining; scale bar=20 μm for lower WGA staining). Representative image of hearts from injected mice following TAC or sham operation. n=5. Statistical results for the cell sectional area (down panel). n=5. **b** Statistical results for the ratios of HW/BW in the indicated groups. n=5. **c** Real-time quantitative PCR assays. n=5. **d** Echocardiographic measurement of left ventricular ejection fraction [LVEF=(EDV-ES)/EDV] and left ventricular fractional shortening [LVFS=(LVEDD-LVESD)/LVEDD×100%] are shown. n=5. **e** PSR staining and quantification (down panel). n=5. Scale bar=450 μm and then zoom in 5 times. **f** Left ventricular collagen quantification by hydroxyproline assay (μg/mg). n=5. **g** Total ROS levels (by DCFH-DA probe) were quantified. n=5. **h** Hydrogen peroxide levels (by Amplex Red assay) quantified in different groups. n=5. One-way ANOVA was followed by a post-hoc Fisher's comparison test. All quantitative data are reported as means ± SD, one-way ANOVA with Tukey multiple comparisons test: n.s. = not significant. All numbers (n) are biologically independent animals. Source data are provided as a Source Data file.

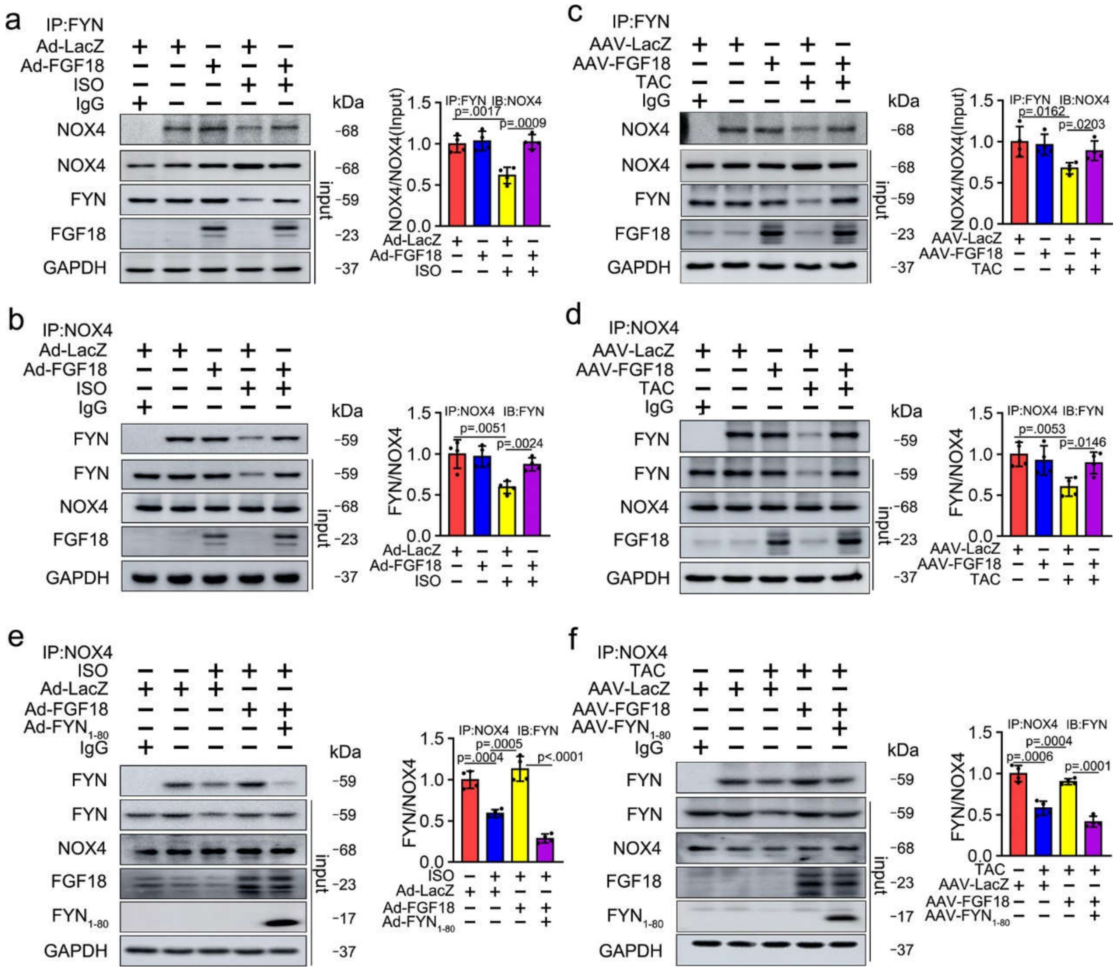


**Supplementary Fig. 15 FGF18 attenuates cardiac hypertrophy by FYN through a mechanism involving NOX4.** (**a-b**) NRCMs were infected with Ad-LacZ or Ad-FGF18 in the presence or absence of ISO (10μM) for 48 h. **a** The cell lysates were subjected to immunoprecipitation with FYN antibody in NRCMs, followed by immunoblotting with the NOX4 antibody. n=4. **b** The cell lysates were subjected to immunoprecipitation with NOX4 antibody in NRCMs, followed by immunoblotting with the FYN antibody. IgG as the negative control. n=4. (**c-d**) FGF18 overexpression vector (AAV9-cTnT-FGF18) and control vector (AAV9-LacZ) were injected intravenously into the tail veins of 6 weeks old male C57BL/6J mice respectively, two weeks after the injection, these mice were subjected to Sham or TAC operation. **c** The heart lysates were subjected to immunoprecipitation with FYN antibody, followed by immunoblotting with the NOX4 antibody. n=4. **d** The heart lysates were subjected to immunoprecipitation with NOX4 antibody, followed by immunoblotting with the FYN antibody. IgG as the negative control. n=4. **e** NRCMs were infected with Ad-LacZ, Ad-$FYN_{1-80}$ or Ad-FGF18 in the presence or absence of ISO (10μM) for 48 h. The cell lysates were subjected to immunoprecipitation with NOX4 antibody in NRCMs, followed by immunoblotting with the FYN antibody. IgG as the negative control. n=4. **f** FGF18 overexpression adeno-associated virus (AAV9-cTnT-FGF18) and control vector (AAV9-LacZ) were injected intravenously into tail veins of 6 weeks old male C57BL/6J mice respectively, one week after the injection, these mice were intravenously injected with the adeno-associated virus (AAV9-cTnT-$FYN_{1-80}$) into tail veins, one week after the injection, these mice were subjected to Sham or TAC operation. The heart lysates were subjected to

immunoprecipitation with NOX4 antibody, followed by immunoblotting with the NOX4, FYN antibody. IgG as the negative control. n=4. All quantitative data are reported as means ± SD, one-way ANOVA with Tukey multiple comparisons test. All numbers (n) are biologically independent experiments. Source data are provided as a Source Data file.

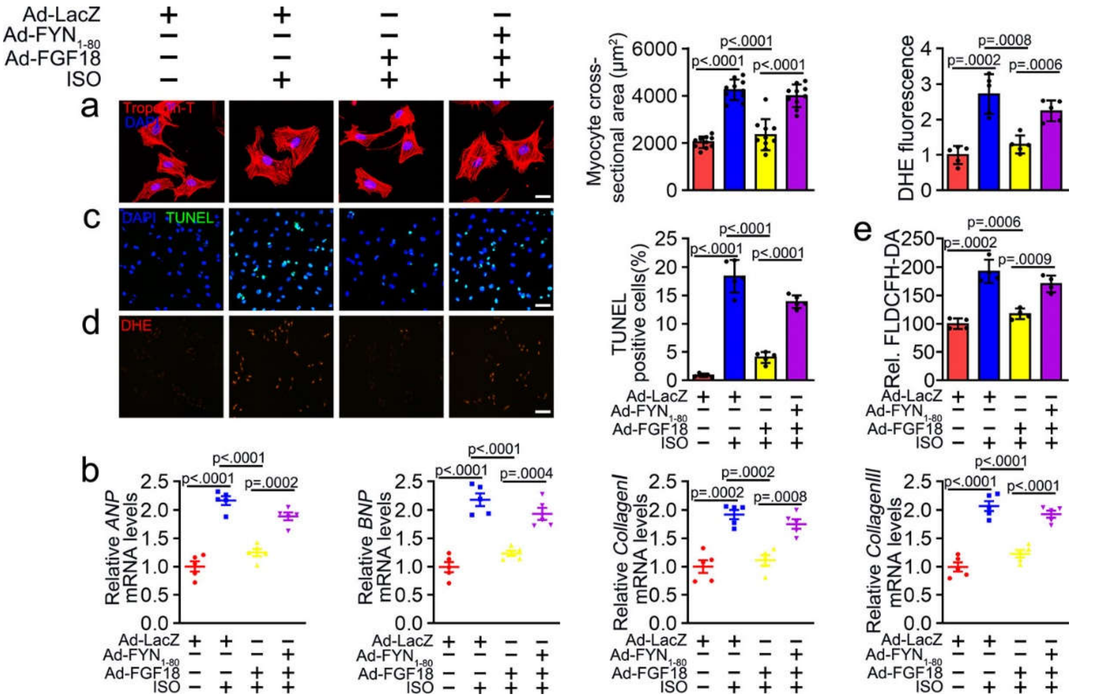


**Supplementary Fig. 16 FYN1-80 aggravates ISO-induced cardiomyocyte hypertrophy via interfering with the interaction between endogenous FYN and NOX4.** NRCMs were infected with Ad-LacZ, Ad-FGF18 and Ad-FYN1-80 in the presence or absence of ISO (10 μM) and for 48 h. **a** Representative images of NRCMs (blue: DAPI; red: c-Troponin-T; scale bar=30 μm). Quantification of cell surface area as shown in right panel, n=10. **b** Real-time PCR assays. n=5. **c** The apoptotic cells (green: TUNEL$^{+}$ cells , blue: DAPI). Scale bars = 45 μm. The quantitative analysis of TUNEL$^{+}$ cells (right panel). n=5. Two-tailed Non-parametric Mann-Whitney U test was used. **d** Fluorescent images and quantitation of superoxide levels (right panel). Scale bars = 130 μm. n=5. Two-tailed Non-parametric Mann-Whitney U test. **e** Total ROS levels (by DCFH-DA probe) were quantified. n=4. All quantitative data are reported as means ± SD, one-way ANOVA with Tukey multiple comparisons test. All numbers (n) are biologically independent experiments. Source data are provided as a Source Data file.

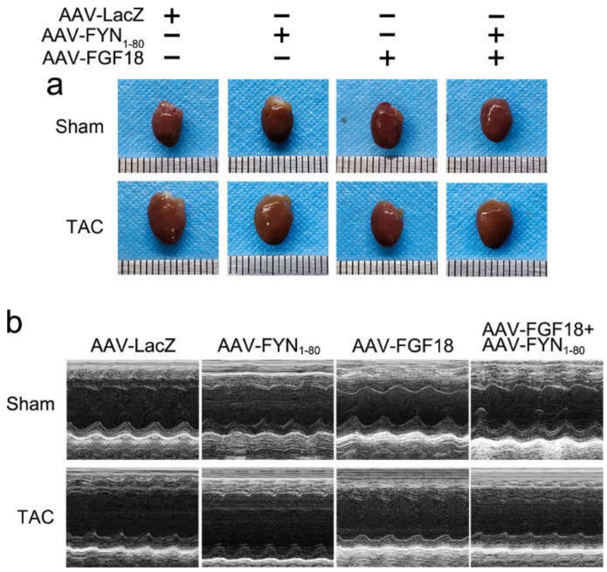


**Supplementary Fig. 17 FYN$_{1\text{-}80}$ aggravates pressure overload-induced hypertrophy. a** Representative image of hearts from AAV9-LacZ, AAV9-cTnT-FGF18 and AAV9-cTnT-FYN$_{1\text{-}80}$ injected mice respectively following TAC or sham operation. n=5. **b** Representative echocardiographic images for AAV9-LacZ, AAV9-cTnT-FGF18 and AAV9-cTnT-FYN$_{1\text{-}80}$ mice after Sham or TAC operation.

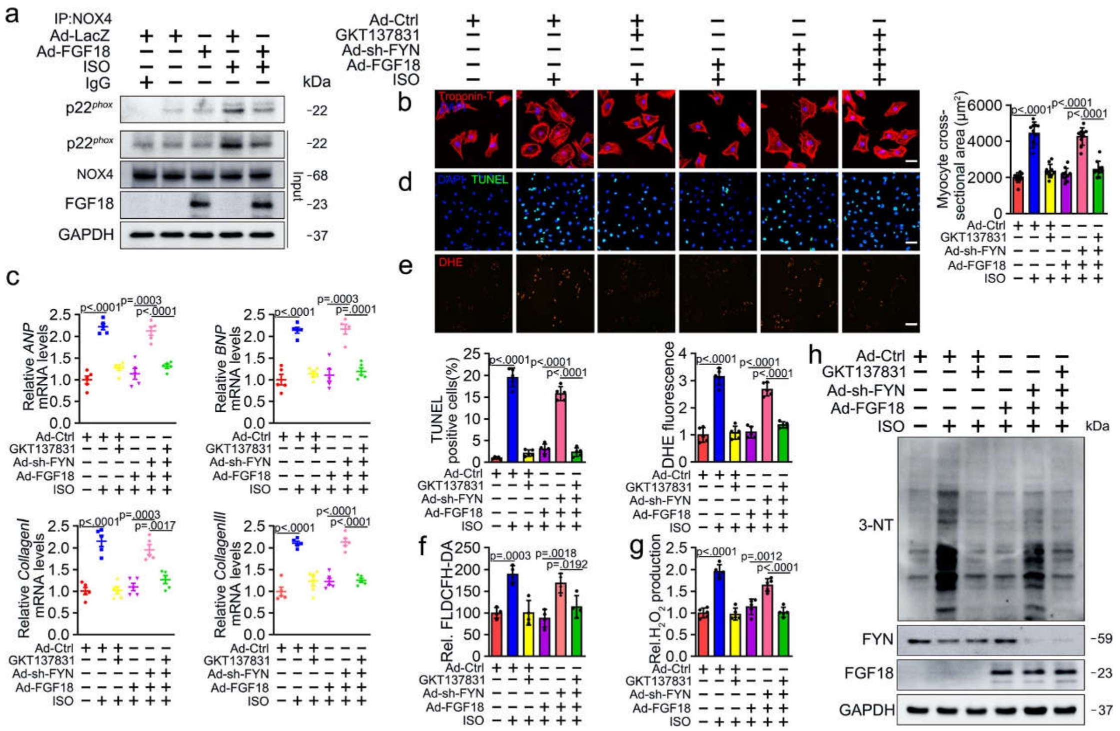


**Supplementary Fig. 18 FGF18 relieves cardiac hypertrophy by reducing the activity of NOX4. a** The cell lysates were subjected to immunoprecipitation with NOX4 antibody in NRCMs infected with Ad-LacZ or Ad-FGF18 in the presence or absence of ISO (10 μM) for 48 h, followed by immunoblotting with the p22*phox* antibody. n=4. IgG as the negative control. (**b-f**) NRCMs infected with Ad-LacZ or Ad-FGF18 and Ad-Scramble or Ad-sh-FYN were subjected to ISO (10 μM) in the presence or absence of GKT137631 for 48 h. **b** Representative images of NRCMs (blue: DAPI; red: c-Troponin-T; scale bar=35 μm). Quantification of cell surface area (right panel). n=10. **c** Real-time quantitative PCR assays. n=5. **d** The apoptotic cells (green: TUNEL$^+$ cells , blue: DAPI). Scale bars = 45 μm. The quantitative analysis of TUNEL$^+$ cells (right panel). n=5. Two-tailed Non-parametric Mann-Whitney U test was used. **e** Fluorescent images and quantitation of superoxide levels using DHE (down panel). Scale bars = 130 μm. n=5. Two-tailed Non-parametric Mann-Whitney U test. **f** Total ROS levels (by DCFH-DA probe) were quantified. n=4. **g** Hydrogen peroxide levels (by Amplex Red assay) quantified in different groups. n=5. One-way ANOVA followed by a post-hoc Fisher's comparison test. **h** Levels and quantitation of 3-NT in NRCMs. n=5. All quantitative data are reported as means ± SD, one-way ANOVA with Tukey multiple comparisons test. All numbers (n) are biologically independent experiments. Source data are provided as a Source Data file.

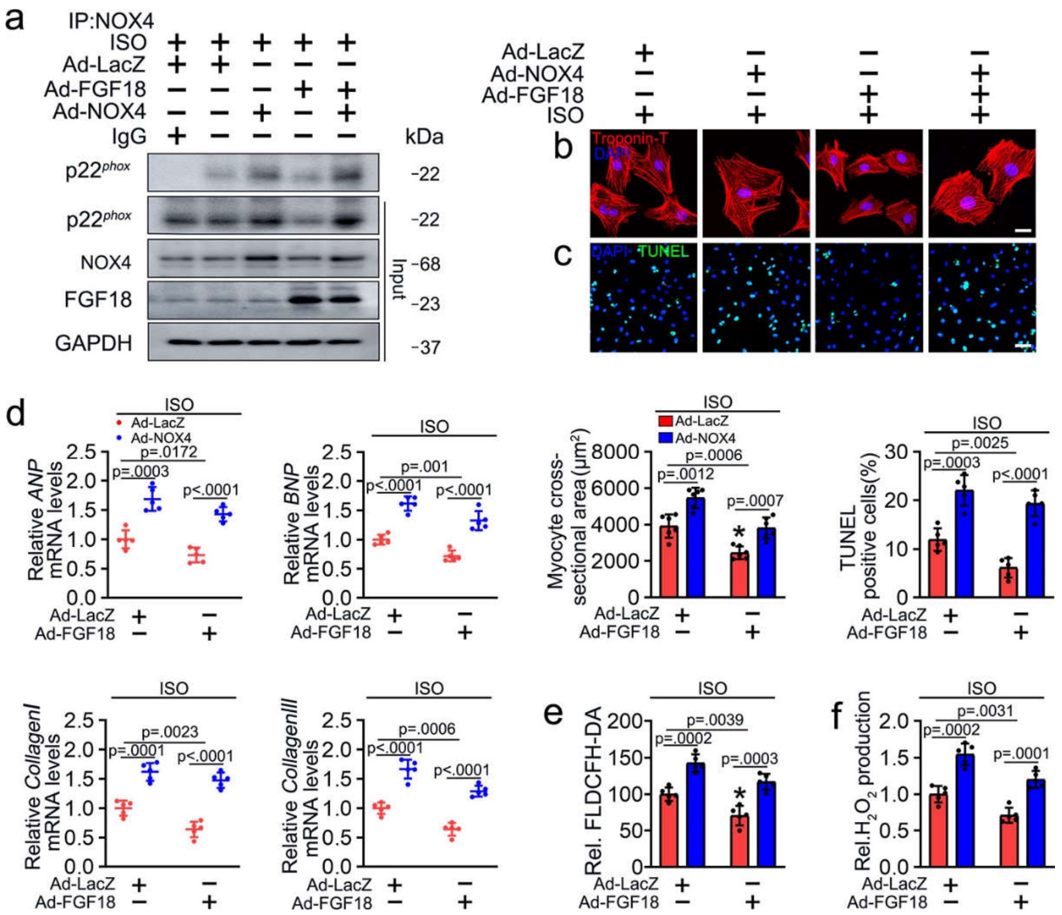


**Supplementary Fig. 19 NOX4 aggravates ISO-induced cardiomyocyte hypertrophy.** NRCMs were infected with Ad-LacZ, Ad-FGF18 and Ad-NOX4 in the presence or absence of ISO (10 μM) and for 48 h. **a** The cell lysates were subjected to immunoprecipitation with NOX4 antibody, followed by immunoblotting with the p22*phox* antibody. n=4. IgG as the negative control. **b** Representative images of NRCMs (blue: DAPI; red: c-Troponin-T; scale bar=35 μm). Quantification of cell surface area as shown in down panel, n=6. **c** The apoptotic cells (green: TUNEL$^+$ cells , blue: DAPI). Scale bars = 45 μm. The quantitative analysis of TUNEL$^+$ cells (down panel). n=5. Two-tailed Non-parametric Mann-Whitney U test was used. **d** Real-time PCR assays. n=5. **e** Total ROS levels (by DCFH-DA probe) were quantified. n=5. **f** Hydrogen peroxide levels (by Amplex Red assay) quantified in different groups. n=5. One-way ANOVA followed by a post-hoc Fisher's comparison test. All quantitative data are reported as means ± SD, one-way ANOVA with Tukey multiple comparisons test. All numbers (n) are biologically independent experiments. Source data are provided as a Source Data file.

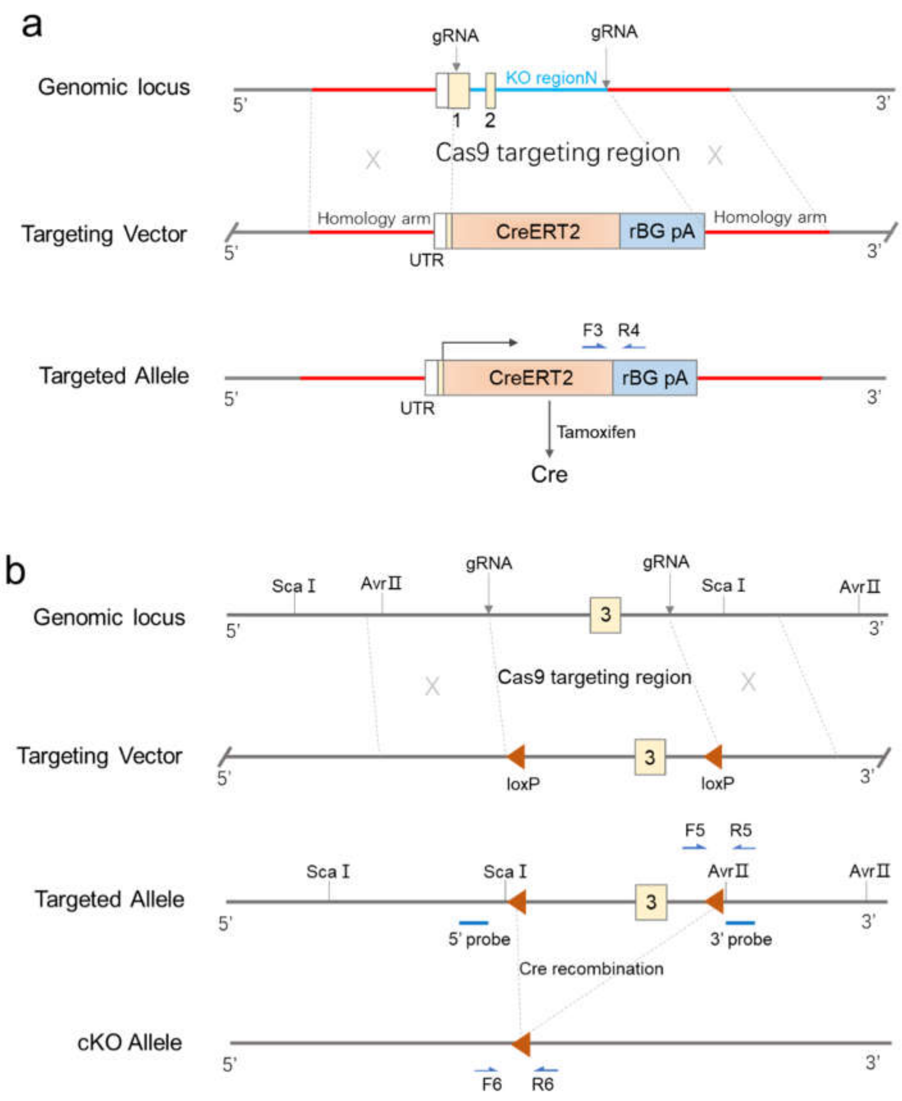


**Supplementary Fig. 20 Schematic illustration of the gene targeting strategy. a** To create FGF18 heterozygous (*Fgf18*$^{+/-}$KO) mice in C57BL/6J mice by CRISPR/Cas-mediated genome engineering. The region from coding sequence of exon 1 to partial intron 2 was replaced with CreERT2-rBG pA cassette. Cas9 and gRNA of targeting sequence will be co-injected into fertilized eggs with targeting vector for mice production. Arrows (F: forward primer, R: reverse prime) indicate PCR primers used for genotyping. **b** Schematic demonstrating the strategy for the generation of *Fgf18*-CKO mice. To generate *Fgf18* conditional knockout model by CRISPR/Cas-mediated genome editing, *Fgf18* Exon 3 was selected as conditional knockout region (cKO region). The gRNA to *Fgf18*, the donor vector containing loxP sites, and Cas9 mRNA were co-injected into fertilized mouse eggs to generate targeted conditional knockout offspring. F0 founder animals were identified by PCR followed by sequence analysis, which were bred to wildtype mice to test germline transmission and F1 animal generation. The correct gene targeting in F1 animals were confirmed by Southern blot analysis of the tail DNA samples. Arrows (F: forward primer, R: reverse prime) indicate PCR primers used for genotyping.

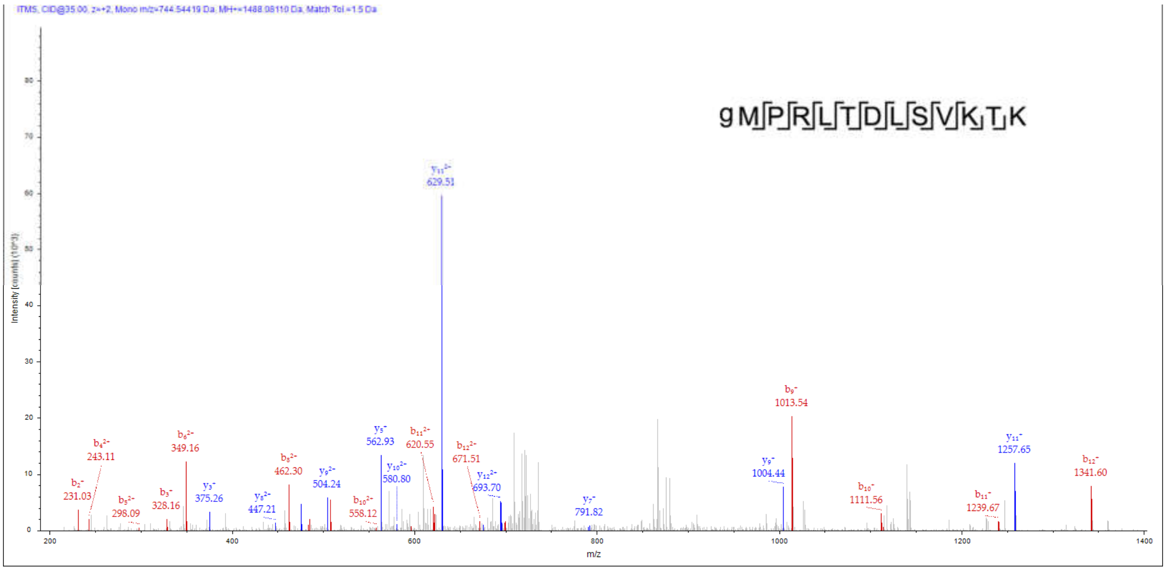


**Supplementary Fig. 21 Tandem mass spectra of representative identified FYN peptide.** NRCMs were treated with ISO (10 μM) in the presence or absence of FGF18 (50 ng/mL) for 48 h. Protein bands at ~60 kDa were excised from the SDS-PAGE gels for LC-MS/MS analysis. The fragment ions match the y and b ions of the peptides.

**Supplementary Table 1**: qRT-PCR primer sequences

| | Mouse | Rat |
|---|---|---|
| *FGF1* | GGGGAGATCACAACCTTCGC | GGGGAGATCACAACCTTTGC |
| | GTCCCTTGTCCCATCCACG | GTCCCTGGTCCCATCCACG |
| *FGF2* | GCGACCCACACGTCAAACTA | GCGACCCACACGTCAAACTA |
| | CCGTCCATCTTCCTTCATAGC | CCGTCCATCTTCCTTCATAGC |
| *FGF3* | TGCGCTACCAAGTACCACC | TGCGCTACCAAGTACCACC |
| | CACCGCAGTAATCTCCAGGAT | CACAGCAGTAATCTCCAGGAT |
| *FGF4* | TGGGCCTCAAAAGGCTTCG | TGGGCCTCAAAAGGCTGCG |
| | CGTCGGTAAAGAAAGGCACAC | CGTCGGTAAAGAAAGGCACAC |
| *FGF5* | GAAGCGTCTCACTCCCGAAG | GAAGCGTCTCACTCCCGAAG |
| | GAAGAAAACGTCGCGCTACT | GAAGCAAACGTCGCGCTACT |
| *FGF6* | CAGGCTCTCGTCTTCTTAGGC | CAGGCTCTCGTCTTCTTAGGC |
| | TTCACACCCGAAATCTCTCCA | TTCACACCCGAAATCTCTCCA |
| *FGF7* | TGGGCACTATATCTCTAGCTTGC | TGGGCACCATATCTTTAGCTTGC |
| | GGGTGCGACAGAACAGTCT | GGGTGCGACAGAACAGTCT |
| *FGF8* | AGAGCCTGGTGACGGATCA | AGAGCCTGGTGACGGATCA |
| | CTTCCAAAAGTATCGGTCTCCAC | CTTCCAAAAGTATCGGTCTCCAC |
| *FGF9* | ATGGCTCCCTTAGGTGAAGTT | ATGGCTCCCTTAGGTGAAGTT |
| | TCCGCCTGAGAATCCCCTTT | TCCGCCTGAGAATCCCCTTT |
| *FGF10* | TCAGCGGGACCAAGAATGAAG | GATACTGACACATTGTGCCTCAG |
| | CGGCAACAACTCCGATTTCC | CGGCAACAACTCCGATTTCC |
| *FGF11* | CCAGTAGCCTGATCCGACAGA | CCAGTAGCCTGATCCGACAGA |
| | GGCAGAACAGTTTGGTGACG | GGCAGAACAGTTTGGTGACG |
| *FGF12* | GTACCATTGATGGGACCAAGG | GTACCATTGATGGGACCAAGG |
| | CCACACGCAGTCCTACAGG | CCACACGCAGTCCCACAGG |
| *FGF13* | TCGCTCATCCGGCAAAAGAG | TCGCTTATCCGGCAAAAGAG |
| | TGTCGGCTGTATAGTTTGGTAAC | TGTCGGCTGTAGAGTTTGGTGAC |
| *FGF14* | CCCCAGCTCAAGGGCATAG | CCCCAGCTCAAGGGCATAG |
| | TGATGGGTAGAGGTAACCTTCTC | TGATGGGTAGAGGTAACCTTCTC |
| *FGF15* | ATGGCGAGAAAGTGGAACGG | GCGAGAAAGTGGAGTGGGCG |
| | GGACCAGCGGAGTACAGGT | CGTATATCTTGCCGTCGGCGC |
| *FGF16* | TGGATCGAAGAAACTCACACG | TGGATCGAAGAAACTCACACG |
| | CTCCGAGTCCGAGTGTTTGTA | CTCCGAGTCCGAGTGTTTGTA |
| *FGF17* | GTGCTTGCAGCTATTGATTCTCT | GTGCTTGCAGCTATTGATCCTCT |
| | GGTCCCTCACGTACTGGTTAAAA | GGTCCCTCACGTACTGGTTAAAA |
| *FGF18* | CTGCGCTTGTACCAGCTCTAT | GGGCTCGCGATGATGTGAG |
| | GACTCCCGAAGGTATCTGTCT | CGCTTCATGAAGTGCACATC |
| *FGF20* | AGGATCACAGTCTCTTCGGTATC | AGGATCACAGCCTCTTCGGTATC |
| | GTCATTCATCCCAAGGTACAGG | GCCATTCATGCCAAGGTACAGG |
| *FGF21* | GTGTCAAAGCCTCTAGGTTTCTT | GTGTCAAAGCCTCTAGGTTTCTT |
| | GGTACACATTGTAACCGTCCTC | GGTACACATTGTATCCGTCCTT |
| *FGF22* | CTCTGTGGACTGTAGGTTCCG | CTCTGTGGACTGTAGGTTCCG |
| | GAGGCGTATGTGTTGTAGCC | GAGGCGTATGTGTTGTAGCC |
| *FGF23* | ATGCTAGGGACCTGCCTTAGA | ATGCTGGGGGCCTGCCTCAGA |
| | GGAGCCAAGCAATGGGGAA | GGAGCCAAGCAGTGGGGAA |
| *ANP* | TGCTTCCTCAGTCTGCTC | ATCTGATGGATTTCAAGAACC |
| | CAACACAGATCTGATGGATTTCA | CTCTGAGACGGGTTGACTTC |
| *BNP* | GGGCTGTAACGCACTGAAGTT | TGATTCTGCTCCTGCTTTTC |
| | AGTTTGTGCTCCAAGATAAGA | GTGGATTGTTCTGGAGACTG |
| *Collagen I* | CCGCTGGTCAAGATGGTC | ACCTCAGGGTATTGCTGGAC |
| | CCTCGCTCTCCAGCCTTT | ACCTTGTTTGCCGGGTTCAC |
| *Collagen III* | GTGGCTTTTCACCCTATTAT | GCGGCTTTTCACCATATTAC |
| | GCATGTTTCCCCAGTTTC | GCATGTTTCTCCGGTTTC |
| *FYN* | ACCTCCATCCCGAACTACAAC | TTCCTAGCAACTACGTGGCTC |
| | CATAAAGCGCCACAAACAGTG | GCATCCTTGCGGCCAAGTTTT |
| *GAPDH* | ATGACAACTTTGTCAAGCTCATTT | ATGGGAAGCTGGTCATCAAC |
| | GGTCCACCACCCTGTTGCT | GTGGTTCACACCCATCACAA |

**Supplementary Table 2**. Effect of AAV9-FGF18 treatment on LV function evaluated by echocardiography at 6 weeks after Sham and TAC operation mice.

| | Sham | | TAC | |
|---|---|---|---|---|
| | AAV9-LacZ | AAV9-FGF18 | AAV9-LacZ | AAV9-FGF18 |
| HR (beats/min) | 489±20 | 478±35 | 468±25 | 481±22 |
| LVIDd (mm) | 3.78±0.07 | 3.66±0.12 | 4.11±0.12$^{\#.0017}$ | 3.87±0.13$^{*.0291}$ |
| LVIDs (mm) | 2.34±0.13 | 2.28±0.06 | 3.19±0.15$^{\#<.0001}$ | 2.60±0.11$^{*.0002}$ |
| LVEDV ($mm^3$) | 66.15±2.31 | 63.62±2.47 | 83.92±8.42$^{\#\circ\ 0036}$ | 70.45±2.75$^{*.0161}$ |
| LVESV ($mm^3$) | 26.00±2.40 | 24.87±1.18 | 49.72±3.05$^{\#<.0001}$ | 32.85±1.90$^{*<.0001}$ |
| LVPWTd (mm) | 0.68±0.07 | 0.67±0.06 | 0.90±0.06$^{\#.0015}$ | 0.75±0.06$^{*.0080}$ |
| LVPWTs (mm) | 0.87±0.04 | 0.88±0.04 | 1.25±0.04$^{\#<.0001}$ | 0.99±0.03$^{*<.0001}$ |
| LVM (mg) | 99.17±8.68 | 99.21±6.36 | 140.07±9.01$^{\#.0002}$ | 110.06±5.00$^{*.0004}$ |
| LVFS (%) | 38.14±2.88 | 37.57±1.58 | 22.23±2.59$^{\#<.0001}$ | 32.92±3.30$^{*.0009}$ |
| LVEF (%) | 60.66±3.67 | 61.01±2.40 | 41.36±4.69$^{\#.0001}$ | 53.21±4.40$^{*.004}$ |

HR, heart rate; LVIDd, LV internal diameter during diastole; LVIDs, LV internal diameter during systole; EDV, end-diastolic volume; ESV, end-systolic volume; LVPWTd, posterior wall thickness during diastole; LVPWTs, posterior wall thickness during systole; LVM, LV mass; LVEF, LV ejection fraction; LVFS, LV fractional shortening; All measurements are means ± SD. Data were analysed by one-way ANOVA (#p versus Sham/AAV9-LacZ, *p versus TAC/AAV9-LacZ).

**Supplementary Table 3** Effect of AAV9-sh-FGFR3 treatment on LV function evaluated by echocardiography at 6 weeks after Sham and TAC operation mice.

| | Sham | | TAC | | |
|---|---|---|---|---|---|
| | AAV9-Ctrl | AAV9-shFGFR3 | AAV9-Ctrl | AAV9-FGF18 | AAV9-FGF18+shFGFR3 |
| HR (beats/min) | 462±25 | 464±21 | 471±15 | 453±25 | 464±17 |
| LVIDd (mm) | 3.68±0.08 | 3.71±0.09 | 4.11±0.12$^{\#.0003}$ | 3.86±0.15$^{*.0326}$ | 4.10±0.11$^{\&.0328}$ |
| LVIDs (mm) | 2.22±0.05 | 2.25±0.08 | 3.01±0.11$^{\#<001}$ | 2.50±0.14$^{*.0005}$ | 3.05±0.09$^{\&.0002}$ |
| LVEDV ($mm^3$) | 61.35±2.28 | 60.65±3.21 | 84.02±3.46$^{\#<.0001}$ | 73.48±2.89$^{*.0016}$ | 82.47±2.76$^{\&.002}$ |
| LVESV ($mm^3$) | 23.35±2.45 | 22.58±2.64 | 46.54±2.68$^{\#<.0001}$ | 32.25±1.48$^{*<.0001}$ | 43.37±2.48$^{\&<.0001}$ |
| LVPWTd (mm) | 0.63±0.05 | 0.64±0.08 | 1.10±0.09$^{\#<.0001}$ | 0.81±0.09$^{*.0002}$ | 0.99±0.09$^{\&.0232}$ |
| LVPWTs (mm) | 0.87±0.06 | 0.85±0.04 | 1.29±0.10$^{\#<.0001}$ | 0.92±0.08$^{*.0003}$ | 1.22±0.08$^{\&.0005}$ |
| LVM (mg) | 90.25±2.68 | 89.27±2.48 | 139.37±8.85$^{\#<.0001}$ | 112.84±3.75$^{*.0006}$ | 133.71±5.68$^{\&.0003}$ |
| LVFS (%) | 39.65±2.48 | 38.97±3.46 | 26.57±4.69$^{\#.0012}$ | 35.18±4.65$^{*.0313}$ | 25.49±3.14$^{\&.0087}$ |
| LVEF (%) | 61.96±3.76 | 62.97±3.95 | 44.56±2.87$^{\#<.0001}$ | 56.00±3.41$^{*.0009}$ | 47.38±2.95$^{\&.0051}$ |

HR, heart rate; LVIDd, LV internal diameter during diastole; LVIDs, LV internal diameter during systole; EDV, end-diastolic volume; ESV, end-systolic volume; LVPWTd, LV posterior wall thickness during diastole; LVPWTs, LV posterior wall thickness during systole; LVM, LV mass; LVEF, LV ejection fraction; LVFS, LV fractional shortening; All measurements are means ± SD. Data were analysed by one-way ANOVA (#p versus Sham/AAV9-Ctrl, *p versus TAC/AAV9-Ctrl, &p versus TAC/AAV9-FGF18).

**Supplementary Table 4** Echocardiographic analysis in FGF18 wild-type (WT) and FGF18 heterozygous mice (*Fgf18*$^{+/-}$KO) mice subjected to Sham or TAC operation for 6 weeks.

| | Sham | | TAC | |
|---|---|---|---|---|
| | WT | *Fgf18*$^{+/-}$KO | WT | *Fgf18*$^{+/-}$KO |
| HR (beats/min) | 493±25 | 489±35 | 497±38 | 485±30 |
| LVIDd (mm) | 3.75±0.13 | 3.71±0.09$^{n.s.}$ | 3.97±0.15$^{\#.0195}$ | 4.21±0.09$^{*.0063}$ |
| LVIDs (mm) | 2.32±0.11 | 2.29±0.08$^{n.s.}$ | 3.04±0.10$^{\#<.0001}$ | 3.48±0.05$^{*<.0001}$ |
| LVEDV ($mm^3$) | 63.22±2.44 | 62.78±2.32$^{n.s.}$ | 86.72±5.53$^{\#<.0001}$ | 94.51±3.59$^{*.0135}$ |
| LVESV ($mm^3$) | 25.01±2.12 | 24.35±2.19$^{n.s.}$ | 49.81±2.97$^{\#<.0001}$ | 59.34±4.87$^{*\circ\ 0015}$ |
| LVPWTd (mm) | 0.61±0.07 | 0.63±0.08$^{n.s.}$ | 1.05±0.08$^{\#<.0001}$ | 1.15±0.14$^{.1536}$ |
| LVPWTs (mm) | 0.86±0.04 | 0.85±0.03$^{n.s.}$ | 1.32±0.09$^{\#<.0001}$ | 1.49±0.07$^{*.0035}$ |
| LVM (mg) | 96.01±3.67 | 95.39±4.24$^{n.s.}$ | 141.25±7.97$^{\#<.0001}$ | 165.25±9.34$^{*.0004}$ |
| LVFS (%) | 38.27±1.45 | 38.21±3.21$^{n.s.}$ | 23.32±4.54$^{\#.0003}$ | 17.41±2.43$^{*.0156}$ |
| LVEF (%) | 60.31±4.43 | 61.12±4.35$^{n.s.}$ | 42.37±4.13$^{\#<.0001}$ | 37.29±3.21$^{*.0346}$ |

HR, heart rate; LVIDd, LV internal diameter during diastole; LVIDs, LV internal diameter during systole; EDV, end-diastolic volume; ESV, end-systolic volume; LVPWTd, LV posterior wall thickness during diastole; LVPWTs, LV posterior wall thickness during systole; LVM, LV mass; LVEF, LV ejection fraction; LVFS, LV fractional shortening; All measurements are means ± SD. Data were analysed by one-way ANOVA (n.s. versus WT/Sham, #p versus WT/Sham, *p versus WT/TAC).

**Supplementary Table 5** Echocardiographic analysis in α-MHC-Cre, FGF18-Flox and *Fgf18*-CKO mice subjected to Sham or TAC operation for 6 weeks.

| | Sham | | TAC | |
|---|---|---|---|---|
| | WT | *Fgf18*-CKO | WT | *Fgf18*-CKO |
| HR (beats/min) | 466±26 | 478±36 | 459±31 | 482±29 |
| LVIDd (mm) | 3.67±0.11 | 3.63±0.08$^{n.s.}$ | 3.99±0.11$^{\#.0032}$ | 4.18±0.10$^{*.0395}$ |
| LVIDs (mm) | 2.22±0.09 | 2.18±0.10$^{n.s.}$ | 3.10±0.13$^{\#<.0001}$ | 3.46±0.04$^{*.0007}$ |
| LVEDV ($mm^3$) | 64.41±2.54 | 63.57±2.24$^{n.s.}$ | 87.81±5.75$^{\#<.0001}$ | 93.01±3.70$^{*.1675}$ |
| LVESV ($mm^3$) | 24.95±2.22 | 24.47±2.09$^{n.s.}$ | 49.93±0.96$^{\#<.0001}$ | 58.28±2.66$^{*.0004}$ |
| LVPWTd (mm) | 0.66±0.09 | 0.68±0.06$^{n.s.}$ | 1.05±0.06$^{\#<.0001}$ | 1.11±0.10$^{*.3263}$ |
| LVPWTs (mm) | 0.87±0.03 | 0.87±0.02$^{n.s.}$ | 1.32±0.08$^{\#<.0001}$ | 1.40±0.08$^{*\ .2261}$ |
| LVM (mg) | 94.11±3.58 | 92.39±6.77$^{n.s.}$ | 144.84±8.97$^{\#<.0001}$ | 163.14±8.77$^{*.0194}$ |
| LVFS (%) | 39.57±1.72 | 39.91±3.14$^{n.s.}$ | 22.54±3.21$^{\#<.0001}$ | 17.09±2.98$^{*.0376}$ |
| LVEF (%) | 61.23±3.53 | 61.37±4.48$^{n.s.}$ | 42.19±3.65$^{\#<.0001}$ | 37.93±2.70$^{*.0385}$ |

HR, heart rate; LVIDd, LV internal diameter during diastole; LVIDs, LV internal diameter during systole; EDV, end-diastolic volume; ESV, end-systolic volume; LVPWTd, LV posterior wall thickness during diastole; LVPWTs, LV posterior wall thickness during systole; LVM, LV mass; LVEF, LV ejection fraction; LVFS, LV fractional shortening; All measurements are means ± SD. Data were analysed by one-way ANOVA (n.s. versus WT/Sham, #p versus WT/Sham, *p versus WT/TAC).

**Supplementary Table 6** FYN-deficient mice have normal cardiac function at the basal level.

| | Sham | |
|---|---|---|
| | AAV9-Scramble | AAV9-sh-FYN |
| HR (beats/min) | 455±13 | 449±25$^{n.s.}$ |
| LVIDd (mm) | 3.75±0.12 | 3.64±0.09$^{n.s.}$ |
| LVIDs (mm) | 2.13±0.07 | 2.09±0.14$^{n.s.}$ |
| LVEDV ($mm^3$) | 60.12±4.34 | 63.78±3.69$^{n.s.}$ |
| LVESV ($mm^3$) | 21.64±3.24 | 19.84±4.46$^{n.s.}$ |
| LVPWTd (mm) | 0.61±0.07 | 0.59±0.05$^{n.s.}$ |
| LVPWTs (mm) | 0.82±0.06 | 0.81±0.07$^{n.s.}$ |
| LVM (mg) | 88.81±3.68 | 91.36±5.41$^{n.s.}$ |
| LVFS (%) | 39.25±3.15 | 38.21±3.64$^{n.s.}$ |
| LVEF (%) | 63.44±2.62 | 62.42±2.84$^{n.s.}$ |

HR, heart rate; LVIDd, LV internal diameter during diastole; LVIDs, LV internal diameter during systole; EDV, end-diastolic volume; ESV, end-systolic volume; LVPWTd, LV posterior wall thickness during diastole; LVPWTs, LV posterior wall thickness during systole; LVM, LV mass; LVEF, LV ejection fraction; LVFS, LV fractional shortening; All measurements are means ± SD. Data were analysed by two-tailed student's t-test. n.s. = not significant.

**Supplementary Table 7** Effect of AAV9-sh-FYN treatment on LV function evaluated by echocardiography at 6 weeks after Sham and TAC operation mice.

| | Sham | TAC | | |
|---|---|---|---|---|
| | AAV9-Ctrl | AAV9-Ctrl | AAV9-FGF18 | AAV9-FGF18+shFYN |
| HR (beats/min) | 478±33 | 469±24 | 486±35 | 472±26 |
| LVIDd (mm) | 3.73±0.12 | 4.10±0.11$^{\#.0019}$ | 3.84±0.17$^{*.0361}$ | 4.03±0.12$^{\&.0114}$ |
| LVIDs (mm) | 2.29±0.05 | 3.15±0.09$^{\#<.0001}$ | 2.50±0.15$^{*<.0001}$ | 2.99±0.09$^{\&.0005}$ |
| LVEDV ($mm^3$) | 64.80±2.47 | 82.32±2.30$^{\#<.0001}$ | 71.36±3.57$^{*.0009}$ | 80.26±3.56$^{\&.0077}$ |
| LVESV ($mm^3$) | 24.09±1.27 | 47.45±1.51$^{\#<.0001}$ | 32.60±1.56$^{*<.0001}$ | 43.69±3.09$^{\&.0002}$ |
| LVPWTd (mm) | 0.61±0.04 | 1.02±0.09$^{\#<.0001}$ | 0.77±0.06$^{*0022}$ | 0.98±0.05$^{\&.001}$ |
| LVPWTs (mm) | 0.88±0.04 | 1.30±0.08$^{\#<.0001}$ | 0.96±0.03$^{*<.0001}$ | 1.21±0.07$^{\&.0002}$ |
| LVM (mg) | 91.73±3.25 | 145.41±9.07$^{\#<.0001}$ | 110.23±5.98$^{*.0002}$ | 132.39±6.49$^{\&.001}$ |
| LVFS (%) | 38.35±2.03 | 23.15±3.76$^{\#.0001}$ | 34.97±4.91$^{*.0051}$ | 25.69±2.20$^{\&.0086}$ |
| LVEF (%) | 62.81±1.79 | 42.35±0.77$^{\#<.0001}$ | 54.22±2.39$^{*<.0001}$ | 45.60±2.06$^{\&.0006}$ |

HR, heart rate; LVIDd, LV internal diameter during diastole; LVIDs, LV internal diameter during systole; EDV, end-diastolic volume; ESV, end-systolic volume; LVPWTd, LV posterior wall thickness during diastole; LVPWTs, LV posterior wall thickness during systole; LVM, LV mass; LVEF, LV ejection fraction; LVFS, LV fractional shortening; All measurements are means ± SD. Data were analysed by one-way ANOVA (#p versus Sham/AAV9-Ctrl, *p versus TAC/AAV9-Ctrl, &p versus TAC/AAV9-FGF18).

**Supplementary Table 8** Effect of AAV9-sh-FYN treatment on LV function evaluated by echocardiography at 6 weeks after TAC operation mice.

| | TAC | | | |
|---|---|---|---|---|
| | AAV9-Scramble | | AAV9-sh-FYN | |
| | AAV9-LacZ | AAV9-FGF18 | AAV9-LacZ | AAV9-FGF18 |
| HR (beats/min) | 459±14 | 458±15 | 452±17 | 471±20 |
| LVIDd (mm) | 3.97±0.08 | 3.76±0.11$^{\#.0134}$ | 4.23±0.15 | 4.04±0.11$^{*0067}$ |
| LVIDs (mm) | 2.78±0.09 | 2.33±0.09$^{\#.0001}$ | 3.16±0.06 | 2.81±0.12$^{*.0002}$ |
| LVEDV ($mm^3$) | 87.25±4.24 | 69.64±1.34$^{\#<.0001}$ | 103.72±5.62 | 85.64±3.13$^{*<.0001}$ |
| LVESV ($mm^3$) | 49.85±3.21 | 26.75±2.64$^{\#<.0001}$ | 66.81±4.04 | 44.34±3.71$^{*<.0001}$ |
| LVPWTd (mm) | 1.02±0.04 | 0.68±0.07$^{\#<.0001}$ | 1.18±0.09 | 0.98±0.08$^{*.0005}$ |
| LVPWTs (mm) | 1.22±0.10 | 0.91±0.08$^{\#.0015}$ | 1.42±0.07 | 1.19±0.09$^{*.0018}$ |
| LVM (mg) | 142.78±6.24 | 116.35±5.64$^{\#.0002}$ | 159.17±8.68 | 136.95±6.81$^{*.0016}$ |
| LVFS (%) | 30.18±1.97 | 37.84±2.26$^{\#.0009}$ | 25.21±3.25 | 30.61±2.34$^{*.0021}$ |
| LVEF (%) | 43.94±3.58 | 61.54±4.19$^{\#.0002}$ | 35.57±2.83 | 48.27±3.37$^{*.0011}$ |

HR, heart rate; LVIDd, LV internal diameter during diastole; LVIDs, LV internal diameter during systole; EDV, end-diastolic volume; ESV, end-systolic volume; LVPWTd, LV posterior wall thickness during diastole; LVPWTs, LV posterior wall thickness during systole; LVM, LV mass; LVEF, LV ejection fraction; LVFS, LV fractional shortening; All measurements are means ± SD. Data were analysed by one-way ANOVA (#p versus TAC/AAV9-LacZ, *p versus TAC/Scramble/AAV9-FGF18).

**Supplementary Table 9** Effect of AAV9-FYN treatment on LV function evaluated by echocardiography at 6 weeks after TAC operation *Fgf18*-CKO mice.

| | TAC | | | |
|---|---|---|---|---|
| | WT | | *Fgf18*-CKO | |
| | AAV9-LacZ | AAV9-FYN | AAV9-LacZ | AAV9-FYN |
| HR (beats/min) | 442±21 | 451±16 | 449±22 | 439±17 |
| LVIDd (mm) | 4.01±0.11 | 3.62±0.09$^{\#.0006}$ | 4.14±0.08 | 3.78±0.11$^{*.0009}$ |
| LVIDs (mm) | 2.86±0.12 | 2.18±0.08$^{\#<.0001}$ | 3.31±0.09 | 2.31±0.05$^{*<.0001}$ |
| LVEDV ($mm^3$) | 85.73±3.94 | 61.09±2.79$^{\#<.0001}$ | 92.73±3.25 | 75.46±4.08$^{*.0002}$ |
| LVESV ($mm^3$) | 48.49±3.01 | 27.95±2.75$^{\#<.0001}$ | 58.73±1.06 | 36.76±2.15$^{*<.0001}$ |
| LVPWTd (mm) | 0.91±0.11 | 0.62±0.09$^{\#.0036}$ | 1.12±0.09 | 0.85±0.11$^{*.0051}$ |
| LVPWTs (mm) | 1.21±0.04 | 0.88±0.05$^{\#<.0001}$ | 1.39±0.05 | 1.12±0.06$^{*.0001}$ |
| LVM (mg) | 132.67±5.34 | 115.67±4.39$^{\#.0012}$ | 149.36±6.84 | 133.14±4.34$^{*.0039}$ |
| LVFS (%) | 28.76±2.43 | 39.71±2.87$^{\#.0004}$ | 19.96±3.73 | 38.78±2.38$^{*<.0001}$ |
| LVEF (%) | 43.31±3.94 | 54.21±4.25$^{\#.0056}$ | 36.58±2.44 | 51.23±2.81$^{*<.0001}$ |

HR, heart rate; LVIDd, LV internal diameter during diastole; LVIDs, LV internal diameter during systole; EDV, end-diastolic volume; ESV, end-systolic volume; LVPWTd, LV posterior wall thickness during diastole; LVPWTs, LV posterior wall thickness during systole; LVM, LV mass; LVEF, LV ejection fraction; LVFS, LV fractional shortening; All measurements are means ± SD. Data were analysed by one-way ANOVA (#p versus WT/AAV9-LacZ, *p versus CKO/AAV9-LacZ.).

**Supplementary Table 10** Effect of AAV9-FYN$_{1\text{-}80}$ treatment on LV function evaluated by echocardiography at 6 weeks after Sham and TAC operation mice.

| | Sham | | | | TAC | | | |
|---|---|---|---|---|---|---|---|---|
| | AAV9-LacZ | | AAV9-FYN$_{1\text{-}80}$ | | AAV9-LacZ | | AAV9-FYN$_{1\text{-}80}$ | |
| | - | AAV9-FGF18 | - | AAV9-FGF18 | - | AAV9-FGF18 | - | AAV9-FGF18 |
| HR (beats/min) | 475±13 | 465±21 | 476±16 | 471±13 | 461±15 | 469±22 | 471±23 | 467±26 |
| LVIDd (mm) | 3.67±0.12 | 3.69±0.13 | 3.72±0.10 | 3.73±0.09 | 4.05±0.11$^{\#.0019}$ | 3.87±0.10$^{\&.0540}$ | 4.19±0.13$^{*.1282}$ | 3.98±0.12$^{\%.2466}$ |
| LVIDs (mm) | 2.21±0.07 | 2.19±0.10 | 2.26±0.08 | 2.19±0.11 | 2.81±0.07$^{\#<.0001}$ | 2.44±0.08$^{\&<.0001}$ | 3.41±0.15$^{*.0001}$ | 2.68±0.09$^{\%.0005}$ |
| LVEDV (mm$^3$) | 61.23±2.61 | 63.57±3.14 | 59.82±3.64 | 62.01±3.46 | 87.12±3.87$^{\#<.0001}$ | 72.35±3.45$^{\&.0005}$ | 79.85±4.62$^{*.0424}$ | 70.57±2.35$^{\%.4192}$ |
| LVESV (mm$^3$) | 23.12±2.84 | 24.34±1.64 | 24.65±2.35 | 23.84±2.65 | 49.83±2.48$^{\#<.0001}$ | 28.95±3.86$^{\&<.0001}$ | 51.75±3.48$^{*.3961}$ | 37.56±2.37$^{\%.0099}$ |
| LVPWTd(mm) | 0.62±0.11 | 0.58±0.08 | 0.61±0.10 | 0.62±0.09 | 1.10±0.08$^{\#<.0001}$ | 0.68±0.12$^{\&.0004}$ | 1.26±0.09$^{*.0322}$ | 1.04±0.09$^{\%.0015}$ |
| LVPWTs(mm) | 0.84±0.10 | 0.86±0.09 | 0.91±0.08 | 0.98±0.10 | 1.26±0.12$^{\#.0007}$ | 0.98±0.10$^{\&.0072}$ | 1.44±0.14$^{*.0885}$ | 1.12±0.16$^{\%.1799}$ |
| LVM (mg) | 88.24±4.56 | 90.34±4.51 | 88.86±4.38 | 89.37±4.64 | 128.68±5.67$^{\#<.0001}$ | 99.58±6.67$^{\&.0002}$ | 154.94±6.98$^{*.0004}$ | 118.68±4.24$^{\%.0013}$ |
| LVFS (%) | 39.54±3.54 | 40.35±3.76 | 38.92±3.34 | 41.10±3.54 | 30.36±2.91$^{\#.0039}$ | 37.10±2.69$^{\&.0093}$ | 18.74±3.54$^{*.0001}$ | 32.65±2.74$^{\%.0489}$ |
| LVEF (%) | 62.46±3.15 | 61.68±3.15 | 68.84±2.34 | 61.28±2.68 | 42.69±3.57$^{\#<.0001}$ | 58.79±4.68$^{\&.0006}$ | 35.05±4.68$^{*.0319}$ | 46.79±2.47$^{\%.0021}$ |

HR, heart rate; LVIDd, LV internal diameter during diastole; LVIDs, LV internal diameter during systole; EDV, end-diastolic volume; ESV, end-systolic volume; LVPWTd, LV posterior wall thickness during diastole; LVPWTs, LV posterior wall thickness during systole; LVM, LV mass; LVEF, LV ejection fraction; LVFS, LV fractional shortening; All measurements are means ± SD. Data were analysed by one-way ANOVA (#p versus Sham/AAV9-LacZ, *p versus TAC/AAV9-LacZ, &p versus TAC/AAV9-LacZ, %p versus TAC/AAV9-FGF18).

Online Figure 1

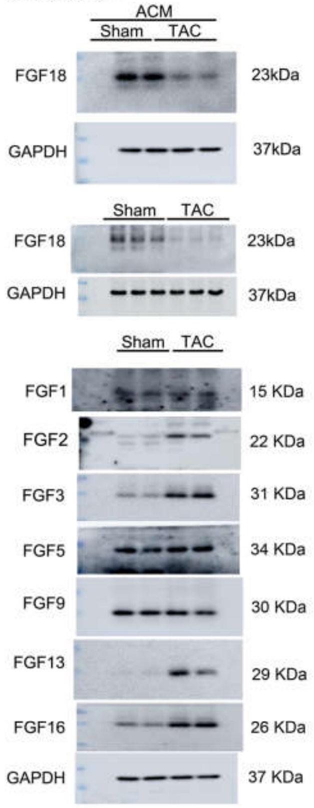


Online Figure 2

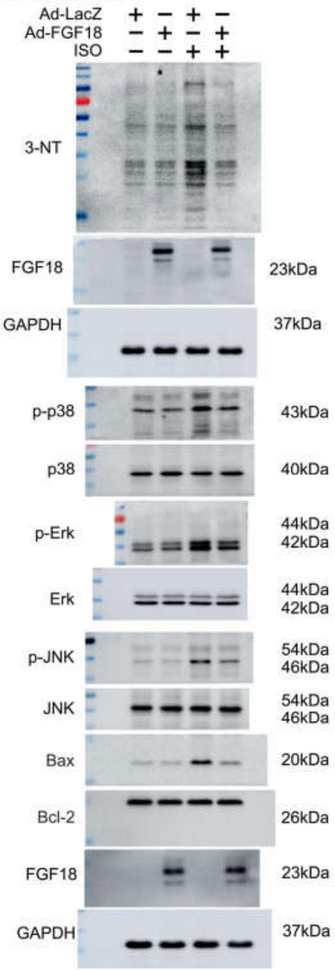


Online Figure 3

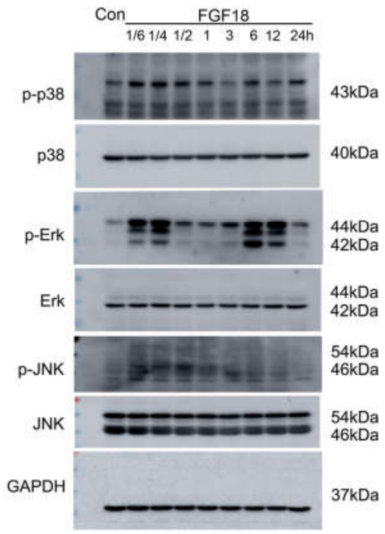

Online Figure 5

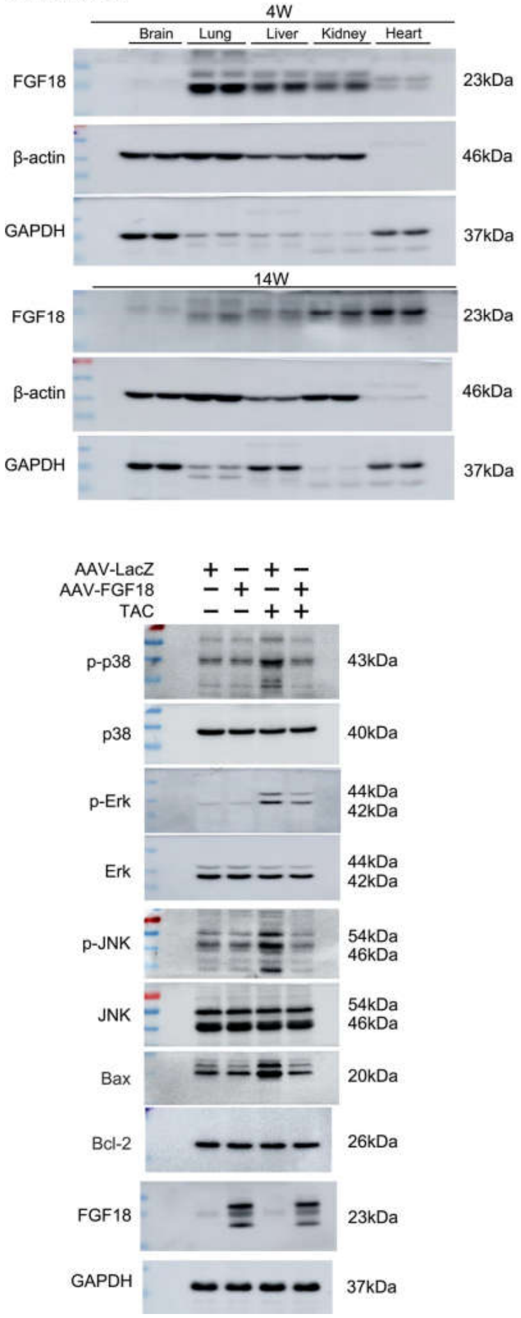


Online Figure 9

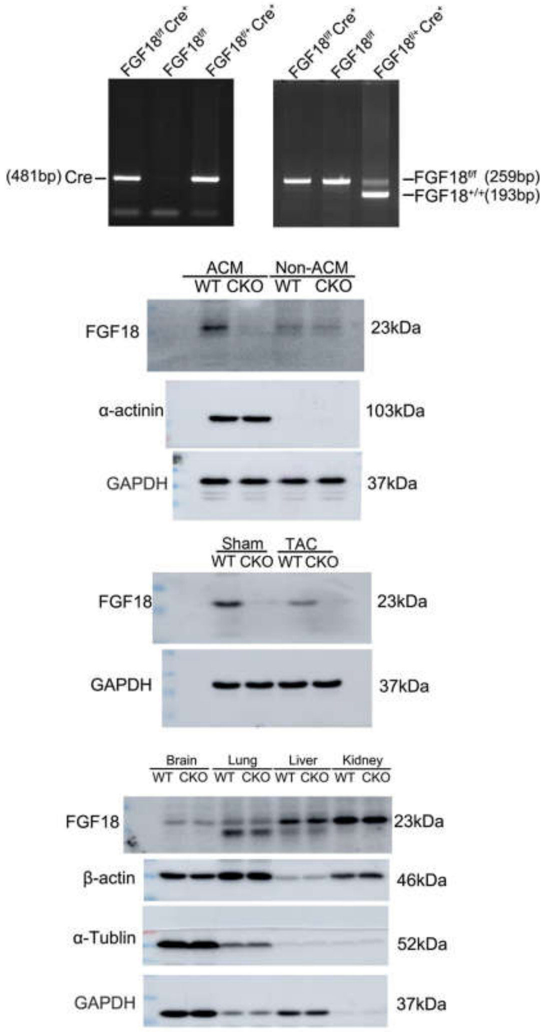

Online Figure 10

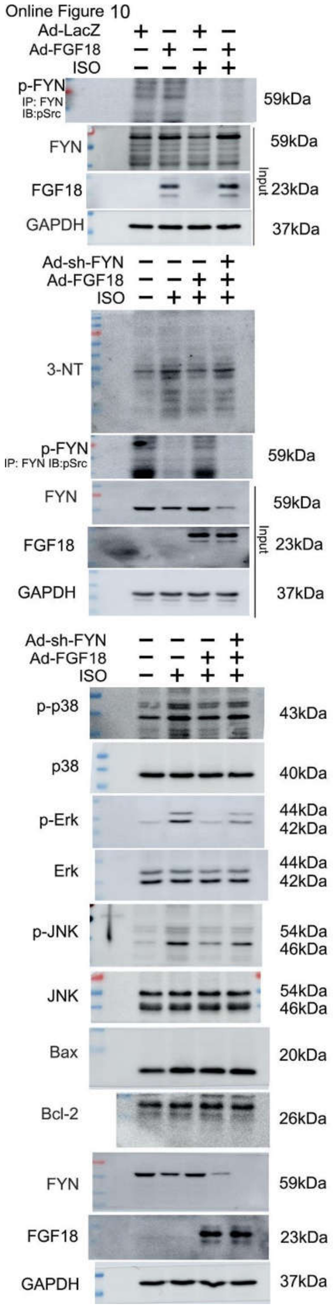


Online Figure 11

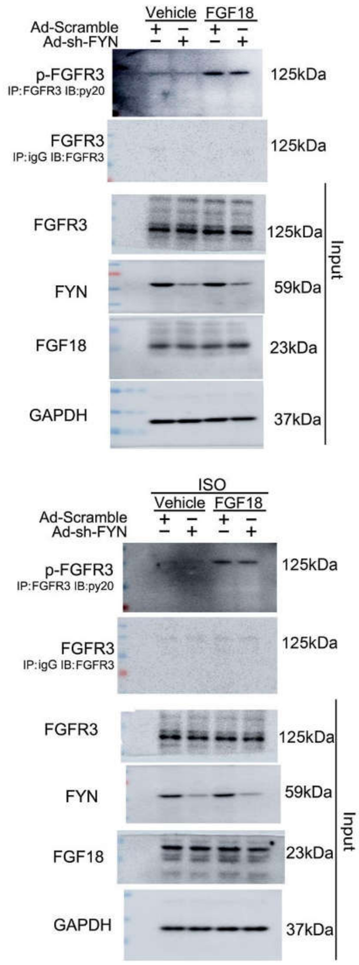


Online Figure 13

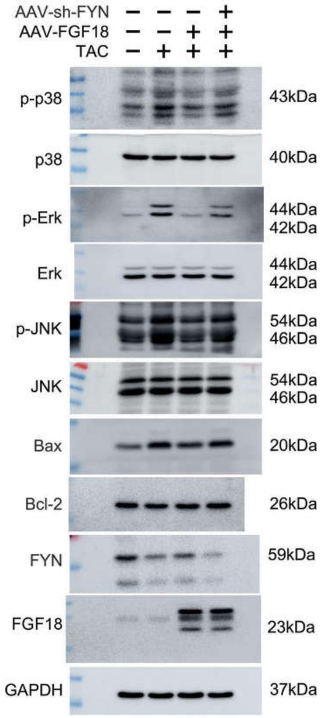

Online Figure15

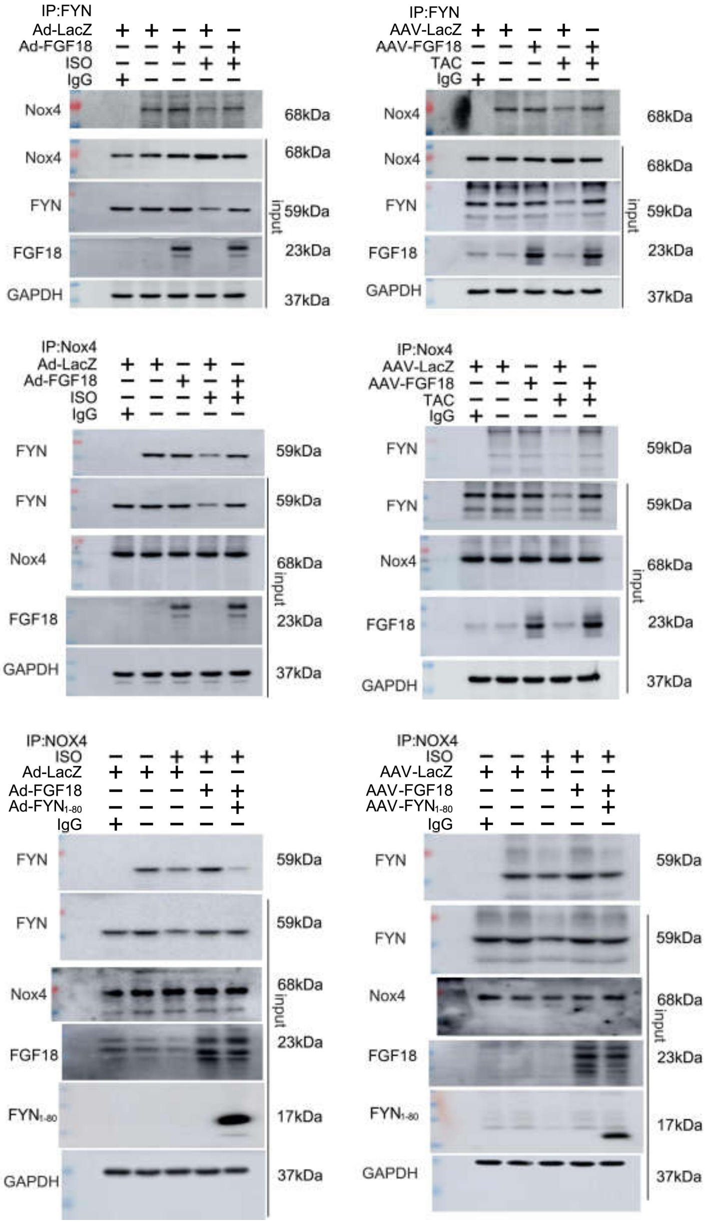


Online Figure 18

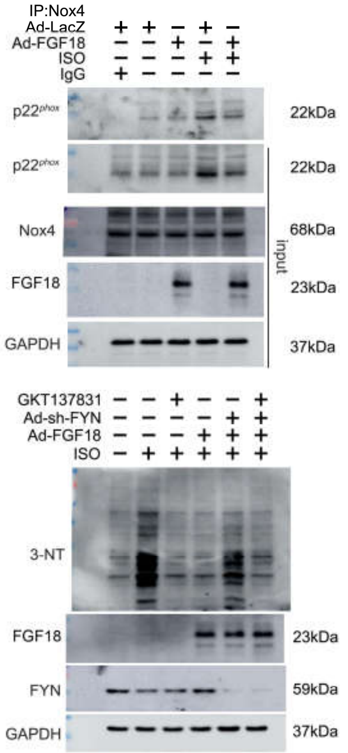


Online Figure 19

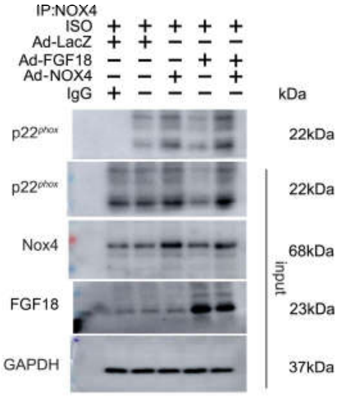

Supplement: Supplementary file 1 — Supplementary Information [file 41467_2023_36895_MOESM1_ESM.pdf]
