# Supplementary material for: Fibroblast growth factor 18 alleviates stress-induced pathological cardiac hypertrophy in male mice
Source: Nat Commun. 2023 Mar 4;14:1235. doi: 10.1038/s41467-023-36895-1 (PMC9985628; doi:10.1038/s41467-023-36895-1)
Supplement: Supplementary file 2 — Reporting Summary [file 41467_2023_36895_MOESM2_ESM.pdf]

nature portfolio

Corresponding author(s): Xu Wang; Weitao Cong; Xiaokun Li

Last updated by author(s): Feb 15, 2023

Reporting Summary

Nature Portfolio wishes to improve the reproducibility of the work that we publish. This form provides structure for consistency and transparency in reporting. For further information on Nature Portfolio policies, see our Editorial Policies and the Editorial Policy Checklist.

Statistics

For all statistical analyses, confirm that the following items are present in the figure legend, table legend, main text, or Methods section.

n/a	Confirmed	
☐	☒	The exact sample size (*n*) for each experimental group/condition, given as a discrete number and unit of measurement
☐	☒	A statement on whether measurements were taken from distinct samples or whether the same sample was measured repeatedly
☐	☒	The statistical test(s) used AND whether they are one- or two-sided<br>*Only common tests should be described solely by name; describe more complex techniques in the Methods section.*
☒	☐	A description of all covariates tested
☒	☐	A description of any assumptions or corrections, such as tests of normality and adjustment for multiple comparisons
☐	☒	A full description of the statistical parameters including central tendency (e.g. means) or other basic estimates (e.g. regression coefficient) AND variation (e.g. standard deviation) or associated estimates of uncertainty (e.g. confidence intervals)
☐	☒	For null hypothesis testing, the test statistic (e.g. *F*, *t*, *r*) with confidence intervals, effect sizes, degrees of freedom and *P* value noted<br>*Give P values as exact values whenever suitable.*
☒	☐	For Bayesian analysis, information on the choice of priors and Markov chain Monte Carlo settings
☒	☐	For hierarchical and complex designs, identification of the appropriate level for tests and full reporting of outcomes
☒	☐	Estimates of effect sizes (e.g. Cohen's *d*, Pearson's *r*), indicating how they were calculated

Our web collection on statistics for biologists contains articles on many of the points above.

Software and code

Policy information about availability of computer code

Data collection	Bound antibody was visualized using Pierce ECL plus western blotting substrate (Thermo Fisher Scientific, 32132). The protein bands were visualized by exposure machine (GE, Amersham 154 Imager 680).<br>Echocardiograms were obtained using a Vevo 1100 Ultrasound System (VisualSonics, Toronto, Canada) equipped with a high-frequency (30 MHz) linear array transducer.<br>Images were captured with a confocal laser scanning microscope (Leica TCS SP8, Wetzlar, Germany).<br>qRT-PCR analysis was performed on a QuantStudio™ 3 Real-Time PCR Detection System using ChamQ Universal SYBR qPCR Master Mix (Vazyme, Q711-02) with specific primers.<br>The eluted peptides were ionized and introduced into a Thermo Fisher LTQ Velos Pro mass spectrometer (Thermo Fisher Scientific, Bremen, Germany) using a Proxeon nanoelectrospray ion source.
Data analysis	The protein bands were quantified using Image Quant 5.2 software (Molecular Dynamics, Sunnyvale, CA).<br>Echocardiograms analysis was performed off-line on a workstation installed with Vevo LAB software (version 1.7.1) (VisualSonics, Toronto, Canada).<br>The fluorescence intensity of DHE staining was measured using the ImageJ software (version 1.8.0).<br>The myocyte cross-sectional area and picrosirius red staining were quantitatively measured by ImagePro Plus software (version 7.0, Media Cybernetics Rockville, MD).<br>Proteomics data analyzed by Thermo Proteome Discoverer 1.4 software.<br>qPCR were quantitated using the 2−ΔΔCT method and normalized to the amount of endogenous Glyceraldehyde-3-phosphate dehydrogenase (GAPDH).

For manuscripts utilizing custom algorithms or software that are central to the research but not yet described in published literature, software must be made available to editors and reviewers. We strongly encourage code deposition in a community repository (e.g. GitHub). See the Nature Portfolio guidelines for submitting code & software for further information.

Data

Policy information about availability of data

All manuscripts must include a data availability statement. This statement should provide the following information, where applicable:

- Accession codes, unique identifiers, or web links for publicly available datasets
- A description of any restrictions on data availability
- For clinical datasets or third party data, please ensure that the statement adheres to our policy

The hierarchical cluster heatmap, venn diagram and KEGG pathway data used in this study are available in the GSE18801 dataset (www.ncbi.nlm.nih.gov/geo/query/acc.cgi?acc=GSE18801). The mass spectrometry proteomics data have been deposited to the ProteomeXchange Consortium via the PRIDE partner repository with the dataset identifier PXD039587. Source data are provided source data files. Additional relevant data are available at request.

Human research participants

Policy information about studies involving human research participants and Sex and Gender in Research.

Reporting on sex and gender	None
Population characteristics	None
Recruitment	None
Ethics oversight	None

Note that full information on the approval of the study protocol must also be provided in the manuscript.

Field-specific reporting

Please select the one below that is the best fit for your research. If you are not sure, read the appropriate sections before making your selection.

☒ Life sciences ☐ Behavioural & social sciences ☐ Ecological, evolutionary & environmental sciences

For a reference copy of the document with all sections, see nature.com/documents/nr-reporting-summary-flat.pdf

Life sciences study design

All studies must disclose on these points even when the disclosure is negative.

Sample size	We chose the sample sizes based on the literature, which was sufficient to analyze significance between groups. Please see: Tran et al., 2020 (PMID: PMC7156663), Foinquinos et al., 2020 (PMID: PMC6994493), Ye et al., 2020 (PMID: 32098592). The number of the independent experiments for cell and biological experiments is indicated in the Source Data.
Data exclusions	Sick or diseased animals were excluded. The exclusions were based on criteria from the literature and the recommendation of the veterinarian. The exclusions criteria were unbiasedly applied on the described experiments involving mouse model.
Replication	All experiments where reproduced, typically in triplicate, and the number of biological replicates for each experiment are noted. Replication attempts were successful.
Randomization	Animals were randomized into groups with the same genotypes, gender, and age. Mice were bred in-house with groups being populated with mice as soon as they became available. Mice were randomly assigned to groups where applicable. Experimental groups provide male mice.
Blinding	Individuals collecting data or adjudicating outcomes were blinded to the groups or cohorts being analyzed.

Reporting for specific materials, systems and methods

We require information from authors about some types of materials, experimental systems and methods used in many studies. Here, indicate whether each material, system or method listed is relevant to your study. If you are not sure if a list item applies to your research, read the appropriate section before selecting a response.

Materials & experimental systems

n/a	Involved in the study	
☐	☒	Antibodies
☐	☒	Eukaryotic cell lines
☒	☐	Palaeontology and archaeology
☐	☒	Animals and other organisms
☒	☐	Clinical data
☒	☐	Dual use research of concern

Methods

n/a	Involved in the study	
☒	☐	ChIP-seq
☒	☐	Flow cytometry
☒	☐	MRI-based neuroimaging

Antibodies

Antibodies used	p-p38 (CST, 4511, 1:1000), p38 (CST, 8690, 1:2000), p-Erk (CST, 9101, 1:2000), Erk (CST, 9102, 1:2000), p-JNK (CST, 4668, 1:1000), JNK (Abcam, ab179461, 1:2000), Bax (CST, 2772, 1:2000), Bcl-2 (Santa cruz, sc-7382, 1:1000), FGF1 (Abcam, ab207321, 1:1000), FGF2 (Santa cruz, sc-74412, 1:1000), FGF3 (Santa cruz, sc-135, 1:1000), FGF5 (Santa cruz, sc-376264, 1:1000), FGF9 (Santa cruz, sc-373716, 1:1000), FGF13 (Affinity biosciences , DF4699, 1:1000), FGF16 (Santa cruz, sc-390547, 1:1000), FGF18 (Santa cruz, sc-393471, 1:1000), FGFR3 (Santa cruz, sc-390423, 1:1000), FYN (Santa cruz, sc-365913, 1:1000), p-Src (CST, 2101, 1:1000), 3-NT (Abcam, ab61392, 1:1000), NOX4 (Origene, TA349083, 1:1000), p22phox (Santa cruz, sc-271968, 1:1000), GAPDH (Abcam, ab9485, 1:1000), HRP-goat anti-mouse (Abcam, ab6789, 1:5000), HRP-goat anti-rabbit (Abcam, ab6721, 1:5000).
Validation	p-p38, p38, p-JNK, JNK, FGF1, GAPDH, HRP-goat anti-mouse, HRP-goat anti-rabbit have been validated for immunoblotting applications and validation information can be found on the manufacturer's website.<br>p-Erk: validated by Nat Commun. 2022. PMCID: PMC9579145<br>Erk: validated by Nat Commun . 2022. PMCID: 36572689<br>Bax: validated by EMBO J. 2022. PMCID: PMC8762556<br>Bcl-2: validated by Cell. 2022. PMID: 35447071<br>FGF2: validated by Adv Sci (Weinh). 2021. PMID: 34791825<br>FGF3: validated by Chest. 2004. PMID: 15078761<br>FGF5: validated by Int J Mol Sci. 2022. PMID: 36233155<br>FGF9: validated by Cell Rep. 2018. PMID: 30590054<br>FGF13: validated by Diabetes. 2023. PMID:36256844<br>FGF16: validated by Elife. 2022. PMID: 36399125<br>FGF18: validated by Pharmacol Res. 2022. PMID: 35202822<br>FGFR3: validated by Nat Commun. 2021. PMID: 34282140<br>FYN: validated by Nat Commun. 2017. PMID: 28368000<br>p-Src: validated by Nat Commun. 2022. PMCID:PMC9345940<br>3-NT: validated by Diabetes. 2021. PMCID: PMC7897347<br>NOX4: validated by Antioxid. Redox Signal. 2016. PMCID: PMC5444494<br>p22phox: validated by Cell Death Discov. 2022. PMID: 35190552

Eukaryotic cell lines

Policy information about cell lines and Sex and Gender in Research

Cell line source(s)	HEK293T cells were obtained from Procell Life Science&Technology (CL-0005).
Authentication	HEK293T cells was authenticated by PCR assays with species-specific primer in Procell Life Science&Technology, China.
Mycoplasma contamination	Cells tested negative for mycoplasma before their use in this study.
Commonly misidentified lines<br>(See ICLAC register)	No commonly misidentified cell lines were used in this study.

Animals and other research organisms

Policy information about studies involving animals; ARRIVE guidelines recommended for reporting animal research, and Sex and Gender in Research

Laboratory animals	All animal experiments and methods performed in this study followed ethical guidelines for animal studies and were approved by the Institutional Animal Care and Use Committee of Wenzhou Medical University, China.<br>All adult (6-week-old) male C57BL/6J mice and female pregnant Sprague-Dawley rats (2 weeks pregnant) were obtained from the Model Animal Research Center of Nanjing University and were housed in an environmentally controlled room for 4-6 days to adapt to the environment before experimentation. All mice were kept in a standard laboratory condition of temperature 21±2℃, relative humidity 50±15%, 12 h light-darkness cycles, with water and food available ad libitum.<br>Both Fgf18 heterozygous (Fgf18+/-KO) and Fgf18 flox/flox (Fgf18 f/f) male mice on C57BL/6J background was a generous gift from professor Shen of Wenzhou University
Wild animals	No wild animals were used in this study.

Reporting on sex	Experimental groups provide male mice.
Field-collected samples	No field-collected samples were used in this study.
Ethics oversight	All animal experiments and methods performed in this study followed ethical guidelines for animal studies and were approved by the Institutional Animal Care and Use Committee of Wenzhou Medical University, China.

Note that full information on the approval of the study protocol must also be provided in the manuscript.
