# Supplementary material for: Fibroblast growth factor 18 alleviates stress-induced pathological cardiac hypertrophy in male mice
Source: Nat Commun. 2023 Mar 4;14:1235. doi: 10.1038/s41467-023-36895-1 (PMC9985628; doi:10.1038/s41467-023-36895-1)
Supplement: Supplementary file 3 — Source Data [file 41467_2023_36895_MOESM3_ESM.zip › 22-09253B_Source Data file/F1/F1 a-c/fig1a code.docx]

annotation_col = data.frame(Group = factor(c(rep("ISO",3),rep("Swim",3),rep("Ctrl",3))))

rownames(annotation_col) = colnames(DEG_GSE18801_exp)[c(-10,-11)]

annotation_row = data.frame(ISO.Versus.Ctrl = DEG_GSE18801$ISO_type,Swim.Versus.Ctrl = DEG_GSE18801$type)

rownames(annotation_row) = rownames(DEG_GSE18801_exp)

ann_colors = list(Group = c(ISO = "#3aa32b",Swim = "#fa5454",Ctrl = "#ac930c"),

ISO.Versus.Ctrl = c(Up = "#d250a2",Down = "#4baea5",NoSignificant = "#7887be"),

Swim.Versus.Ctrl = c(Up = "#d250a2",Down = "#4baea5",NoSignificant = "#7887be"))

p <- pheatmap(DEG_GSE18801_exp[,1:9], annotation_col = annotation_col,annotation_row = annotation_row,#

annotation_colors = ann_colors,scale = "row",clustering_method = "average",angle_col = 90)#angle_col调节角度

ggsave("./Figure1/GSE18801_FGF_heatmap.pdf",p,width=10,height=8)
