# Supplementary material for: Fibroblast growth factor 18 alleviates stress-induced pathological cardiac hypertrophy in male mice
Source: Nat Commun. 2023 Mar 4;14:1235. doi: 10.1038/s41467-023-36895-1 (PMC9985628; doi:10.1038/s41467-023-36895-1)
Supplement: Supplementary file 3 — Source Data [file 41467_2023_36895_MOESM3_ESM.zip › 22-09253B_Source Data file/F1/F1 a-c/fig1b code.docx]

DEG_GSE18801 <- read_excel("../20211104-GSE18801结果fgf-fyn-DL.xlsx",sheet = 1,skip=1)

DEG_ISO <- DEG_GSE18801 %>% dplyr::filter(FDR...5 < 0.08 & logFC...2 < -0.322) %>% dplyr::select(1,2,3,4,5,10,11,12,13,14,15)

DEG_swim <- DEG_GSE18801 %>% dplyr::filter(FDR...9 < 0.08 & logFC...6 < -0.322) %>% dplyr::select(1,6,7,8,9,10,11,12,16,17,18)

listinput <- list(one = DEG_ISO$symbol,

two = DEG_swim$symbol)

library(VennDiagram)

venn_list <- list(ISO_Cntr = listinput$one,SWIM_Cntr = listinput$two)

p <- venn.diagram(venn_list, resolution =600,filename = NULL

col="gray",fill=c(colors()[148], colors()[589]), alpha=c(0.6, 0.6), cat.cex = 1.5, cat.fontfamily = 'serif',

cex = 1.5,fontfamily = 'serif',cat.dist = -0.04)# filename ="./Figure2/veen_figure.tiff" ,height = 1500,width = 1500,

grid.draw(p)

dev.off()
