# Supplementary material for: Fibroblast growth factor 18 alleviates stress-induced pathological cardiac hypertrophy in male mice
Source: Nat Commun. 2023 Mar 4;14:1235. doi: 10.1038/s41467-023-36895-1 (PMC9985628; doi:10.1038/s41467-023-36895-1)
Supplement: Supplementary file 3 — Source Data [file 41467_2023_36895_MOESM3_ESM.zip › 22-09253B_Source Data file/F1/F1 a-c/fig1c code.docx]

#气泡图----

rm(list = ls())

library(readr)

library(readxl)

library(tidyverse)

library(mschart)

windowsFonts(RMN=windowsFont("Times New Roman"))

DEG_GSE18801 <- read_excel("./3_气泡图_Figure3_dotplot.xlsx",sheet = 1)

ISO_BP <- DEG_GSE18801[-10,]

colnames(ISO_BP)[1:3] <- c("ONTOLOGY","ID","Description")

colnames()

#visualization----

#计算rich factor

enrichment_fold=apply(ISO_BP,1,function(x){

GeneRatio=eval(parse(text=x["GeneRatio"]))

BgRatio=eval(parse(text=x["BgRatio"]))

enrichment_fold=round(GeneRatio/BgRatio,2)

enrichment_fold

})

ISO_BP$Rich_Factor <- enrichment_fold

ISO_BP$Description <- factor(ISO_BP$Description,levels = c("tissue remodeling","cardiac muscle hypertrophy","cardiac muscle tissue growth","superoxide anion generation","response to hydrogen peroxide","response to oxidative stress","response to reactive oxygen species","regulation of apoptotic signaling pathway", "regulation of cell shape","heart process","heart contraction","cellular response to decreased oxygen levels","response to fibroblast growth factor","cellular response to fibroblast growth factor stimulus"))

p <- ggplot(ISO_BP,aes(Rich_Factor,Description))+

geom_point(aes(size=Count,color=-1*log10(pvalue)))+

scale_color_gradient(low="#4fc3f7",high = "#f06292")

p2 <- p + theme(#legend.title = element_blank(),

legend.key=element_blank(),

legend.text = element_text(color="black",size=10),

legend.spacing.x=unit(0.1,'cm'),

legend.key.width=unit(0.5,'cm'),

legend.key.height=unit(0.5,'cm'),

legend.background=element_blank(),

legend.box.background=element_rect(colour = "black"),

legend.box.margin = margin(1,1,1,1))

p2

p3 <- p2 + theme(legend.position = c(1, 1), legend.justification = c(1, 1))

ggsave("./Figure3/dotplot.pdf",p3,width=5,height=5)
