# Supplementary material for: Fibroblast growth factor 18 alleviates stress-induced pathological cardiac hypertrophy in male mice
Source: Nat Commun. 2023 Mar 4;14:1235. doi: 10.1038/s41467-023-36895-1 (PMC9985628; doi:10.1038/s41467-023-36895-1)
Supplement: Supplementary file 3 — Source Data [file 41467_2023_36895_MOESM3_ESM.zip › 22-09253B_Source Data file/F5/F5 a-b/Fig5 a code.docx]

#热图----Figure 5

rm(list = ls())

### 1. package------

library(pheatmap)

library(tidyverse)

library(data.table)

library(readr)

library(readxl)

library(Hmisc)

### 2.data import----

GSE18801 <- read_excel("F:/WEN_cooperate/20211105/20211104-GSE18801结果fgf-fyn-DL.xlsx",skip = 1,sheet = 1)

gene <- c("Src","Blk","Fgr","Fyn","Hck","Lyn","Lck","Yes","Yes1")

GSE18801 <- GSE18801 %>% dplyr::filter(symbol %in% gene)

GSE18801 <- GSE18801 %>% dplyr::mutate(type = ifelse(adj.P.Val...4 < 0.08 & logFC...2 > 0.322,"Up",ifelse(adj.P.Val...4 < 0.08 & logFC...2 < -0.322,"Down","NoSignificant")))

#鼠的这些基因的case和cntr的均值---

GSE18801_data <- GSE18801 %>% dplyr::select(1,10,11,12,13,14,15,19)

GSE18801_data <- GSE18801_data %>% column_to_rownames("symbol")

GSE18801_case_mean <- apply(GSE18801_data,1,function(x){mean(as.numeric(x[4:6]))})

GSE18801_cntr_mean <- apply(GSE18801_data,1,function(x){mean(as.numeric(x[1:3]))})

#合并数据结果

GSE18801_data <- cbind(GSE18801_cntr_mean,GSE18801_case_mean,GSE18801$type) %>% as.data.frame() %>% rownames_to_column("symbol")

data <- GSE18801_data %>% column_to_rownames("symbol")

data1 <- cbind(GSE18801_cntr_mean,GSE18801_case_mean)%>% as.data.frame()

data1 <- scale(data1,center = F,scale = T) %>% as.data.frame()

#画图----

# data1 <- data1 %>% dplyr::select(1,3,2,4)

colnames(data1) <- c("Ctrl","ISO")

annotation_col = data.frame(group = factor(c("Ctrl","ISO")))

rownames(annotation_col) = colnames(data1)

annotation_row = data.frame(Regulation = data$V3)

rownames(annotation_row) <- rownames(data1)

ann_colors = list(group = c(Ctrl = "#ac930c",ISO = "#3aa32b"),

Regulation = c(NoSignificant = "#7887be",Down = "#4baea5"))

p <- pheatmap(data1, annotation_col = annotation_col,annotation_row = annotation_row,#

annotation_colors = ann_colors,angle_col = 90,cluster_rows = F)#,scale = "row",clustering_method = "average"

ggsave("./Figure5/heatmap.pdf",p,width=6,height=6)

#GSEA----Figure 8

rm(list = ls())

library(clusterProfiler)

library(org.Mm.eg.db)

library(GSEABase)

library(enrichplot)

load("F:/WEN_cooperate/20211016/mmuGPL1621/1.rawdata/GPL1261/GSE18801/04_Diff/fdr008_logFC0322.Rdata")

colnames(iso_diff)[1] <- "SYMBOL"

entrzID <- AnnotationDbi::select(org.Mm.eg.db, keys=iso_diff$SYMBOL,keytype="SYMBOL", columns = "ENTREZID")%>%inner_join(iso_diff,by = "SYMBOL")

geneList_iso=iso_diff$logFC

names(geneList_iso)=entrzID$ENTREZID

geneList_iso=sort(geneList_iso,decreasing = T)

gmts <- read.gmt("./Figure8/Mm.c5.all.v7.1.entrez.gmt")

egmt <- GSEA(geneList_iso, TERM2GENE=gmts, verbose=FALSE, pvalueCutoff = 1)

head(egmt)

gseaplot2(egmt,132,color="blue",pvalue_table = T) # 按第一个做二维码图，并显示p值

cat(paste(dim(egmt@result)),'\n')

GSEA_GO <- egmt@result

write.table(GSEA_GO,"./Figure8/GSEA_GO_iso.txt",sep = "\t",col.names = T,row.names = F,quote = F)

p1 <- gseaplot2(egmt,132,color="blue",pvalue_table = T) # Fgf18

p2 <- gseaplot2(egmt,1844,color="blue",pvalue_table = T) # Fyn

p3 <- gseaplot2(egmt,839,color="red",pvalue_table = T) # Nox4

p4 <- gseaplot2(egmt,500,color="red",pvalue_table = T) # ROS

ggsave("./Figure8/Fgf18_GSEA.pdf",p1,width = 10,height = 8)

ggsave("./Figure8/Fyn_GSEA.pdf",p2,width = 10,height = 8)

ggsave("./Figure8/Nox4_GSEA.pdf",p3,width = 10,height = 8)

ggsave("./Figure8/ROS_GSEA.pdf",p4,width = 10,height = 8)
