# Supplementary material for: Fibroblast growth factor 18 alleviates stress-induced pathological cardiac hypertrophy in male mice
Source: Nat Commun. 2023 Mar 4;14:1235. doi: 10.1038/s41467-023-36895-1 (PMC9985628; doi:10.1038/s41467-023-36895-1)
Supplement: Supplementary file 3 — Source Data [file 41467_2023_36895_MOESM3_ESM.zip › 22-09253B_Source Data file/F5/F5 a-b/Fig5 b code.docx]

#弦图----Figure 4

# install.packages("ggalluvial")

# install.packages("networkD3")

# install.packages("riverplot")

rm(list = ls())

library(ggplot2)

library(dplyr)

library(ggalluvial)

library(networkD3)

library(riverplot)

windowsFonts(RMN=windowsFont("Times New Roman"))

#画弦图

# install.packages("GOplot")

library(GOplot)

GO <- read_excel("./Figure4/chord.xlsx",sheet = 1) %>% as.data.frame()

diff_gene <- read_excel("./Figure4/chord.xlsx",sheet = 2) %>% as.data.frame()

colnames(diff_gene)[1] <- "ID"

colnames(GO)[5] <- "adj_pval"

circ <- circle_dat(GO,diff_gene)

df3 <- read_excel("./Figure4/chord.xlsx",sheet = 3) %>% as.data.frame()

df4 <- read_excel("./Figure4/chord.xlsx",sheet = 4) %>% as.data.frame()

df3$ID <- toupper(df3$ID)

chord <- chord_dat(data = circ,genes = df3,process = df4$x)

library(Hmisc)

rownames(chord) <- capitalize(tolower(rownames(chord)))

p1 <- GOChord(chord, space = 0.02, gene.order = 'logFC', gene.space = 0.25, gene.size = 5)

ggsave("./Figure4/chord.pdf",p1,width = 15,height = 15)
