# Supplementary figures and images for: Fibroblast growth factor 18 alleviates stress-induced pathological cardiac hypertrophy in male mice
Source: Nat Commun. 2023 Mar 4;14:1235. doi: 10.1038/s41467-023-36895-1 (PMC9985628; doi:10.1038/s41467-023-36895-1)

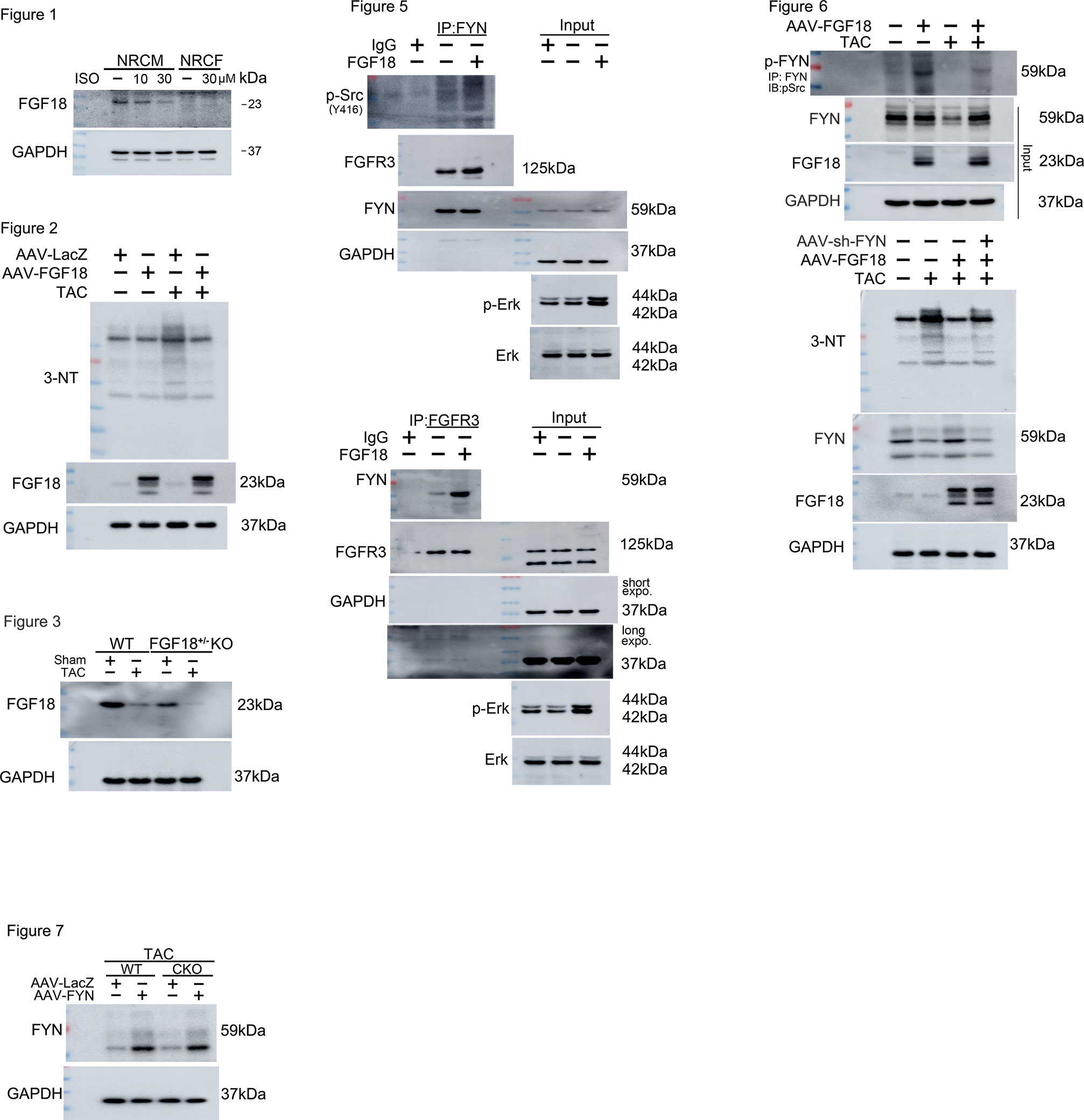

Supplement: Supplementary file 3 — Source Data [file 41467_2023_36895_MOESM3_ESM.zip › 22-09253B_Source Data file/blots and gels/blots/1 Raw data Figures.jpg]

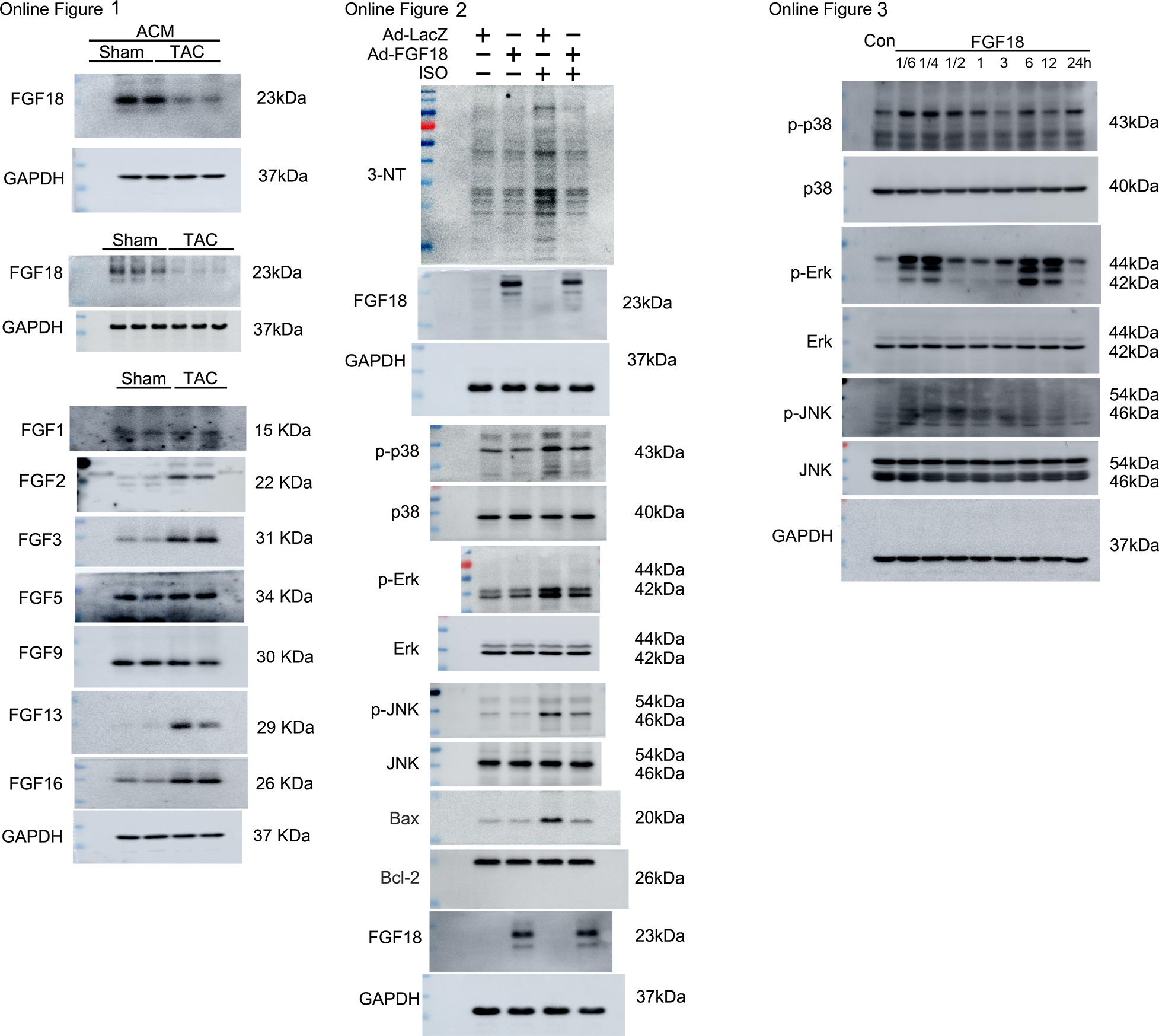

Supplement: Supplementary file 3 — Source Data [file 41467_2023_36895_MOESM3_ESM.zip › 22-09253B_Source Data file/blots and gels/blots/2 Raw data online Figures 1-3.jpg]

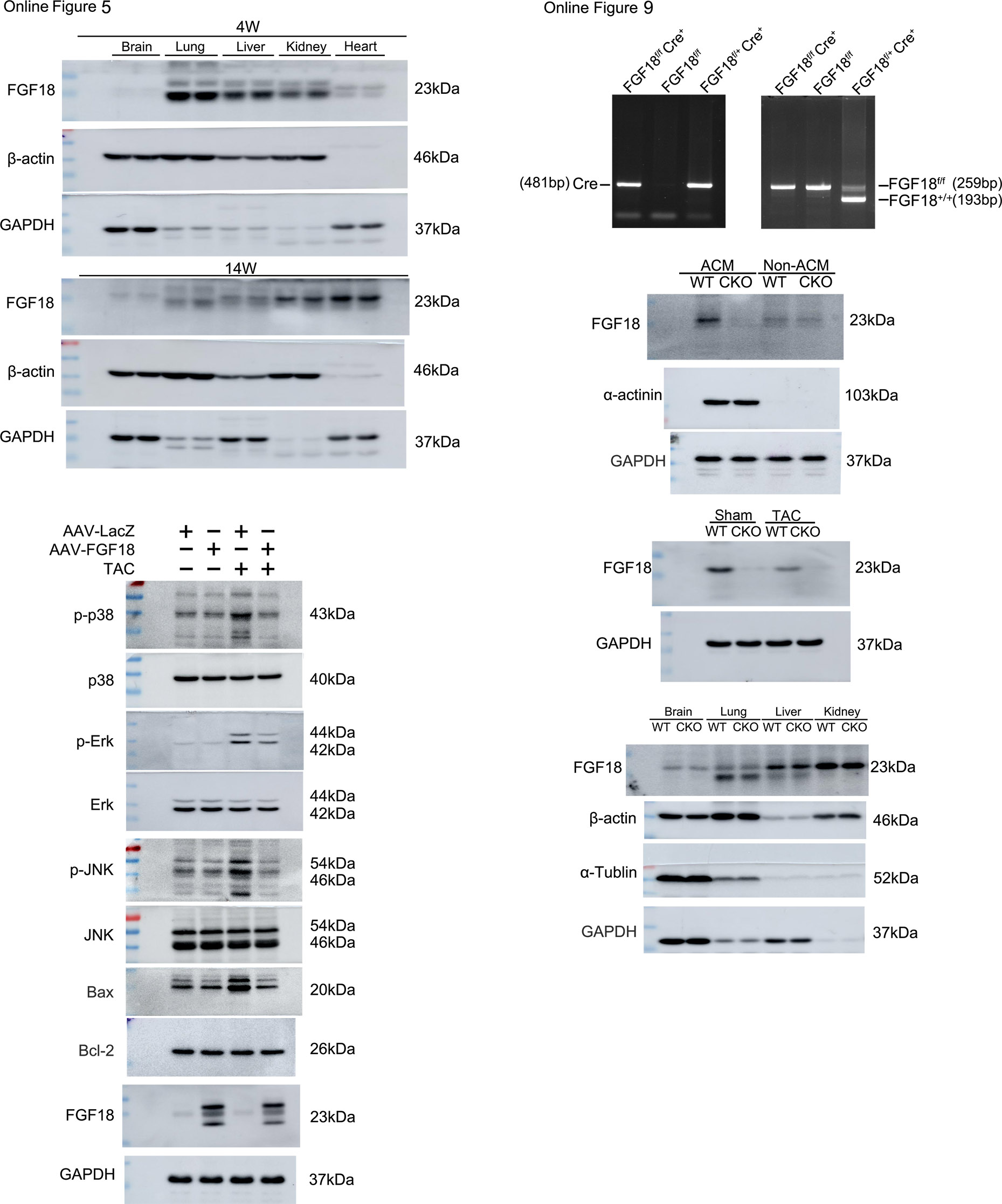

Supplement: Supplementary file 3 — Source Data [file 41467_2023_36895_MOESM3_ESM.zip › 22-09253B_Source Data file/blots and gels/blots/3 Raw data online Figures 5 and 9.jpg]

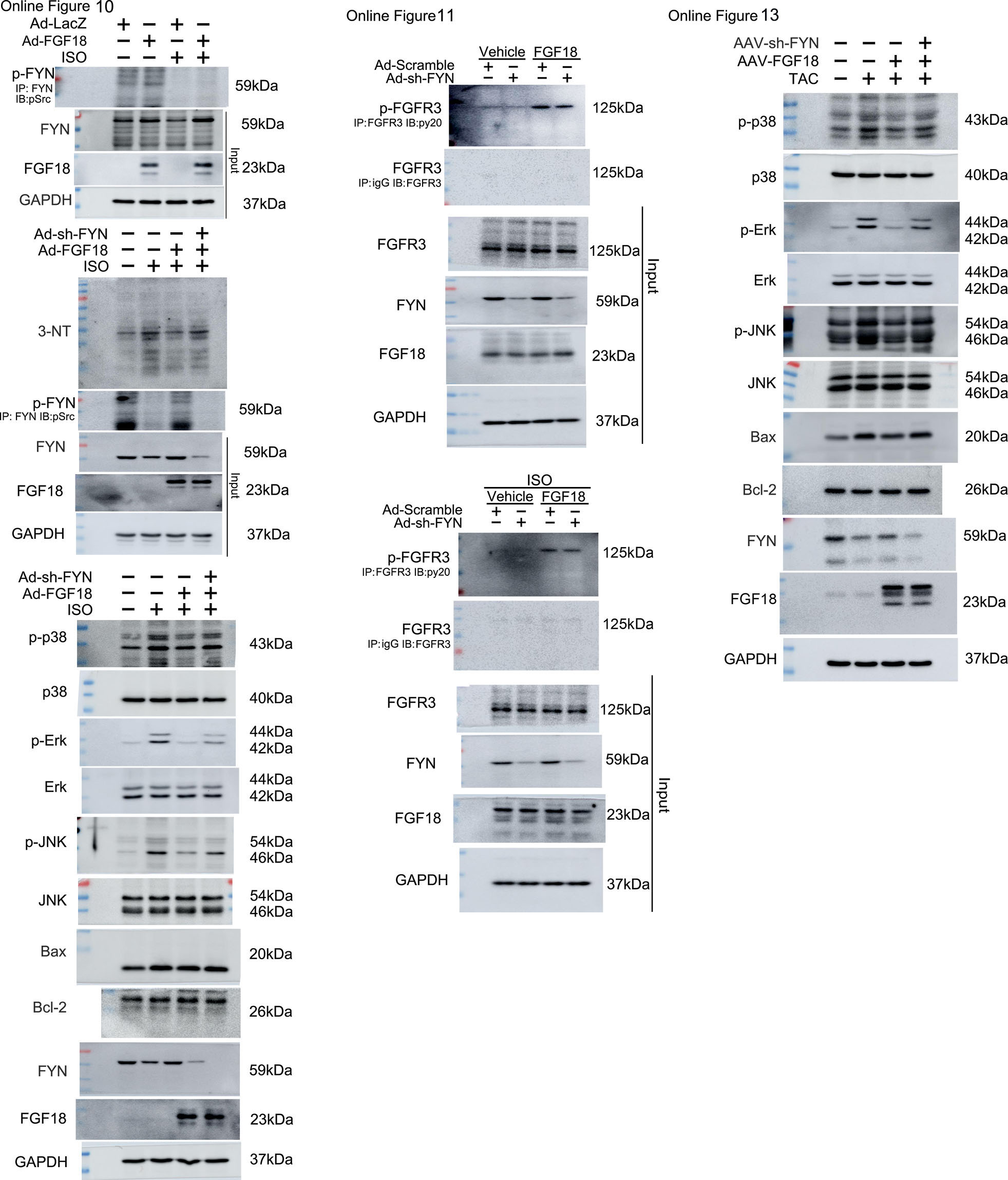

Supplement: Supplementary file 3 — Source Data [file 41467_2023_36895_MOESM3_ESM.zip › 22-09253B_Source Data file/blots and gels/blots/4 Raw data online Figures 10-13.jpg]

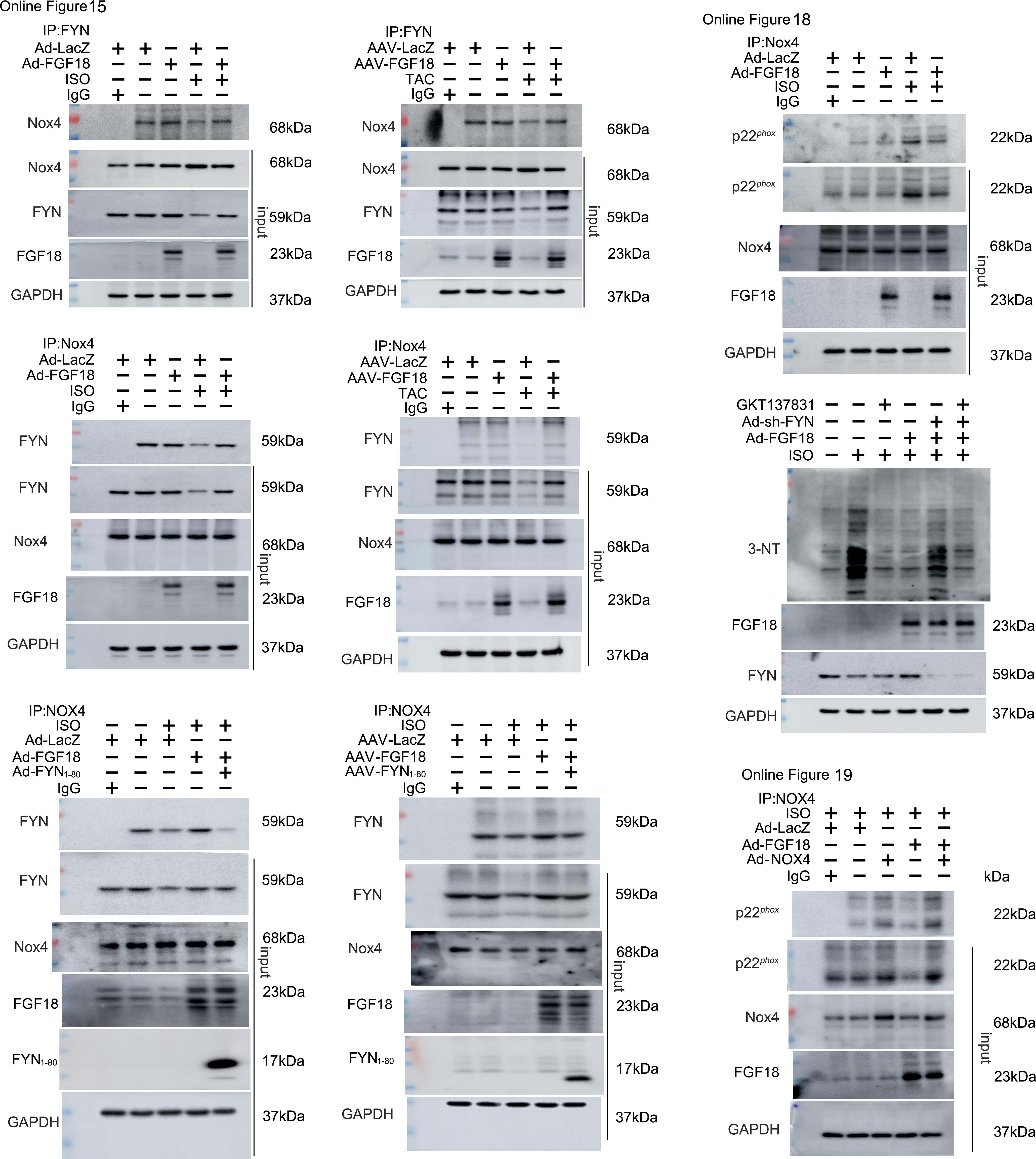

Supplement: Supplementary file 3 — Source Data [file 41467_2023_36895_MOESM3_ESM.zip › 22-09253B_Source Data file/blots and gels/blots/5 Raw data online Figures 15-19.jpg]

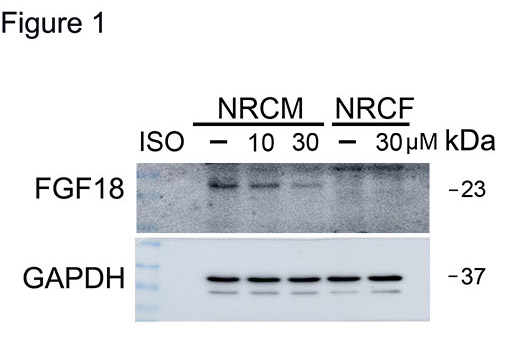

Supplement: Supplementary file 3 — Source Data [file 41467_2023_36895_MOESM3_ESM.zip › 22-09253B_Source Data file/blots and gels/blots/F1.jpg]

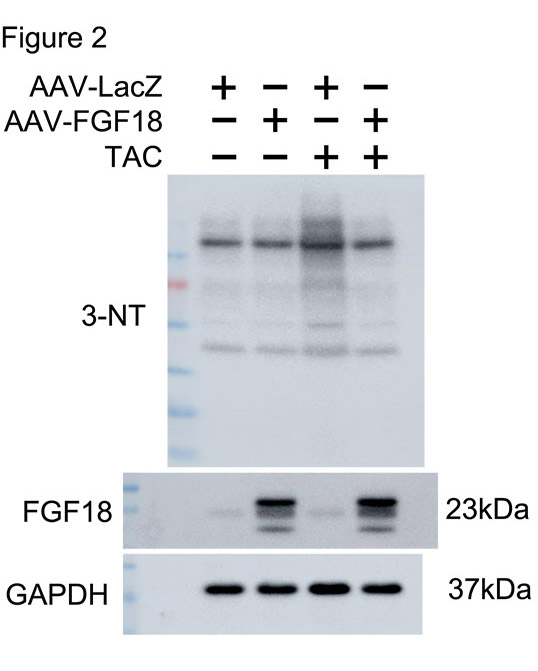

Supplement: Supplementary file 3 — Source Data [file 41467_2023_36895_MOESM3_ESM.zip › 22-09253B_Source Data file/blots and gels/blots/F2.jpg]

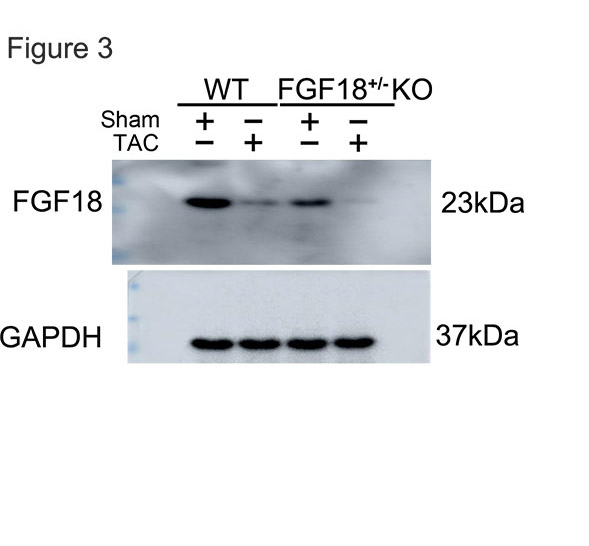

Supplement: Supplementary file 3 — Source Data [file 41467_2023_36895_MOESM3_ESM.zip › 22-09253B_Source Data file/blots and gels/blots/F3.jpg]

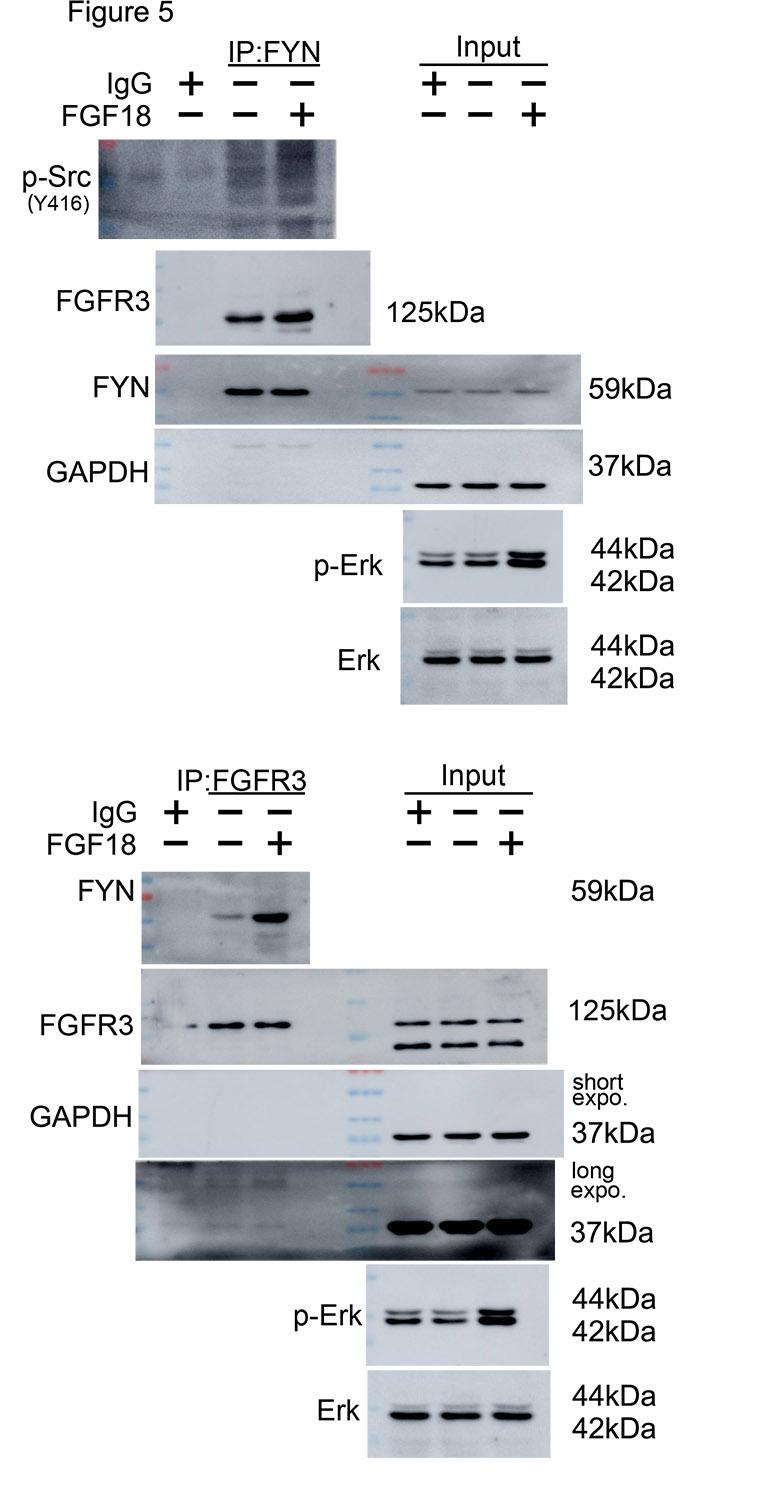

Supplement: Supplementary file 3 — Source Data [file 41467_2023_36895_MOESM3_ESM.zip › 22-09253B_Source Data file/blots and gels/blots/F5.jpg]

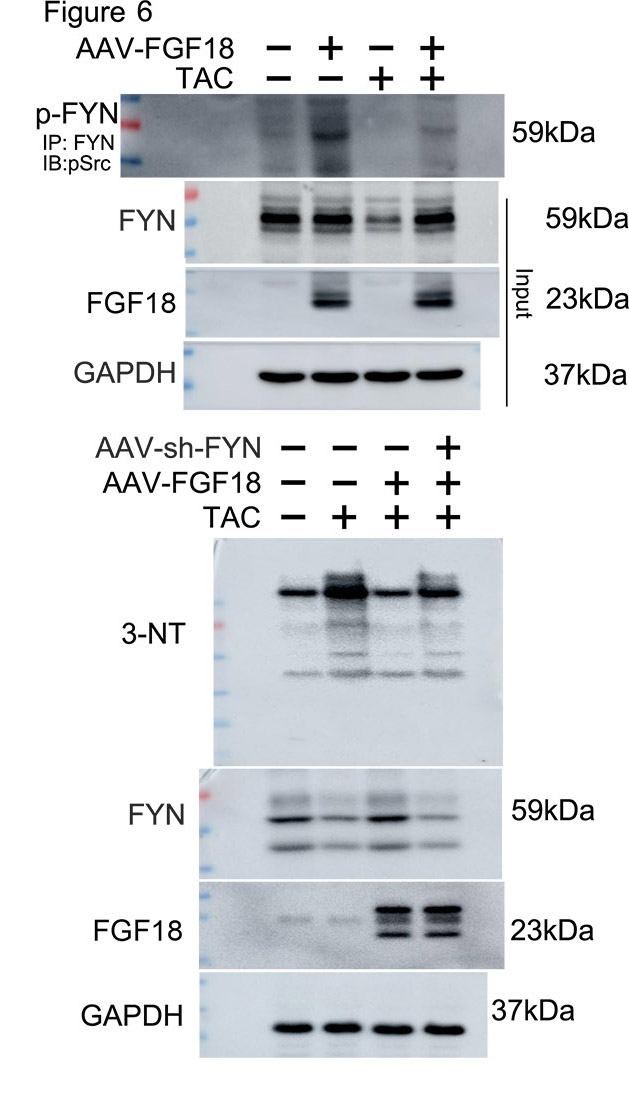

Supplement: Supplementary file 3 — Source Data [file 41467_2023_36895_MOESM3_ESM.zip › 22-09253B_Source Data file/blots and gels/blots/F6.jpg]

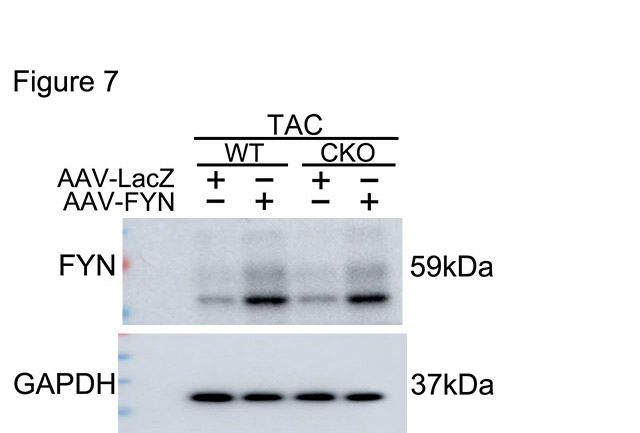

Supplement: Supplementary file 3 — Source Data [file 41467_2023_36895_MOESM3_ESM.zip › 22-09253B_Source Data file/blots and gels/blots/F7.jpg]

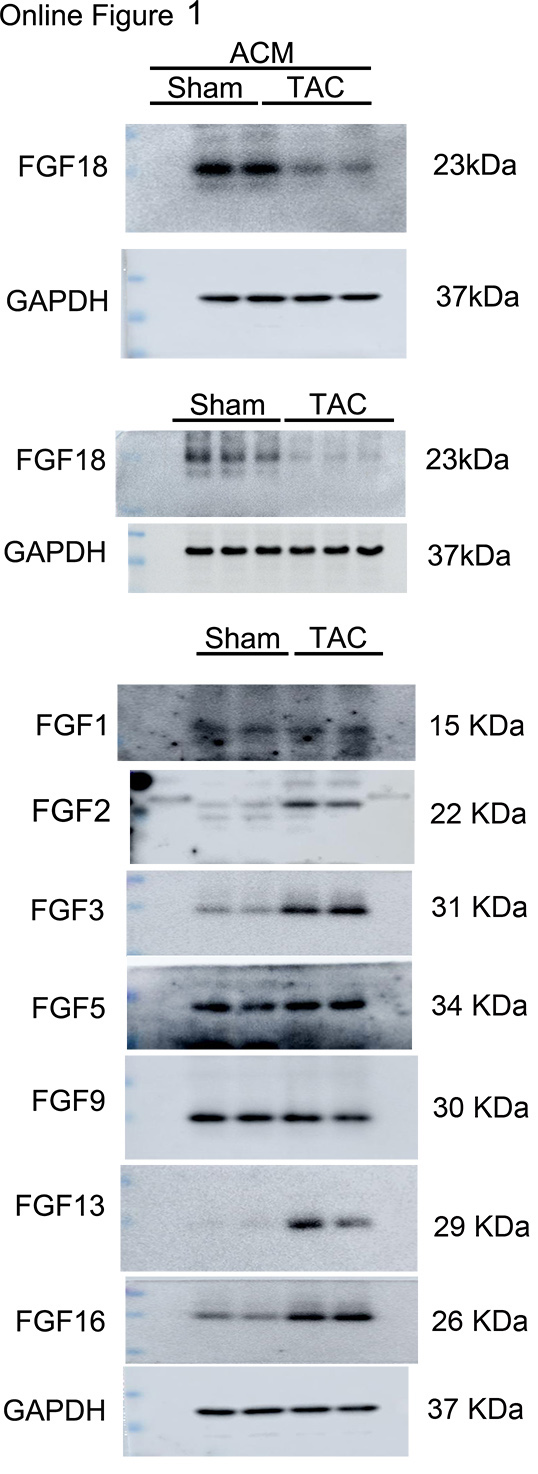

Supplement: Supplementary file 3 — Source Data [file 41467_2023_36895_MOESM3_ESM.zip › 22-09253B_Source Data file/blots and gels/blots/S1.jpg]

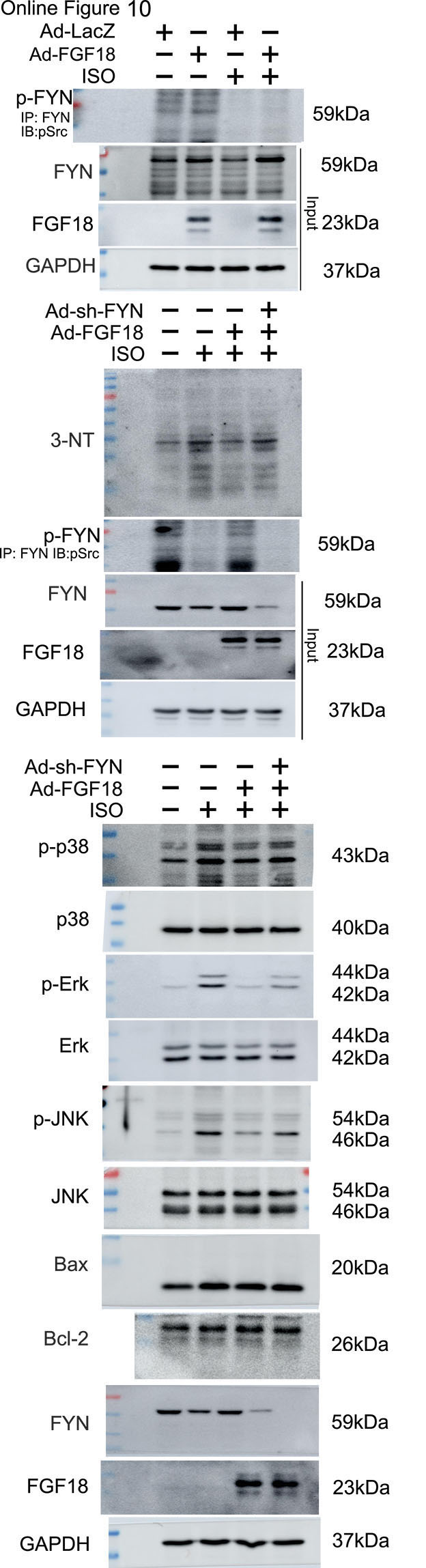

Supplement: Supplementary file 3 — Source Data [file 41467_2023_36895_MOESM3_ESM.zip › 22-09253B_Source Data file/blots and gels/blots/S10.jpg]

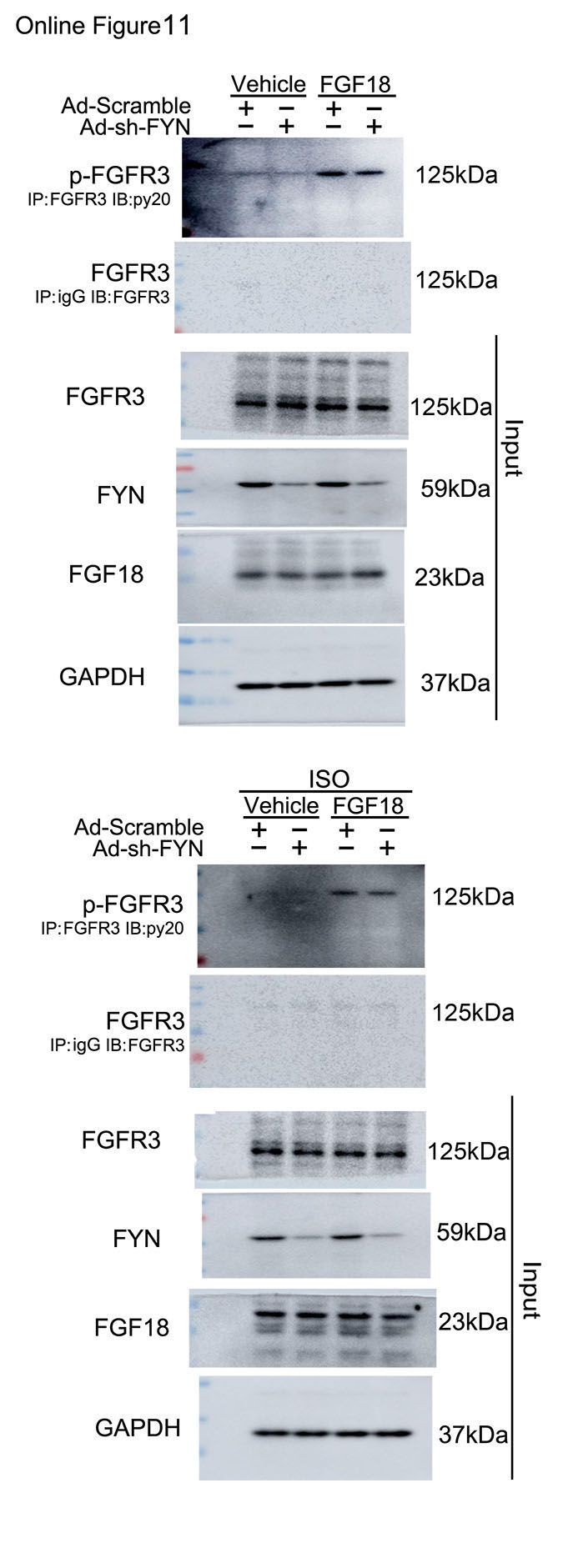

Supplement: Supplementary file 3 — Source Data [file 41467_2023_36895_MOESM3_ESM.zip › 22-09253B_Source Data file/blots and gels/blots/S11.jpg]

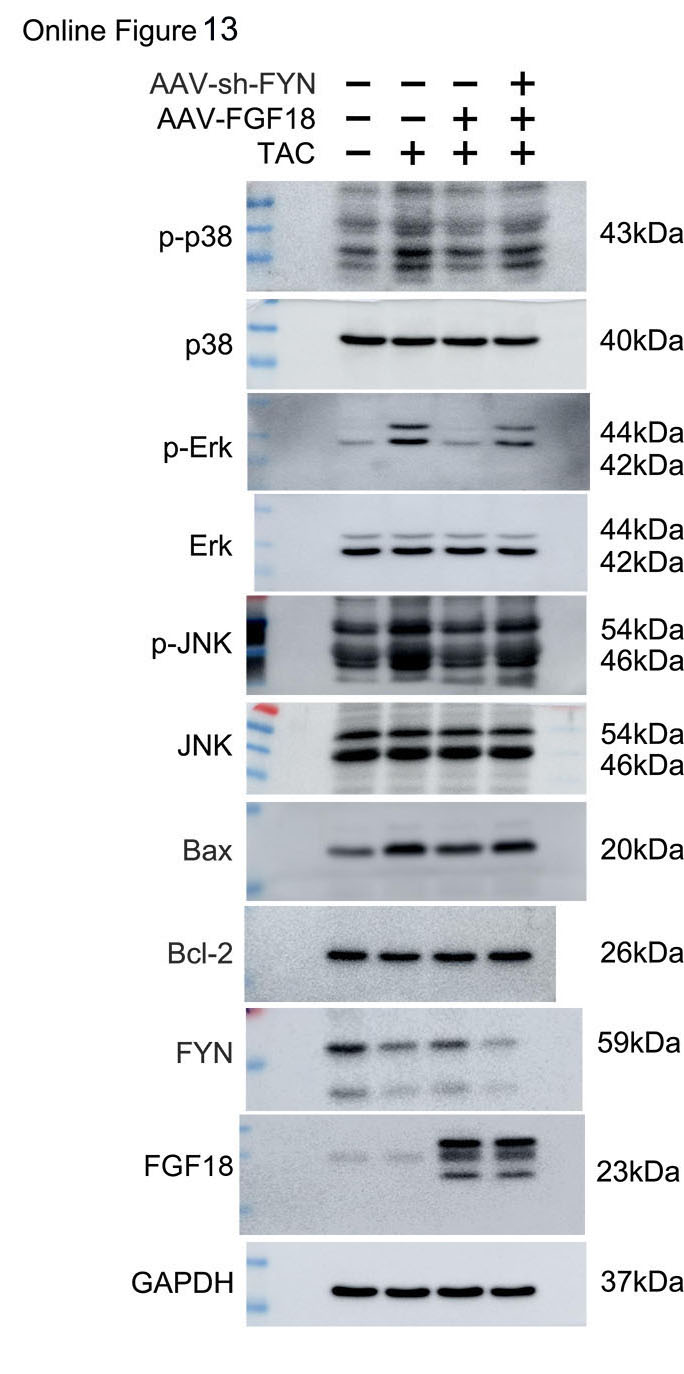

Supplement: Supplementary file 3 — Source Data [file 41467_2023_36895_MOESM3_ESM.zip › 22-09253B_Source Data file/blots and gels/blots/S13.jpg]

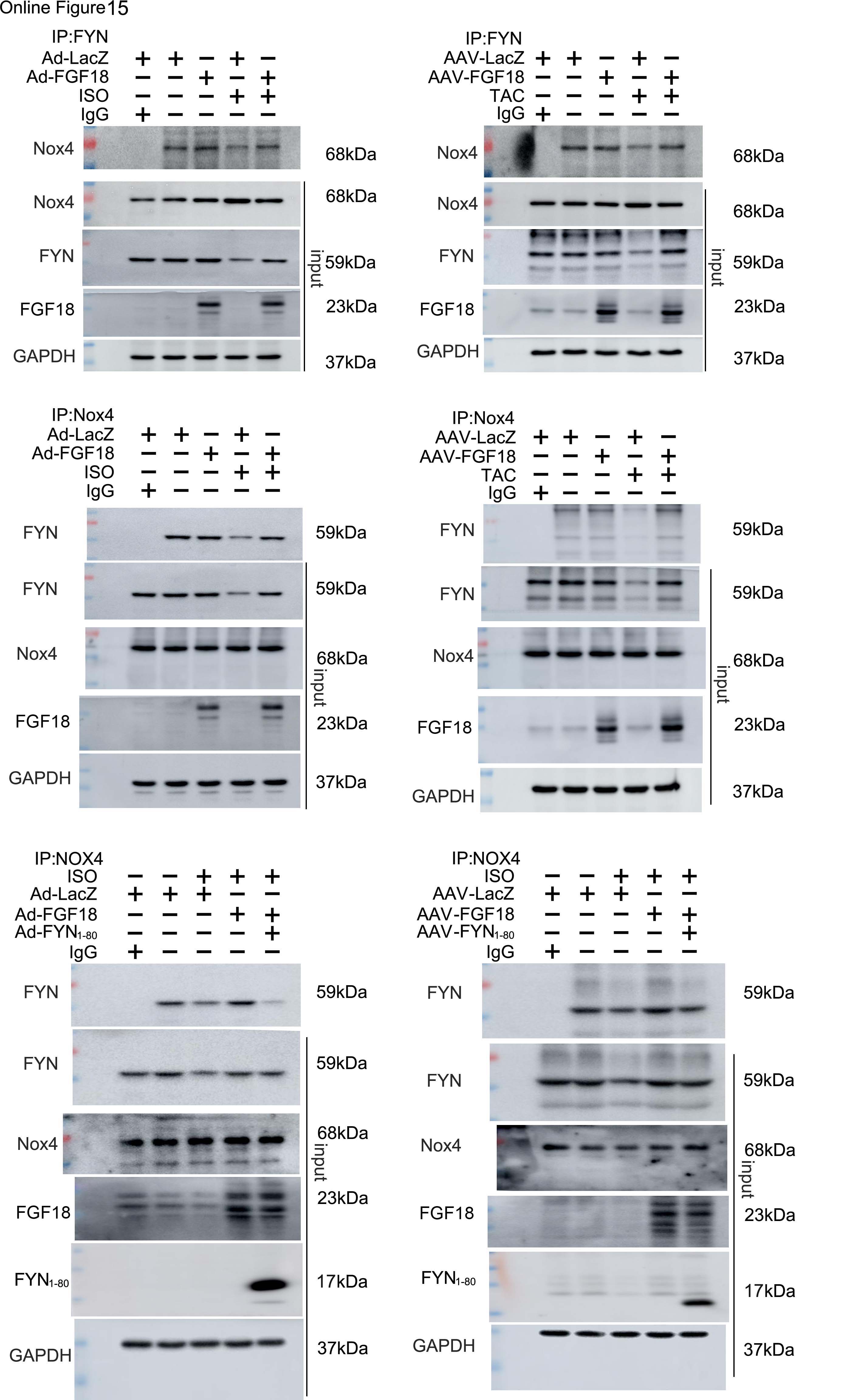

Supplement: Supplementary file 3 — Source Data [file 41467_2023_36895_MOESM3_ESM.zip › 22-09253B_Source Data file/blots and gels/blots/S15.jpg]

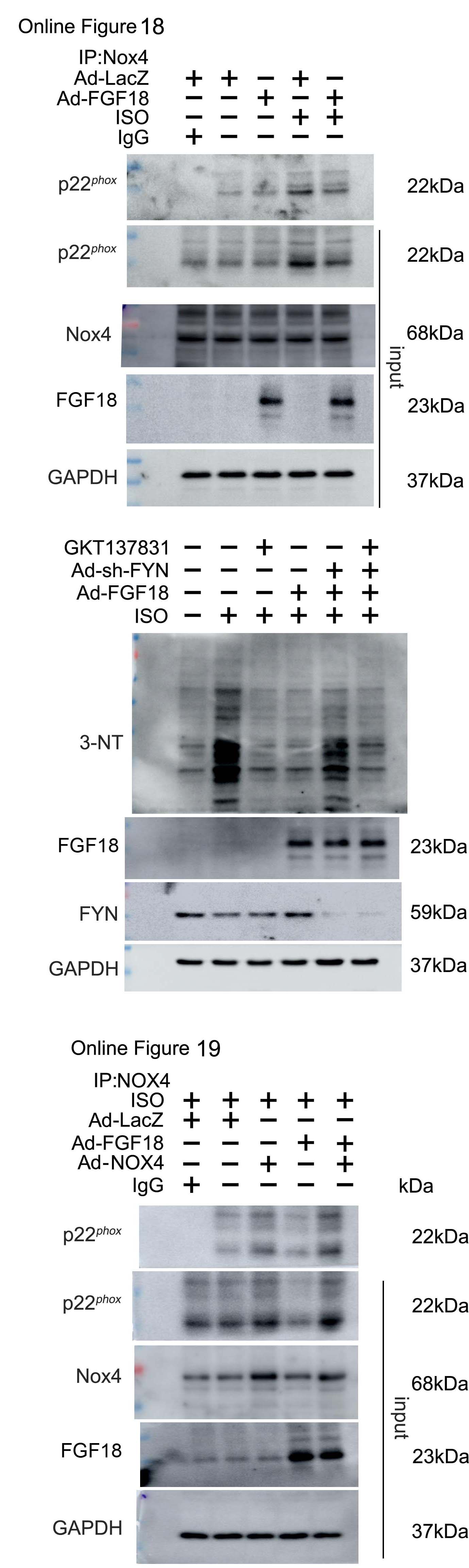

Supplement: Supplementary file 3 — Source Data [file 41467_2023_36895_MOESM3_ESM.zip › 22-09253B_Source Data file/blots and gels/blots/S18-19.jpg]

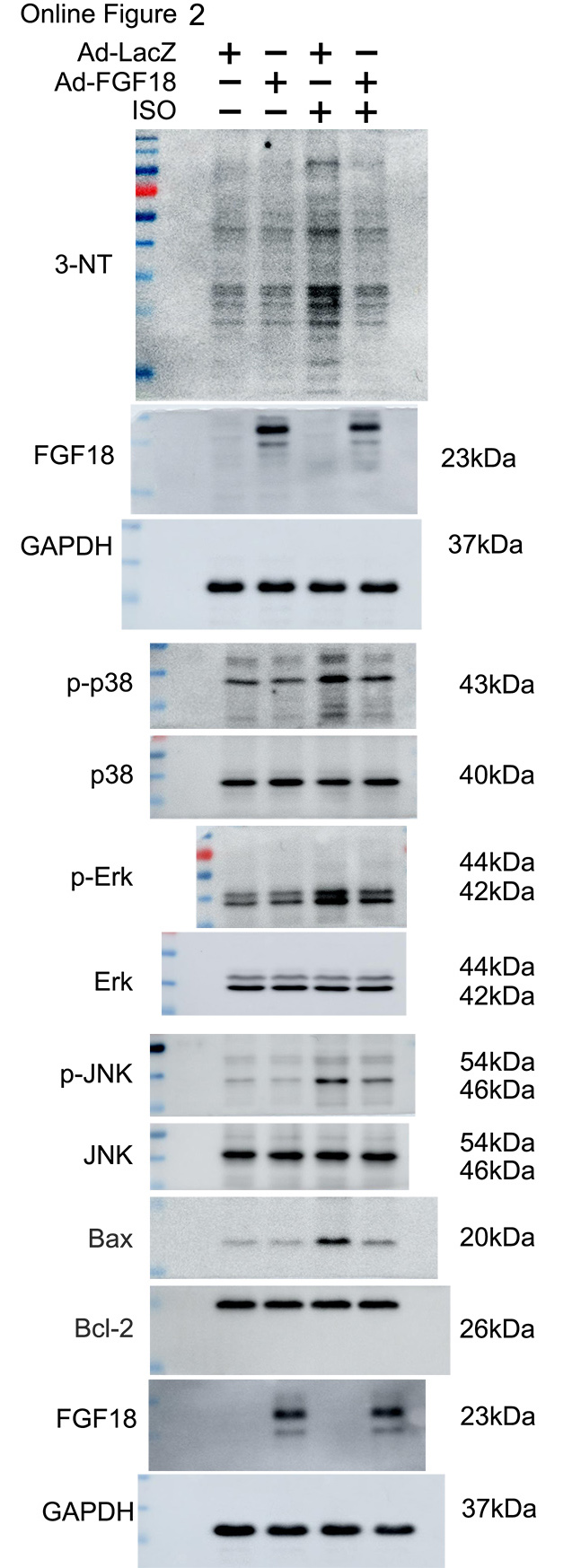

Supplement: Supplementary file 3 — Source Data [file 41467_2023_36895_MOESM3_ESM.zip › 22-09253B_Source Data file/blots and gels/blots/S2.jpg]

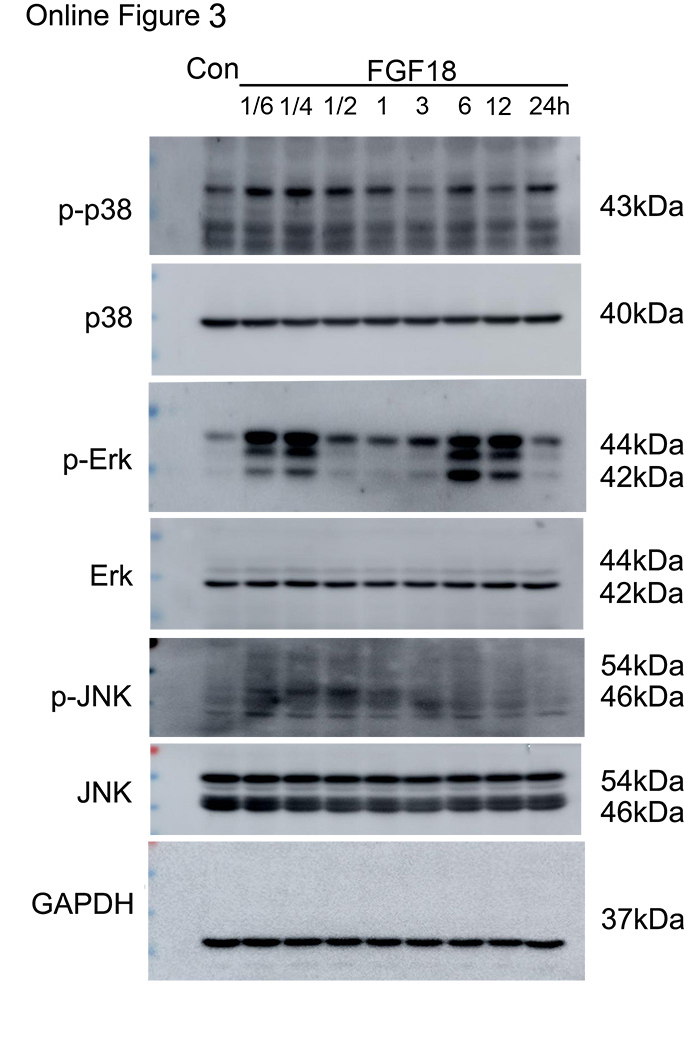

Supplement: Supplementary file 3 — Source Data [file 41467_2023_36895_MOESM3_ESM.zip › 22-09253B_Source Data file/blots and gels/blots/S3.jpg]

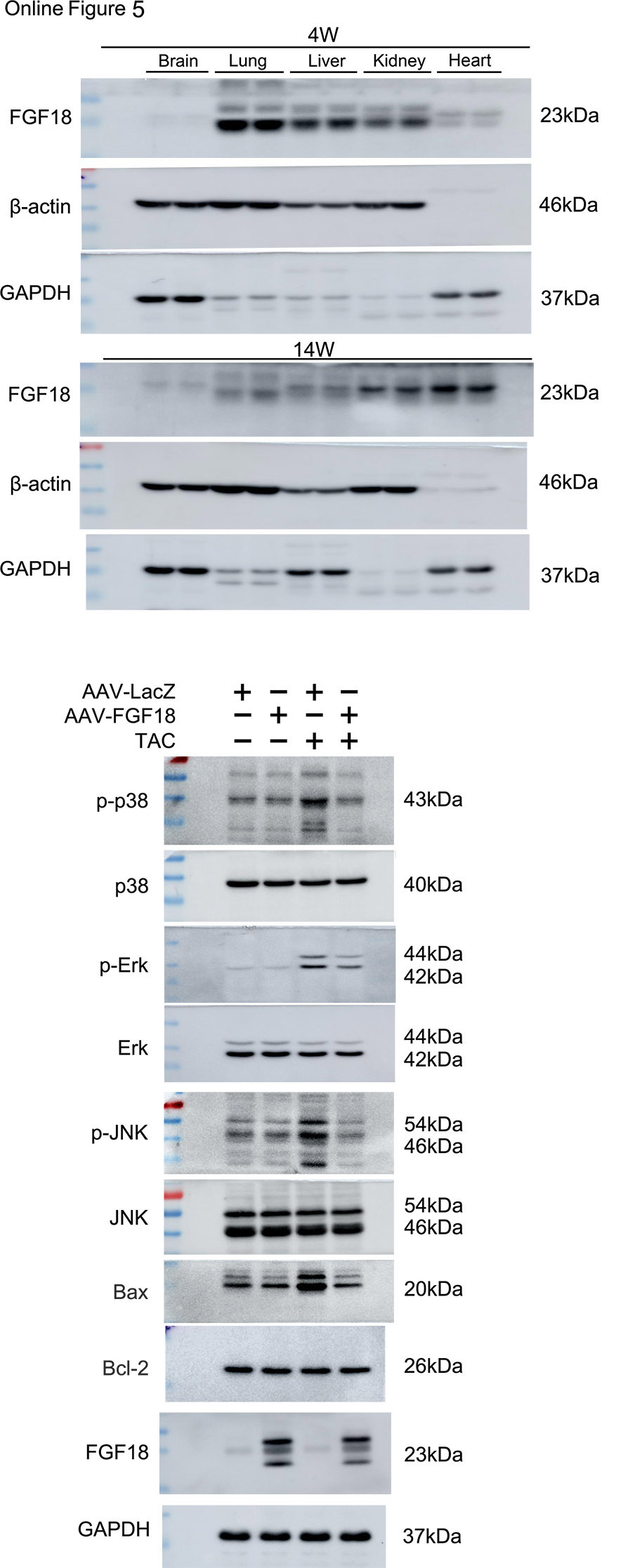

Supplement: Supplementary file 3 — Source Data [file 41467_2023_36895_MOESM3_ESM.zip › 22-09253B_Source Data file/blots and gels/blots/S5.jpg]

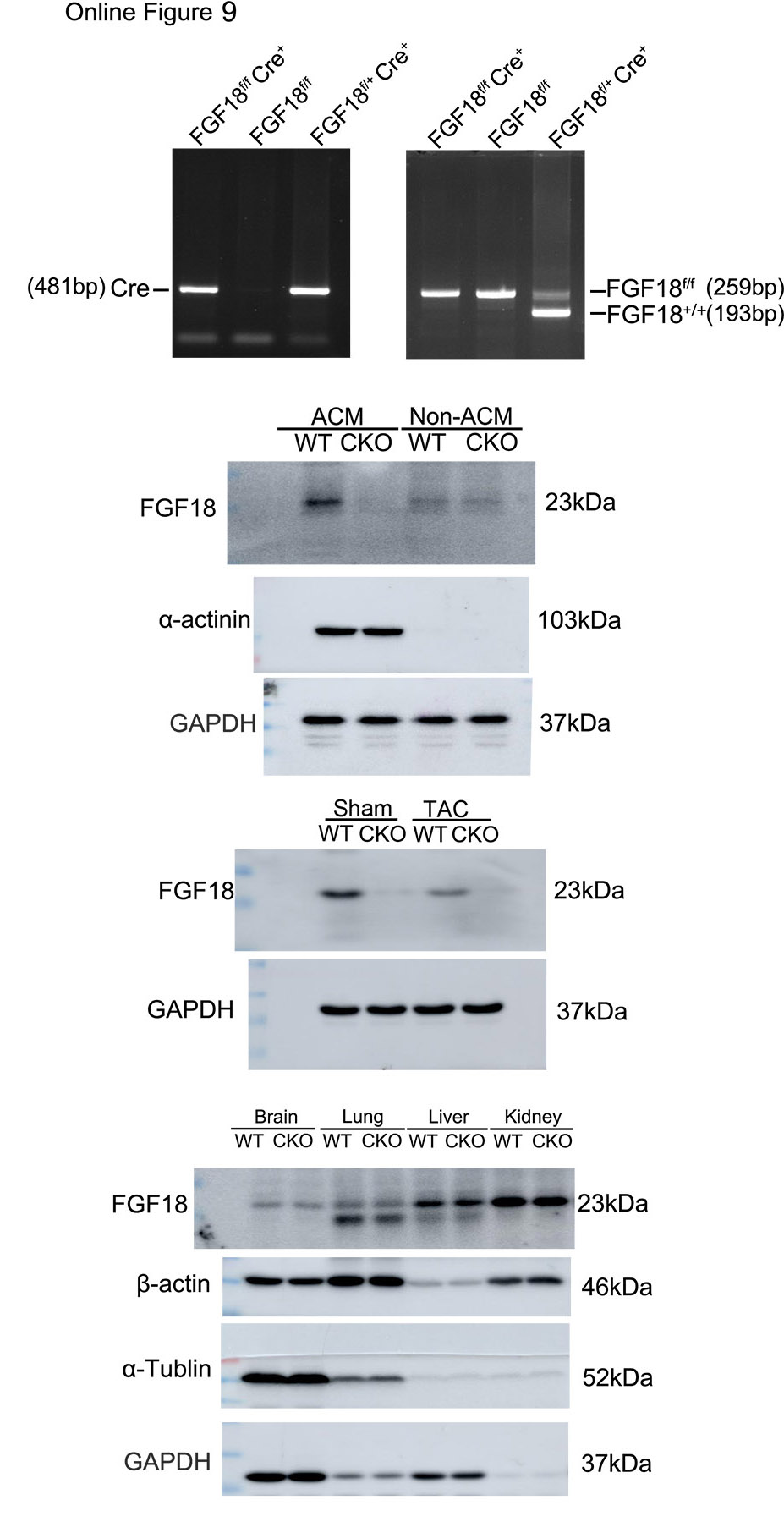

Supplement: Supplementary file 3 — Source Data [file 41467_2023_36895_MOESM3_ESM.zip › 22-09253B_Source Data file/blots and gels/blots/S9.jpg]

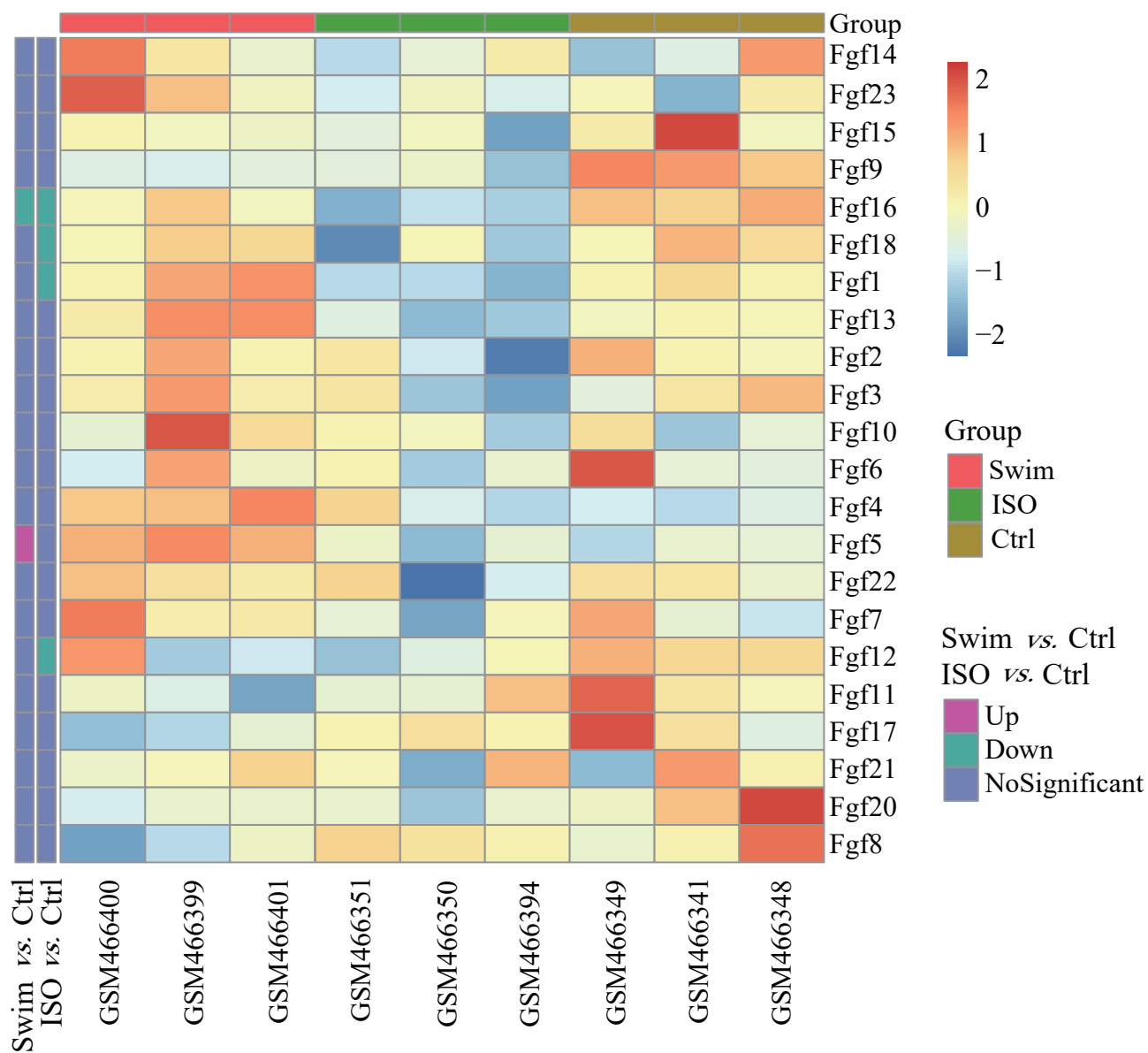

Supplement: Supplementary file 3 — Source Data [file 41467_2023_36895_MOESM3_ESM.zip › 22-09253B_Source Data file/F1/F1 a-c/fig1a.pdf]

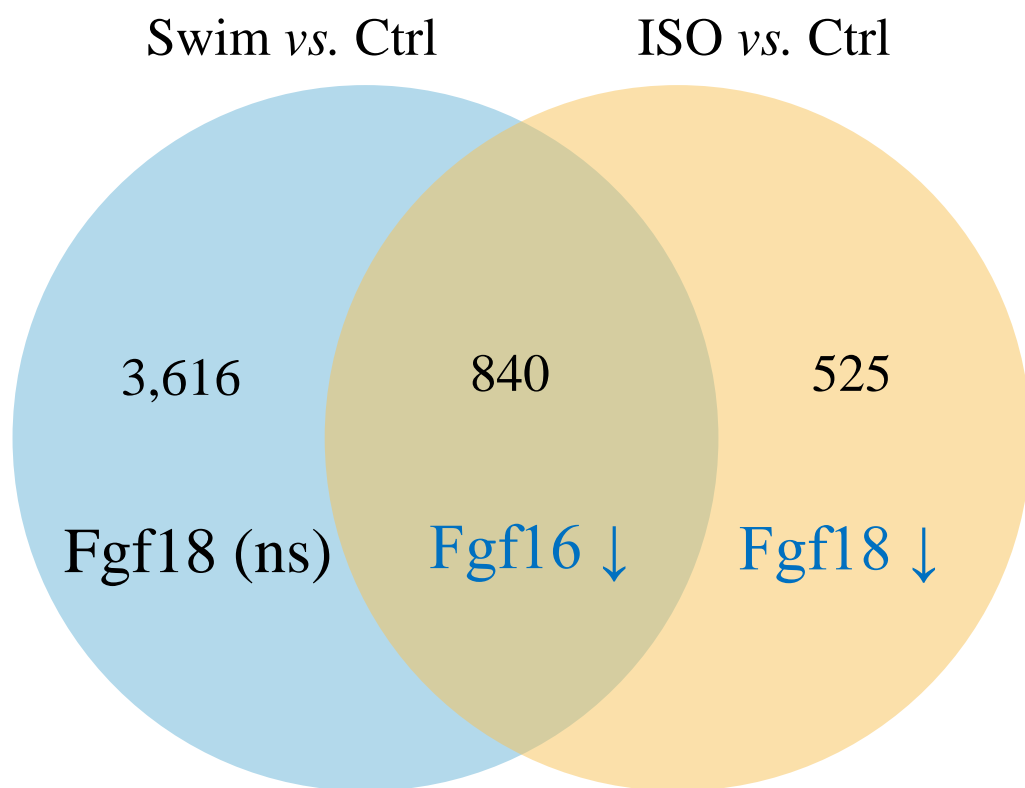

Supplement: Supplementary file 3 — Source Data [file 41467_2023_36895_MOESM3_ESM.zip › 22-09253B_Source Data file/F1/F1 a-c/fig1b.pdf]

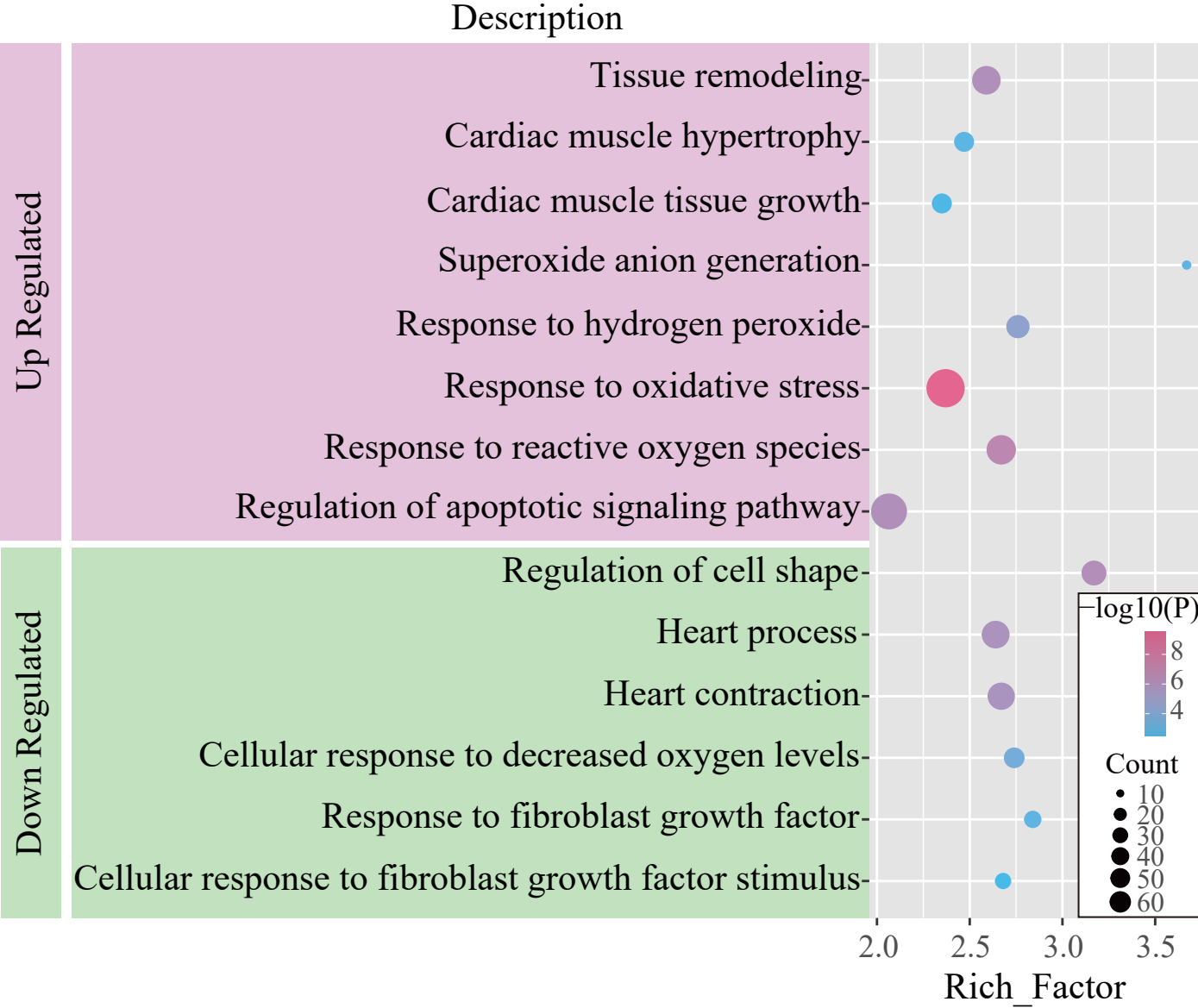

Supplement: Supplementary file 3 — Source Data [file 41467_2023_36895_MOESM3_ESM.zip › 22-09253B_Source Data file/F1/F1 a-c/fig1c.pdf]

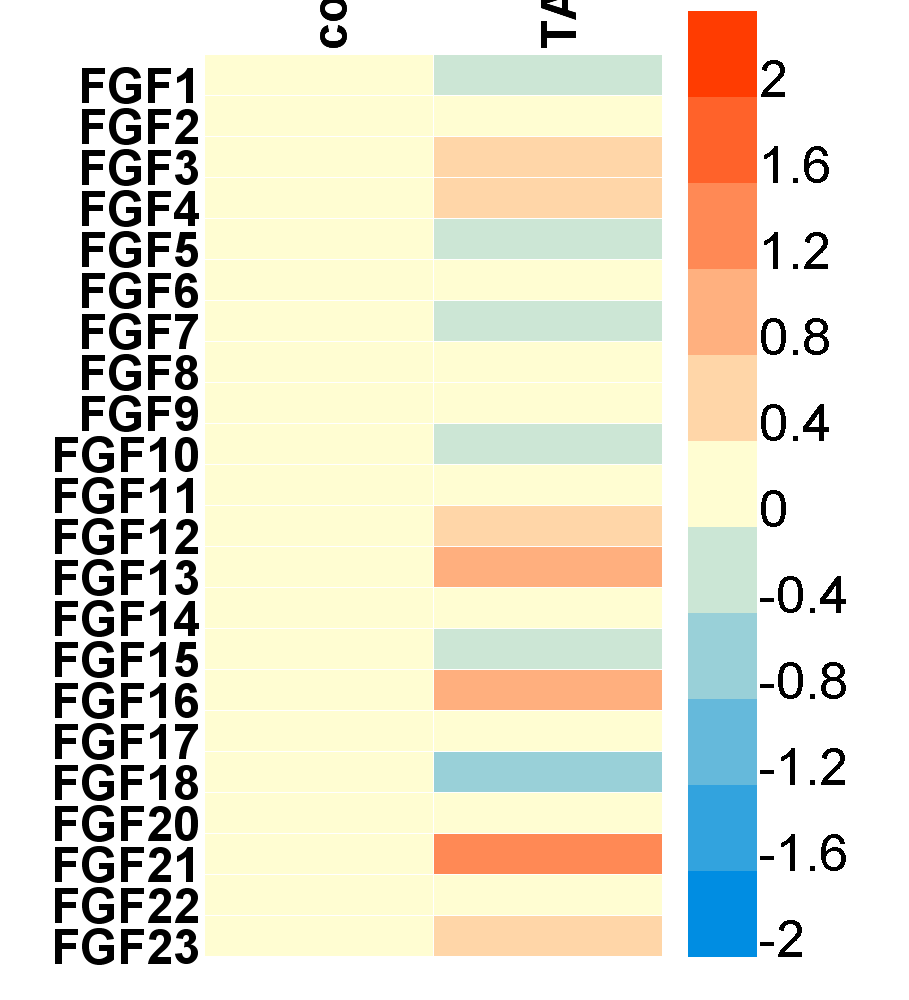

Supplement: Supplementary file 3 — Source Data [file 41467_2023_36895_MOESM3_ESM.zip › 22-09253B_Source Data file/F1/F1 d/heatmap sham TAC.tiff]

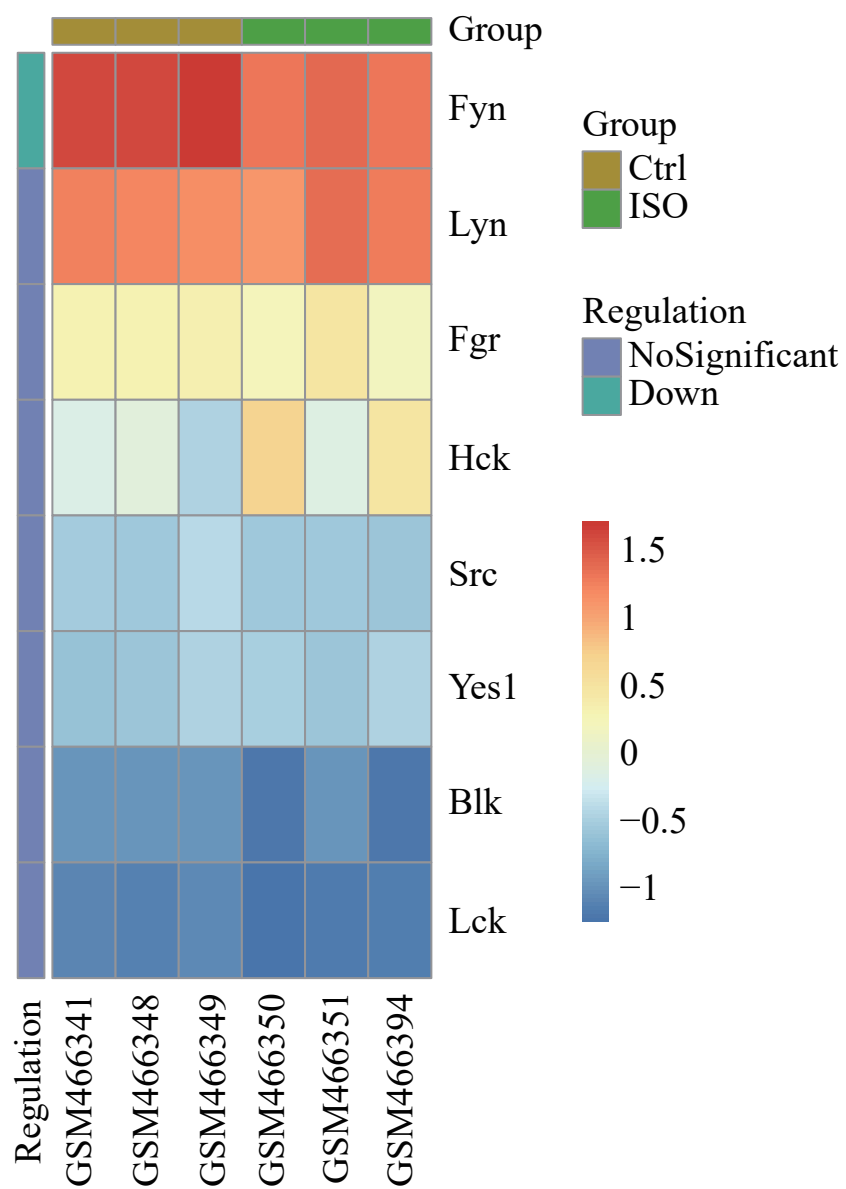

Supplement: Supplementary file 3 — Source Data [file 41467_2023_36895_MOESM3_ESM.zip › 22-09253B_Source Data file/F5/F5 a-b/Fig5 a.pdf]

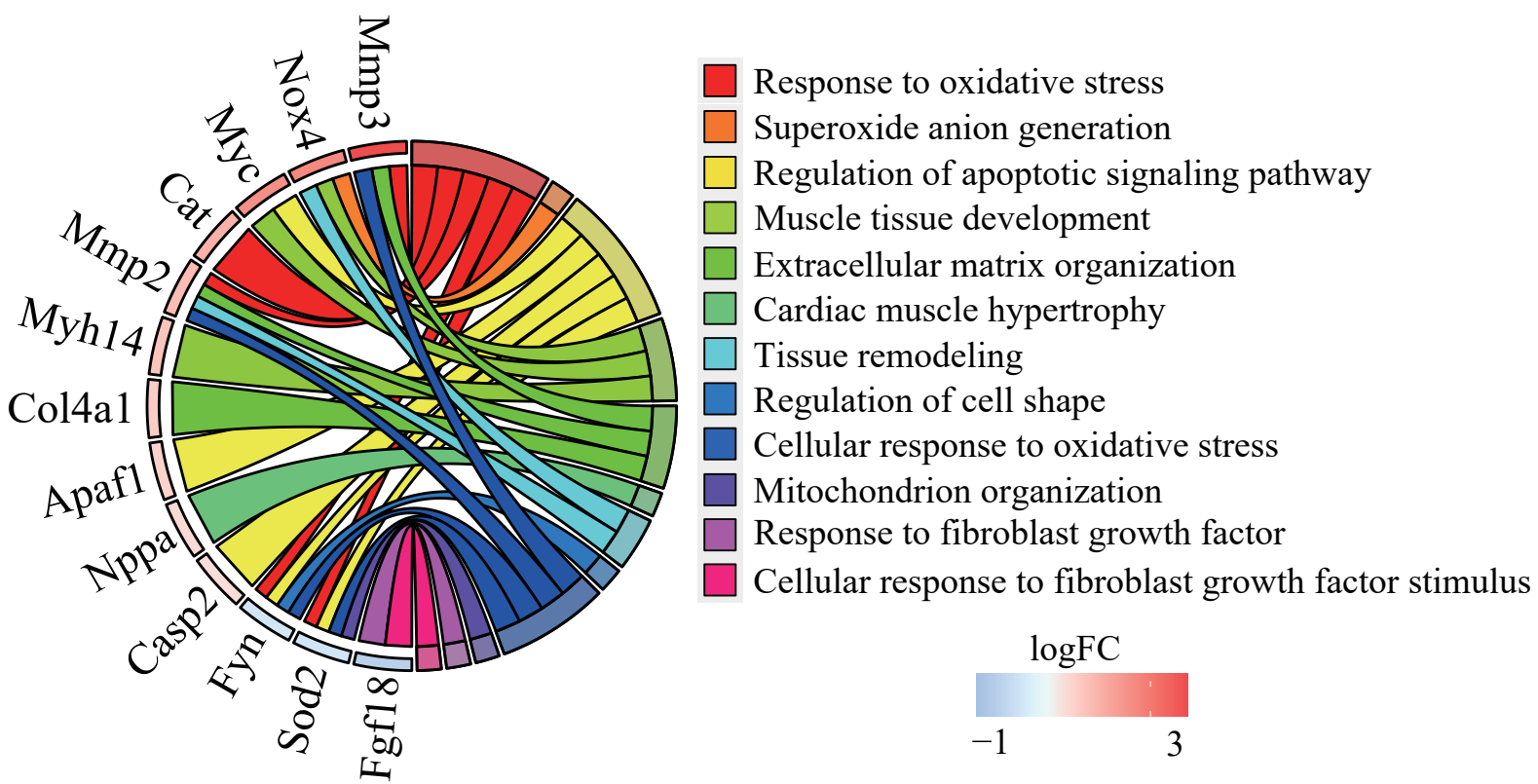

Supplement: Supplementary file 3 — Source Data [file 41467_2023_36895_MOESM3_ESM.zip › 22-09253B_Source Data file/F5/F5 a-b/Fig5 b.pdf]

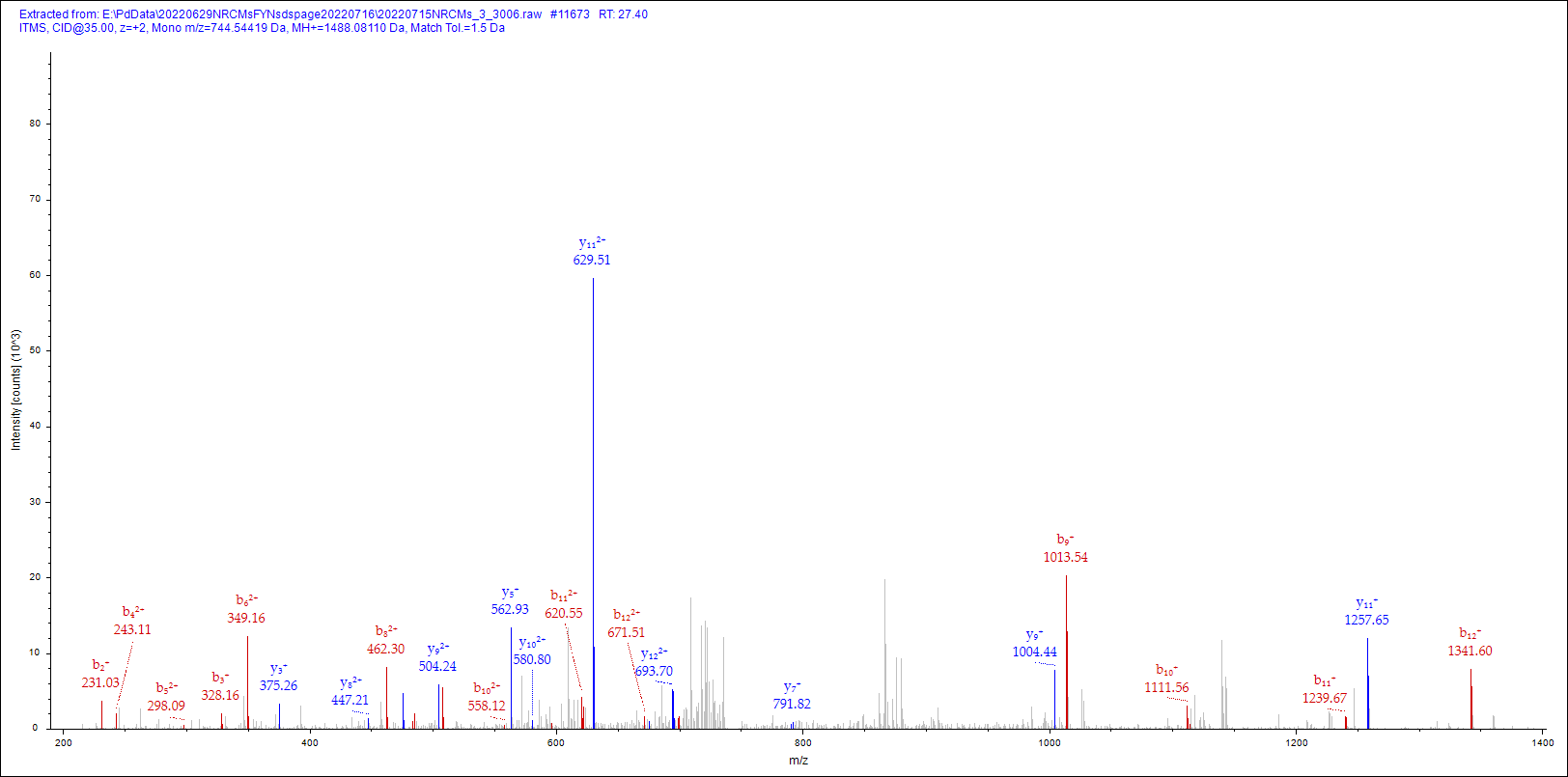

Supplement: Supplementary file 3 — Source Data [file 41467_2023_36895_MOESM3_ESM.zip › 22-09253B_Source Data file/F5/F5 c/1-1.tif]

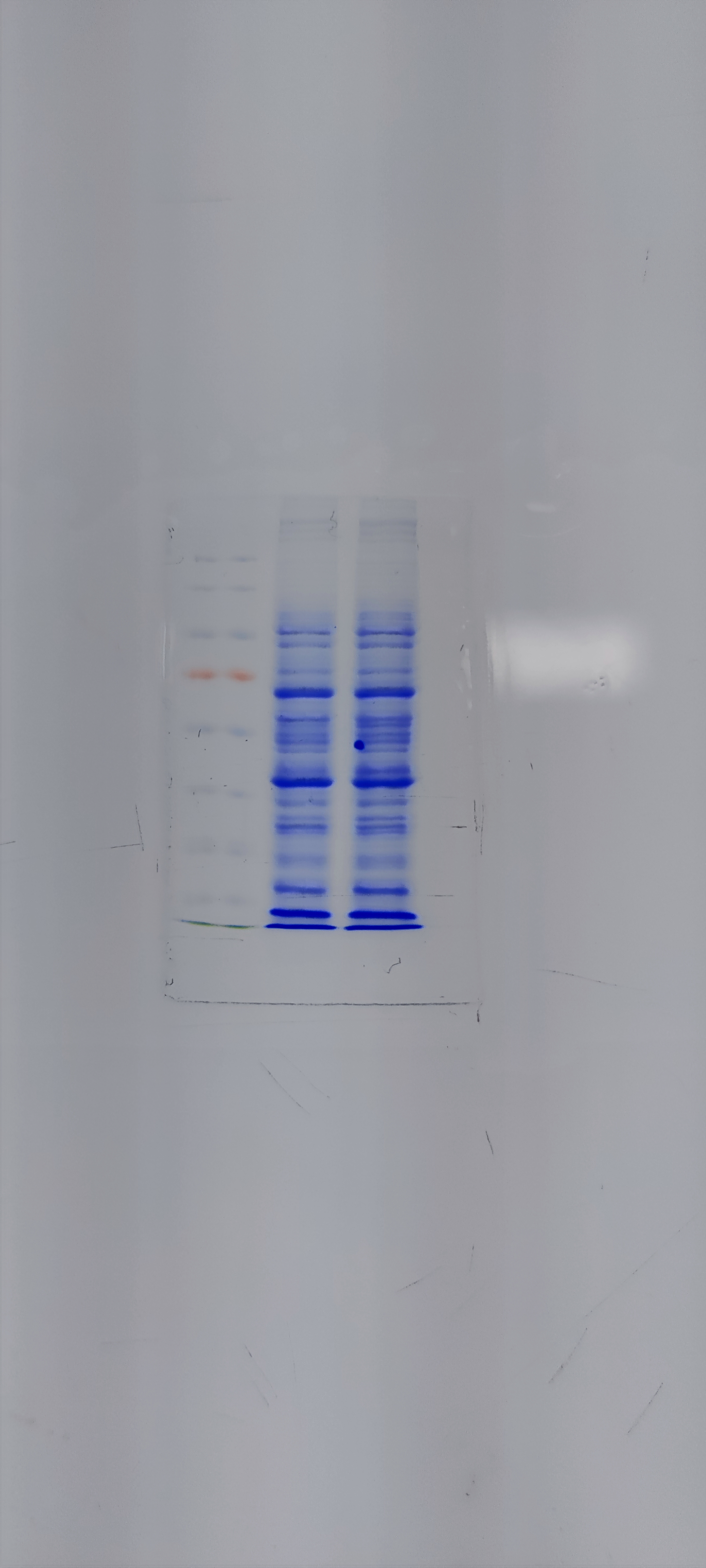

Supplement: Supplementary file 3 — Source Data [file 41467_2023_36895_MOESM3_ESM.zip › 22-09253B_Source Data file/F5/F5 c/NRCMs Gel.jpg]

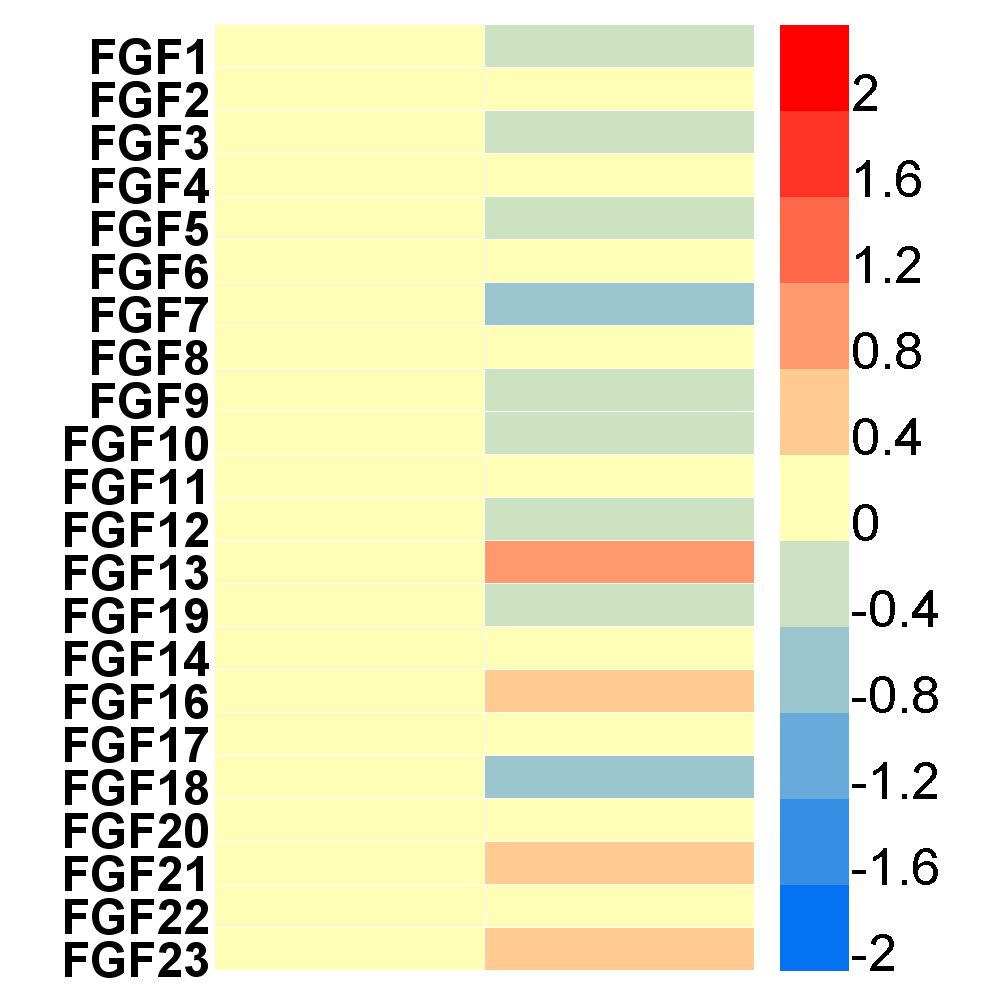

Supplement: Supplementary file 3 — Source Data [file 41467_2023_36895_MOESM3_ESM.zip › 22-09253B_Source Data file/S1/S1 c/heatmap.tiff]

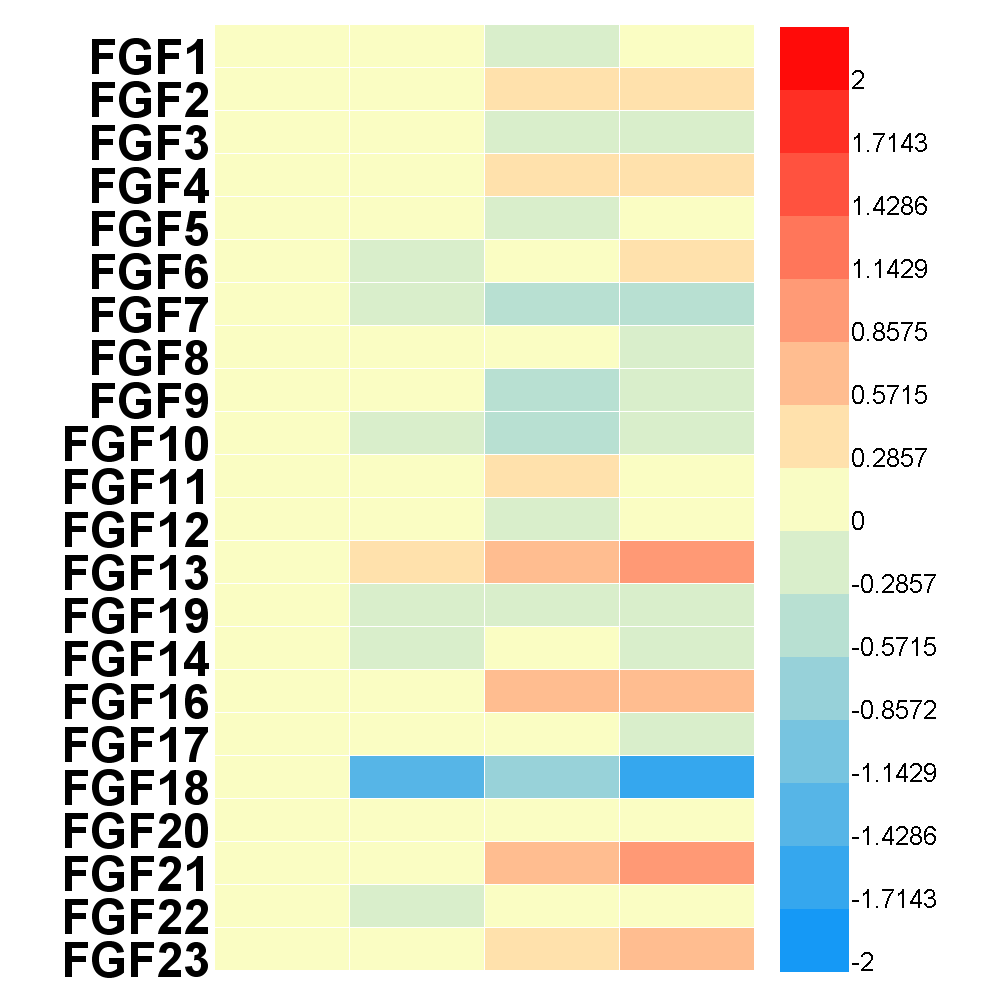

Supplement: Supplementary file 3 — Source Data [file 41467_2023_36895_MOESM3_ESM.zip › 22-09253B_Source Data file/S8/S8 f/heatmap.tiff]
